# LOXL2-induced PEAR1 Ser891 phosphorylation suppresses CD44 degradation and promotes triple-negative breast cancer metastasis

**DOI:** 10.1172/JCI177357

**Published:** 2024-08-15

**Authors:** Yingzhi Shen, Jie Yan, Lin Li, Huiyan Sun, Lin Zhang, Guoming Li, Xinxia Wang, Ruoyan Liu, Xuefeng Wu, Baosan Han, Xueqing Sun, Junling Liu, Xuemei Fan

**Affiliations:** 1 Department of Biochemistry and Molecular Cell Biology,; 2 Shanghai Institute of Immunology, Department of Immunology and Microbiology, and; 3 Department of General Surgery, Xinhua Hospital, Shanghai Jiao Tong University School of Medicine, Shanghai, China.; 4 Shanghai Synvida Biotechnology Co., Shanghai, China.

**Keywords:** Cell biology, Oncology, Breast cancer

## Abstract

CD44 is associated with a high risk of metastasis, recurrence, and drug resistance in various cancers. Here we report that platelet endothelial aggregation receptor 1 (PEAR1) is a CD44 chaperone protein that protected CD44 from endocytosis-mediated degradation and enhances cleavage of the CD44 intracellular domain (CD44-ICD). Furthermore, we found that lysyl oxidase–like protein 2 (LOXL2), an endogenous ligand of PEAR1, bound to the PEAR1-EMI domain and facilitated the interaction between PEAR1 and CD44 by inducing PEAR1 Ser891 phosphorylation in a manner that was independent of its enzyme activity. Levels of PEAR1 protein and PEAR1 phosphorylation at Ser891 were increased in patients with triple-negative breast cancer (TNBC), were positively correlated with expression of LOXL2 and CD44, and were negatively correlated with overall survival. The level of PEAR1 Ser891 phosphorylation was identified as the best independent prognostic factor in TNBC patients. The prognostic efficacy of the combination of PEAR1 phosphorylation at Ser891 and CD44 expression was superior to that of PEAR1 phosphorylation at Ser891 alone. Blocking the interaction between LOXL2 and PEAR1 with monoclonal antibodies significantly inhibited TNBC metastasis, representing a promising therapeutic strategy for TNBC.

## Abstract 2

Phosphorylation at Ser891 plays a decisive role in prometastatic function of PEAR1 by inhibiting the endocytic degradation of CD44 and promoting CD44-ICD signaling.

## Graphical abstract

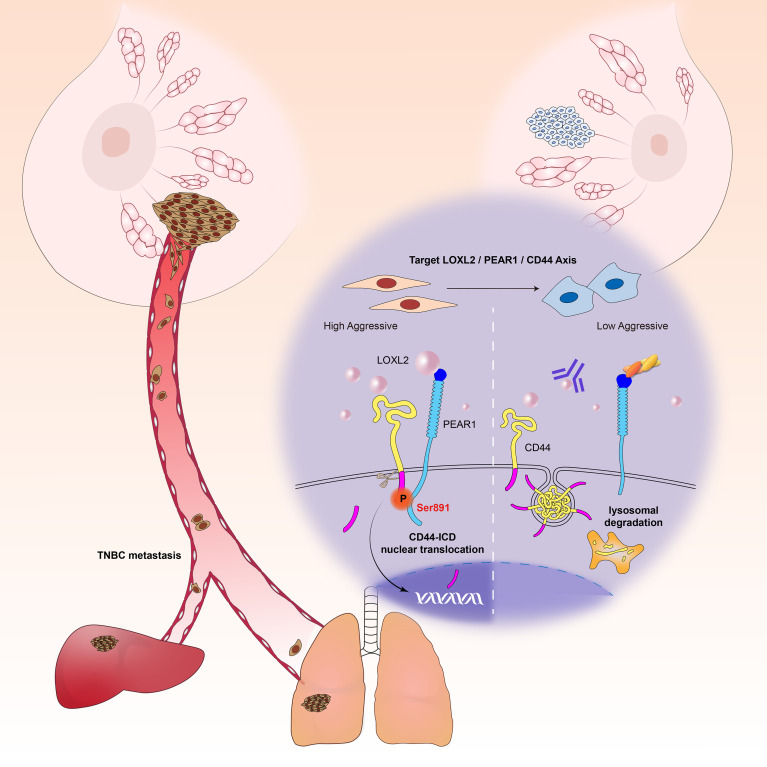

## Introduction

Breast cancer is the most prevalent malignancy in women, and its incidence is increasing (1). Of particular concern is triple-negative breast cancer (TNBC), which is an exceptionally aggressive and resistant subtype, with high heterogeneity and an unfavorable prognosis (2). TNBC is distinguished by the absence of estrogen receptor (ER), progesterone receptor (PR), and human epidermal growth factor receptor 2 (HER2) expression, which limits clinical treatment options (3). Thus, identification of novel therapeutic targets through comprehensive research on the molecular mechanisms underlying TNBC holds promise for the control of TNBC progression and enhancement of treatment success. Notably, cancer stem cells (CSCs), a rare population of potent self-renewing but poorly differentiated cells, account for drug resistance, tumor recurrence, and metastasis in TNBC (4). Remarkably, CD44-positive with absent or low CD24 (CD44^+^CD24^–/lo^) is the molecular marker profile most frequently used to identify TNBC stem cells (5, 6). Moreover, epithelial-mesenchymal transition (EMT) is enhanced in CSCs (7). Recently, the acquisition of both CSC properties and EMT phenotypes has garnered considerable attention as the pivotal driving force in cancer metastasis, and metastasis has emerged as the primary cause of mortality in patients with TNBC (8).

CD44 is highly expressed in advanced metastatic TNBC and acts as an important signal transduction control platform, contributing to tumorigenesis, progression, and metastasis (9). Specifically, the acknowledged ligand of CD44, hyaluronan, imparts conformational changes to CD44, thereby activating diverse signaling pathways that lead to cytoskeletal remodeling, cellular proliferation, and motility (10). A study showed that CD44 splice isoform switching regulates the breast cancer stem cell state, with the standard isoform (CD44s) responsible for stemness and the variant isoform (CD44v) responsible for proliferation (11). Furthermore, the CD44 intracellular domain (CD44-ICD) is cleaved by presenilin/γ-secretase and participates in the transcriptional regulation of stemness and EMT genes, notably *OCT4*, *SOX2*, *MMP9*, and *CD44* (12). Currently, therapeutic strategies targeting CD44, including CD44-neutralizing antibodies, RNA delivery, ectodomain mimics, and aptamers, are undergoing clinical development at various stages (13, 14). However, targeted therapy against CD44 faces clinical constraints, such as the complexity of CD44 variations and downstream signals coupled with tumor heterogeneity, the low affinity of CD44 monoclonal antibodies, the emergence of drug resistance, and severe skin toxicity due to the disruption of normal physiological functions (10, 15). Consequently, despite the promising therapeutic potential of CD44 as a tumor target, the above obstacles and limitations must be overcome before it can be applied in clinical treatment.

Platelet endothelial aggregation receptor 1 (PEAR1), a transmembrane receptor, was identified in 2005, and studies on PEAR1 have focused mainly on thrombosis and angiogenesis (16). Recently, we reported that PEAR1 plays an important role in fibroblast activation and pulmonary fibrosis through phosphatase 1 (17). Here we found that PEAR1 is a chaperone protein of CD44 that prevents CD44 from undergoing endocytosis-mediated degradation and maintains CD44 expression at high levels. PEAR1 plays a vital role in TNBC metastasis and serves as a biomarker for cancer prognosis. We also revealed that lysyl oxidase–like protein 2 (LOXL2), a well-known regulator of tumor, is an endogenous ligand binding to the PEAR1-EMI domain and facilitating PEAR1 Ser891 phosphorylation, which is essential for the binding of PEAR1 to CD44. Interestingly, antibodies blocking the interaction between LOXL2 and PEAR1 dramatically inhibited TNBC metastasis both in vitro and in vivo. Additionally, elevated expression levels of PEAR1, phosphorylated PEAR1 at Ser891, LOXL2, and CD44 in TNBC patients were strongly correlated with poor clinical outcomes, and these conditions significantly correlated with each other. Taken together, these findings indicate that the LOXL2/PEAR1/CD44 pathway is a promising target for TNBC therapy.

## Results

### PEAR1 is a CD44-associated protein that is correlated with poor survival in TNBC patients.

**Figure 1:**
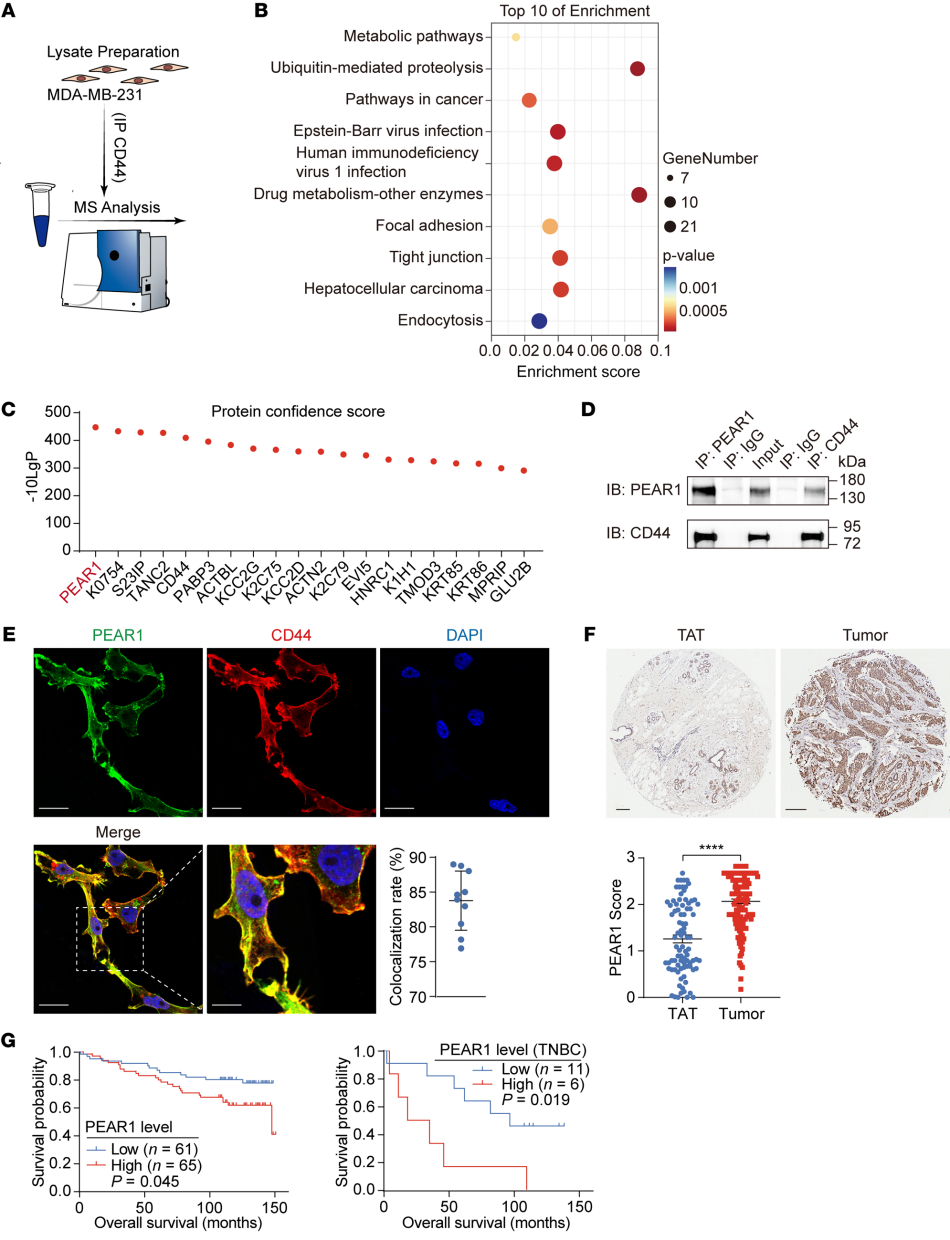
PEAR1 is a CD44-associated protein that is correlated with poor survival in TNBC patients. (**A**) Schematic of CD44-interacting proteins identified using IP-MS. CD44 and its interacting proteins were immunoprecipitated from whole-cell lysates of MDA-MB-231 cells using an anti-CD44 antibody and identified using MS. The IgG isotype served as a negative control. Specific proteins that interact with CD44 were screened. These proteins were detected in anti-CD44 IP samples but not in IgG IP samples; or the relative abundance ratio was greater than 200 in anti-CD44 IP samples compared to that in IgG-IP samples (area CD44/area IgG). (**B**) Bubble plots of the top 10 pathways obtained from KEGG enrichment analyses. (**C**) The top 20 effective proteins obtained from MS sorting according to the confidence score. (**D**) Co-IP of whole-cell lysates of MDA-MB-231 cells with anti-PEAR1 and anti-CD44 antibodies. The IgG isotype was used as a negative control. Results are representative of 3 independent experiments. (**E**) Representative IF staining of MDA-MB-231 cells with anti-PEAR1 (green) and anti-CD44 (red) antibodies and nuclei (blue). Scale bars: 20 μm (original image) and 10 μm (enlarged image). Quantitative analysis of the rate of PEAR1 and CD44 colocalization. (**F**) Representative IHC staining of the tissue microarray containing breast cancer and adjacent tissue samples with an anti-PEAR1 antibody. Scale bars: 50 μm. The PEAR1 staining scores were quantified as indicated (*n* = 86 for TATs, *n* = 126 for tumors; mean ± SEM). (**G**) Total overall survival of patients with breast cancer and with TNBC based on PEAR1 expression level (*n* values as indicated; log-rank test). KEGG pathway enrichment analyses were performed using the online tools DAVID and KOBAS (**B**); unpaired 2-tailed *t* tests were used for **F**; log-rank test was used for **G**. ****P < 0.0001.

Although CD44 is a well-known marker of CSCs and plays important roles in tumor initiation and development, the upstream and downstream regulatory mechanisms of CD44 remain unclear. First, proteins that interact with CD44 were coimmunoprecipitated with an anti-CD44 antibody and identified using mass spectrometry (MS) in the MDA-MB-231 cell line, which is a highly aggressive TNBC cell line that exhibits high CD44 expression levels (Figure 1A). Kyoto Encyclopedia of Genes and Genomes (KEGG) pathway enrichment analyses revealed that the proteins interacting with CD44 were mainly distributed in cancer-associated pathways, as expected, including pathways in involved in cancer, focal adhesion, tight junction, drug metabolism, and metabolic pathways. Moreover, the ubiquitin-mediated proteolysis and endocytosis pathways were also enriched (Figure 1B). The proteins were ranked in descending order based on the confidence score, and the top 20 proteins are listed in Figure 1C. Surprisingly, PEAR1 emerged as the foremost candidate binding partner of CD44 (Figure 1C and Supplemental Figure 1A; supplemental material available online with this article; https://doi.org/10.1172/JCI177357DS1). Subsequently, the association between PEAR1 and CD44 was confirmed by endogenous co-IP and immunofluorescence (IF) analyses in MDA-MB-231 cells (Figure 1, D and E, and Supplemental Figure 1B). Obvious colocalization of PEAR1 and CD44 was noted on the cancer cell membrane in the TNBC nest (Supplemental Figure 1C).

PEAR1 is a characteristic epidermal growth factor repeat–containing type 1 transmembrane receptor (16). PEAR1 participates in contact-induced platelet activation, neoangiogenesis, megakaryopoiesis, and fibroblast activation (17–21). However, investigations concerning the significance of PEAR1 in cancer remain scarce. To reveal the clinical relevance of PEAR1 expression, we performed microarray analysis of human breast tumor tissues alongside adjacent nontumor tissues using IHC staining of PEAR1. The results revealed a notable increase in PEAR1 expression levels in breast cancer tissues compared with tumor-adjacent tissues (TATs) (Figure 1F). Moreover, increased PEAR1 expression in patients with breast cancer was associated with poor overall survival, particularly in patients with TNBC (Figure 1G). Data pertaining to *PEAR1* mRNA expression in various breast cancer cell lines were obtained from the open-access Human Protein Atlas, which revealed that PEAR1 was expressed at high levels predominantly in TNBC cells (Supplemental Figure 1D).

### PEAR1 exacerbates TNBC cell metastasis.

**Figure 2:**
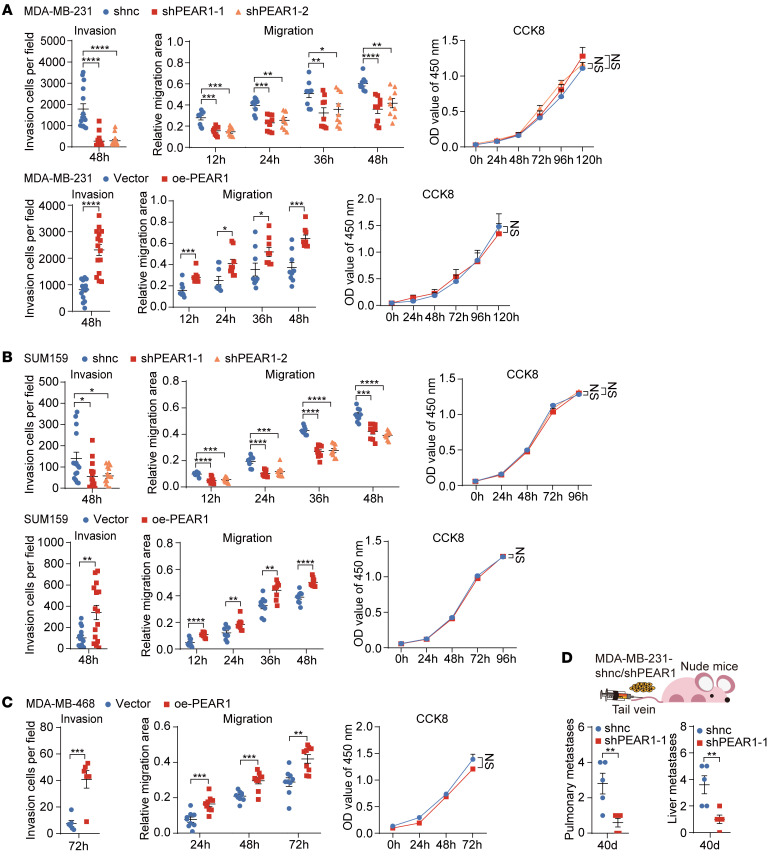
PEAR1 exacerbates TNBC cell metastasis. (**A**) MDA-MB-231 cells with stable PEAR1 knockdown (shPEAR1) or PEAR1 overexpression (oe-PEAR1) were generated and verified as shown in Supplemental Figure 2A. Nontargeting shRNA was used as a negative control for shPEAR1 (shnc). The corresponding empty vector (Vector) was used as a negative control for oe-PEAR1. Quantification of invasive shPEAR1- and oe-PEAR1–treated MDA-MB-231 cells in a Transwell assay; quantification of the relative migration area of shPEAR1- and oe-PEAR1–treated MDA-MB-231 cells in the wound healing assay; and viability curves of shPEAR1- and oe-PEAR1–treated MDA-MB-231 cells generated with a CCK-8 assay kit (*n* = 3; mean ± SEM). (**B**) Quantification of invasive shPEAR1- and oe-PEAR1–treated SUM159 cells in a Transwell assay; quantification of the relative migration area of shPEAR1- and oe-PEAR1–treated SUM159 cells in the wound healing assay; and viability curves of shPEAR1- and oe-PEAR1–treated SUM159 cells generated with a CCK-8 assay kit (*n* = 3; mean ± SEM). (**C**) Quantification of invasive oe-PEAR1–treated MDA-MB-468 cells in a Transwell assay; relative migration area of oe-PEAR1–treated MDA-MB-468 cells quantified using the wound healing assay; and viability of oe-PEAR1–treated MDA-MB-468 cells assessed by a CCK-8 assay kit (*n* = 3; mean ± SEM). (**D**) Quantification of metastatic foci in the lungs and livers of nude mice i.v. injected with shPEAR1 and shnc MDA-MB-231 cells by H&E staining (*n* = 5 mice per group; mean ± SEM). Unpaired 2-tailed *t* tests were used for **A**–**D**; 1-way ANOVA followed by Dunnett’s test was used for **A** and **B**. *P < 0.05, **P < 0.01, ***P < 0.001, ****P < 0.0001.

To evaluate the role of PEAR1 in TNBC cells, we generated stable PEAR1-knockdown (shPEAR1) and PEAR1-overexpression (oe-PEAR1) MDA-MB-231 and SUM159 cell lines using lentivirus infection, which we confirmed with quantitative real-time PCR (RT-qPCR) and Western blotting (Supplemental Figure 2, A and B). Given the relatively low expression of PEAR1 in MDA-MB-468 cells (Supplemental Figure 1D), only the oe-PEAR1 cell line was constructed in this cell type (Supplemental Figure 2C). Invasion, migration, and proliferation were investigated. The results of Transwell, wound healing, CCK-8, and EdU assays demonstrated that PEAR1 significantly augmented TNBC cell invasion and migration, with minimal effect on proliferation (Figure 2, A–C, and Supplemental Figure 2, A–C). Subsequently, the MDA-MB-231 cell xenograft nude mouse model was used to further elucidate the oncogenic roles of PEAR1 in TNBC in vivo. PEAR1 silencing attenuated lung and liver metastasis but had little effect on tumor growth (Figure 2D and Supplemental Figure 2, D and E).

Moreover, 4T1, a murine TNBC cell line, was also used to evaluate the carcinogenic function of PEAR1 in TNBC. Both in vitro and in vivo investigations suggested that Pear1 knockdown impeded 4T1 cell motility and metastasis, with no significant effect on proliferation, mirroring the outcomes obtained with human TNBC cells (Supplemental Figure 3, A–D). These data demonstrated that PEAR1 played a crucial role in the aggressive phenotypes of TNBC.

### PEAR1 protects CD44 from endocytosis-mediated degradation.

**Figure 3:**
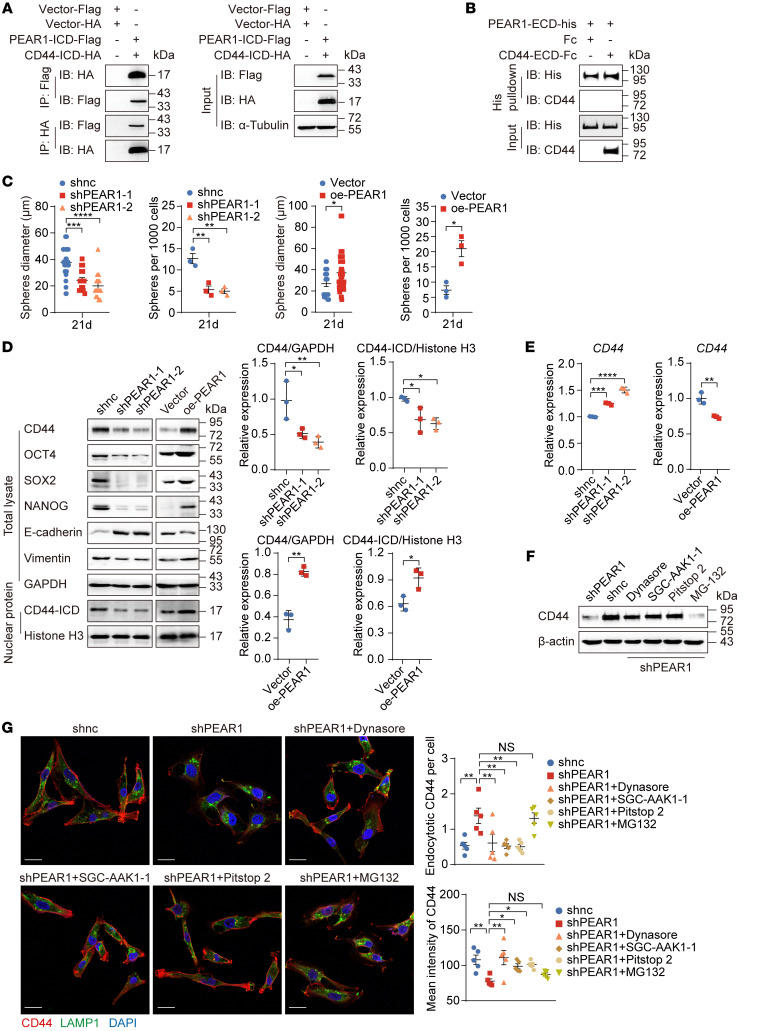
PEAR1 protects CD44 from endocytosis-mediated degradation. (**A**) Co-IP of whole-cell lysates of HEK293T cells transiently transfected with PEAR1-ICD–Flag and CD44-ICD–HA plasmids with anti-Flag and anti-HA agarose beads. Cells transfected with the corresponding empty vector plasmids were used as negative controls. (**B**) The interaction between PEAR1-ECD and CD44-ECD was detected using pulldown with anti-His Dynabeads in a solution containing purified PEAR1-ECD–His and CD44-ECD–Fc protein. Fc was used as a negative control. (**C**) Quantification of the mammosphere diameters and quantities of shPEAR1 and oe-PEAR1 MDA-MB-231 cells (*n* = 3; mean ± SEM). (**D**) Expression levels of CD44, OCT4, SOX2, NANOG, E-cadherin, and vimentin in total lysates and of CD44-ICD in nuclear proteins of shPEAR1 and oe-PEAR1 MDA-MB-231 cells as determined by Western blotting analysis. Quantitative analysis of the relative gray values of full-length CD44 with GAPDH and nuclear CD44-ICD with histone H3 (*n* = 3; mean ± SD). (**E**) *CD44* mRNA expression levels in shPEAR1 and oe-PEAR1 MDA-MB-231 cells were determined by RT-qPCR. The diagram shows the relative expression of mRNAs normalized to that of *18S* rRNA (*n* = 3; mean ± SD). (**F** and **G**) Western blotting of CD44 (**F**) and IF staining of CD44 (red), LAMP1 (green), and nuclei (blue) (**G**) in shPEAR1 MDA-MB-231 cells treated with or without dynasore, SGC-AAK-1, pitstop 2, or MG132 for 1 hour. β-Actin served as an internal control (**F**). Number of endocytotic CD44 foci per cell and mean intensity of CD44 (**G**). Scale bars: 20 μm. One-way ANOVA followed by Dunnett’s test was used for **C**–**E** and **G**; unpaired 2-tailed *t* tests were used for **C**–**E**. *P < 0.05, **P < 0.01, ***P < 0.001, ****P < 0.0001. The Western blotting results are representative of 3 independent experiments.

To further determine the mode of interaction between PEAR1 and CD44, we expressed the ICD and extracellular domain (ECD) of both receptors separately. The results showed that the specific binding region was present in the ICD but not in the ECD (Figure 3, A and B). Next, the biological function of the PEAR1 and CD44 interaction was further investigated in oe-PEAR1 and shPEAR1 MDA-MB-231 cells. We observed significant inhibition of mammosphere formation; expression of the stemness proteins OCT4, SOX2, and NANOG; and expression of the mesenchymal marker vimentin in shPEAR1 MDA-MB-231 cells, whereas expression of the epithelial marker E-cadherin was significantly increased. The opposite trend was observed in oe-PEAR1 MDA-MB-231 cells (Figure 3, C and D, and Supplemental Figure 4A). These data indicated that the stemness and EMT properties of MDA-MB-231 cells were regulated by PEAR1, which was consistent with the typical function of CD44. In fact, PEAR1 directly affected CD44 protein expression levels in MDA-MB-231 cells and SUM159 cells (Figure 3D and Supplemental Figure 4B). Considering that the *CD44* mRNA levels were inconsistent with its protein levels (Figure 3E and Supplemental Figure 4C), we evaluated the degradation pathway. We found that the decrease in CD44 protein levels induced by PEAR1 knockdown could be effectively reversed by endocytosis inhibitors containing dynasore (an inhibitor of dynamin), SGC-AAK1-1 (an inhibitor of AP2-associated kinase 1 [AAK1]) and pitstop 2 (an inhibitor of clathrin), whereas MG132 (an inhibitor of the proteasome) had no effect (Figure 3F). IF staining revealed that CD44 did not localize to the membrane but entered the lysosome under PEAR1-knockdown conditions. Inhibition of endocytosis restored the membrane localization of CD44, whereas inhibition of proteasome-mediated degradation was ineffective (Figure 3G). These data suggested that PEAR1 is a chaperone protein of CD44 and protects it from endocytosis-mediated degradation.

CD44-ICD is cleaved by presenilin/γ-secretase and then released into the nucleus to participate in gene transcription regulation (12). We found that CD44-ICD levels in the nucleus were also regulated by PEAR1 (Figure 3D and Supplemental Figure 4B). DAPT, an inhibitor of γ-secretase, inhibited CD44-ICD cleavage (Supplemental Figure 4D). MDA-MB-231 cell invasion and migration were significantly suppressed by DAPT in a dose-dependent manner, but DAPT did not affect cell proliferation (Supplemental Figure 4, E–G). These data suggested that CD44-ICD was an important messenger of CD44-mediated intracellular signals and that PEAR1 played important roles in maintaining the stability of CD44 on the cell membrane and facilitating CD44-ICD cleavage by γ-secretase.

Interestingly, PEAR1 overexpression failed to enhance the invasion, migration, and proliferation of the weakly invasive MCF7 cell line (Supplemental Figure 5, A–D), which might be attributable to the lack of CD44 expression in MCF7 cells (Supplemental Figure 5E). MDA-MB-231 cell invasion and migration were inhibited by CD44 knockdown, whereas proliferation was not affected, which was consistent with PEAR1 deficiency. Moreover, the enhanced role of PEAR1 was inhibited by CD44 knockdown in MDA-MB-231 cells (Supplemental Figure 5, F–I). These data indicated that PEAR1 regulated TNBC cell metastasis mainly through CD44.

### PEAR1 phosphorylation at Ser891 is crucial for CD44 function.

**Figure 4:**
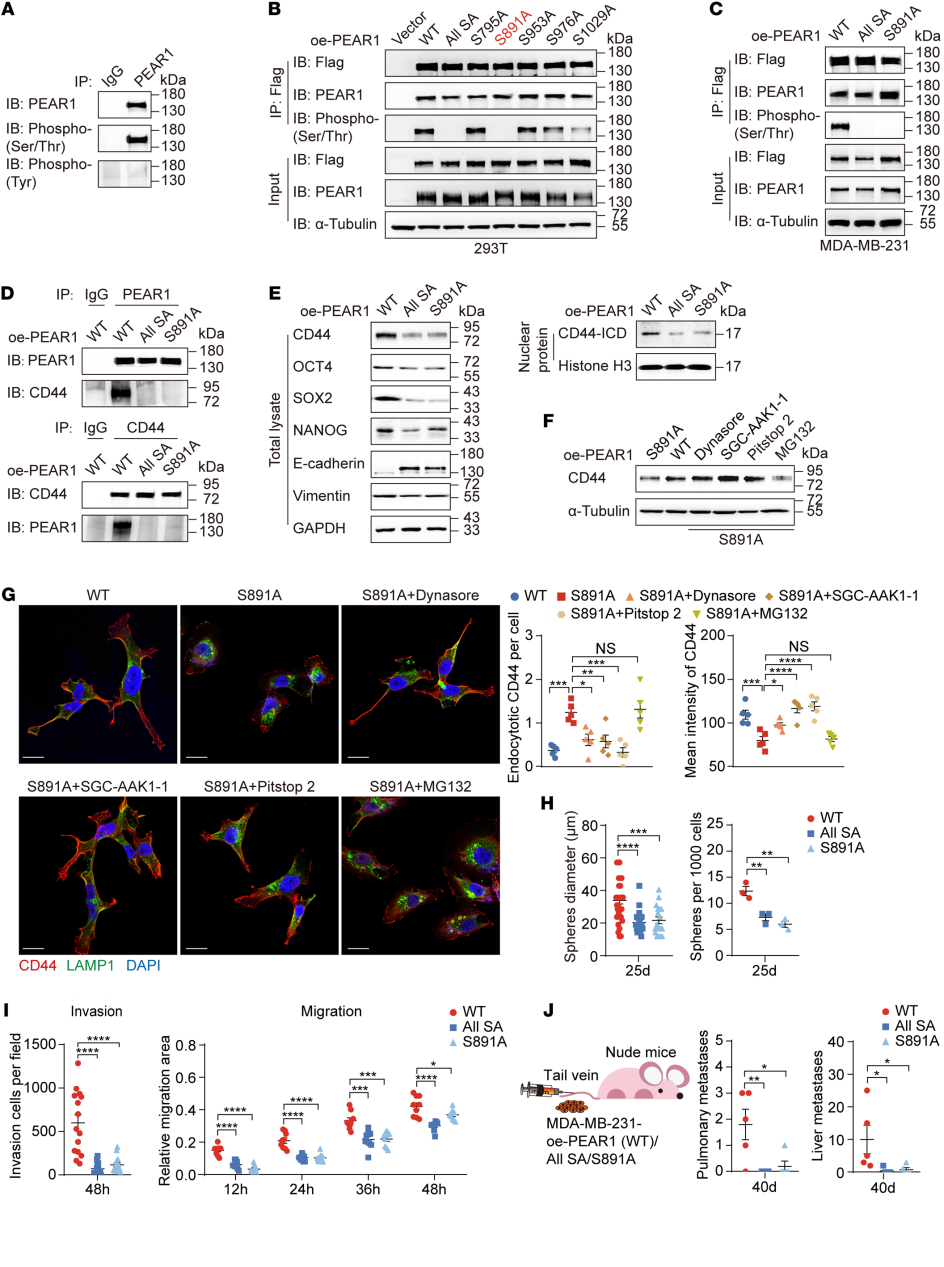
PEAR1 phosphorylation at Ser891 is crucial for CD44 function. (**A**) PEAR1 was immunoprecipitated from MDA-MB-231 cell lysates with anti-PEAR1 antibody, after which the levels of serine/threonine phosphorylation or tyrosine phosphorylation were measured with Western blotting. IgG served as an isotype control. (**B**) Serine/threonine phosphorylation levels of PEAR1 in HEK293T cells transiently transfected with oe-PEAR1–WT-Flag, oe-PEAR1–All SA-Flag, oe-PEAR1–S795A-Flag, oe-PEAR1–S891A-Flag, oe-PEAR1–S953A-Flag, oe-PEAR1–S976A-Flag, or oe-PEAR1–S1029A-Flag plasmids. “All SA” indicates that all serine was mutated to alanine at all 5 residues. (**C**) PEAR1 serine/threonine phosphorylation levels in MDA-MB-231 cells stably expressing PEAR1-WT-Flag, SA-Flag, or S891A-Flag. (**D**) The interaction between PEAR1 and CD44 in MDA-MB-231 cells was detected by co-IP with anti-PEAR1 or anti-CD44 antibodies. (**E**) CD44, OCT4, SOX2, NANOG, E-cadherin, vimentin, and CD44-ICD protein expression in total lysates and nuclear fractions of the indicated MDA-MB-231 cells. (**F** and **G**) Western blotting of CD44 (**F**) and IF staining of CD44 (red), LAMP1 (green), and nuclei (blue) (**G**) in oe-PEAR1–WT-Flag and oe-PEAR1–S891A-Flag MDA-MB-231 cells treated with or without dynasore, SGC-AAK-1, pitstop 2, or MG132 for 1 hour. Number of endocytotic CD44 foci per cell and mean intensity of CD44. Scale bars: 20 μm. (**H** and **I**) Quantification of mammosphere diameters and quantities (**H**); and quantification of invasive cells in Transwell assay and relative migration area in wound healing assay (**I**) of the indicated MDA-MB-231 cells (*n* = 3; mean ± SEM). (**J**) Quantification of metastatic foci by H&E staining in the lungs and livers of nude mice i.v. injected with the indicated MDA-MB-231 cells (*n* = 5 mice per group; mean ± SEM). One-way ANOVA followed by Dunnett’s test was used for **G**–**J**. *P < 0.05, **P < 0.01, ***P < 0.001, ****P < 0.0001. The Western blotting results are representative of 3 independent experiments.

Previous studies have reported that PEAR1 is tyrosine phosphorylated in response to ligand binding during platelet aggregation (22). However, we found that PEAR1 underwent serine/threonine phosphorylation, not tyrosine phosphorylation, in MDA-MB-231 cells (Figure 4A). Furthermore, PEAR1 was subject to be pulled down, and PEAR1 phosphorylation was identified by MS. There were 5 serine sites phosphorylated in PEAR1-ICD, but only the S891A mutation completely blocked PEAR1 phosphorylation, indicating that Ser891 was the key phosphorylation site of PEAR1 (Figure 4B and Supplemental Figure 6A).

To evaluate the function of Ser891, we established MDA-MB-231 cell lines stably overexpressing a PEAR1 S891A mutation or a total of 5 site mutations (all serine>alanine) (Figure 4C). The data showed that the S891A mutation deprived PEAR1 of its ability to interact with CD44, maintained CD44 function, and protected CD44 from endocytosis-mediated degradation (Figure 4, D–G, and Supplemental Figure 6B). In addition, the enhanced role of PEAR1 in mammosphere formation, invasion, and migration also disappeared with the S891A mutation both in vitro and in vivo (Figure 4, H–J, and Supplemental Figure 6, C–F). These data demonstrated that the functions of PEAR1 in TNBC metastasis depended on its phosphorylation at Ser891.

Furthermore, the kinase that induces PEAR1 Ser891 phosphorylation was identified by co-IP and MS. WNK1, a lysine-deficient protein kinase 1, is a member of the serine-threonine protein kinase family and enhances migration and invasion in breast cancer (23). The interaction between WNK1 and PEAR1 was detected via MS of PEAR1-binding proteins and confirmed by co-IP (Supplemental Figure 6, G and H). WNK-IN-11, an allosteric inhibitor of WNK1, inhibited PEAR1 serine phosphorylation and suppressed MDA-MB-231 cell invasion and migration, with little effect on proliferation (Supplemental Figure 6, I–L). The results indicated that WNK1 was the kinase responsible for Ser891 phosphorylation of PEAR1.

### LOXL2, an endogenous ligand of PEAR1, triggers PEAR1 phosphorylation.

**Figure 5:**
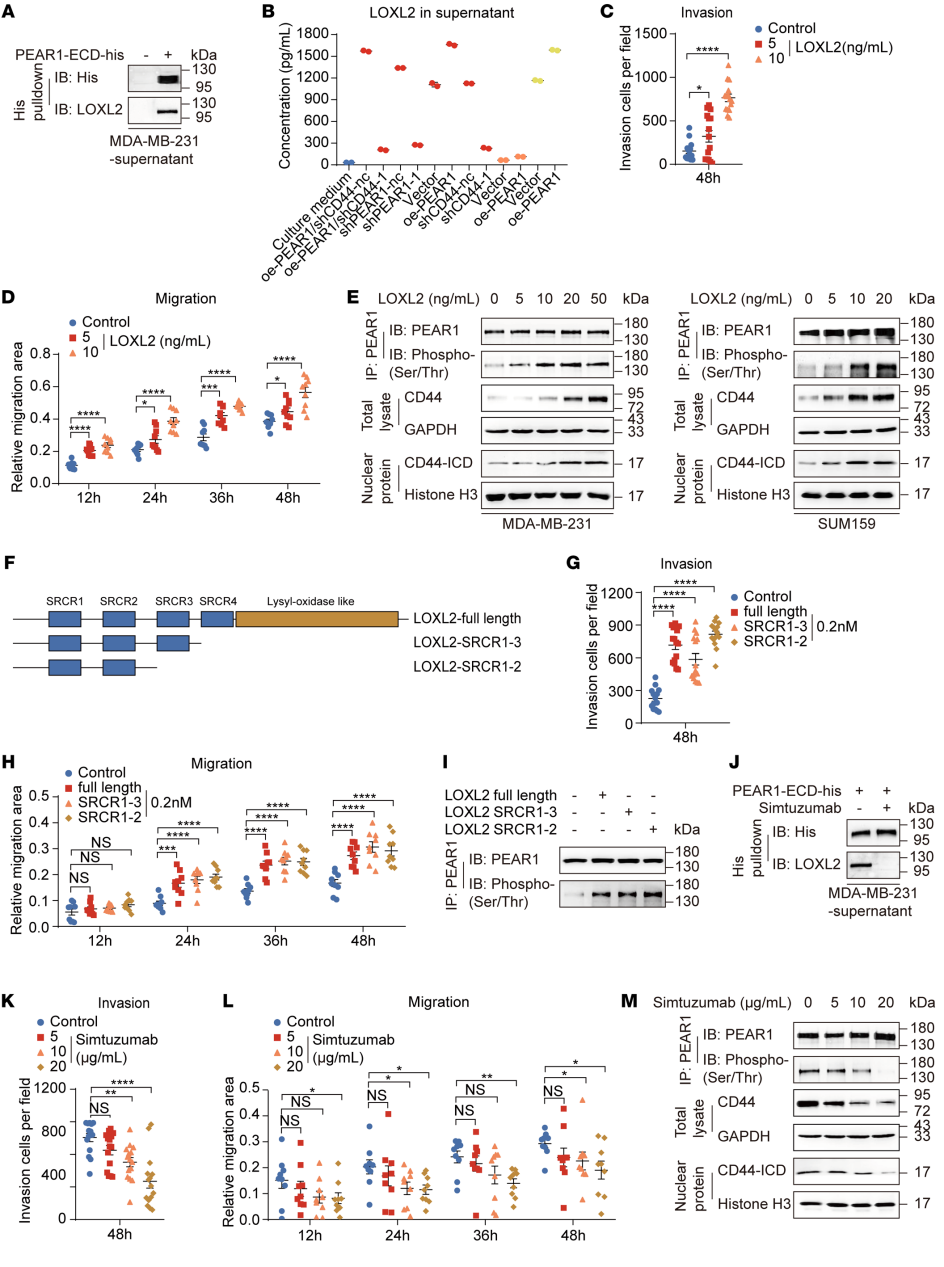
LOXL2 triggers PEAR1 phosphorylation. (**A**) The interaction between LOXL2 and PEAR1 was detected by pulldown with Dynabeads in the supernatant of MDA-MB-231 cells, to which 15 μg exogenous PEAR1-ECD-his was added. (**B**) LOXL2 levels in the supernatants of various TNBC cell lines were quantified by ELISA (MDA-MB-231, red; MDA-MB-468, orange; SUM159, yellow). The complete culture medium served as a negative control (blue) (*n* = 2; mean ± SD). (**C** and **D**) Quantification of invasive cells in Transwell assay; and relative migration area in wound healing assay of MDA-MB-231 cells treated with 0, 5, or 10 ng/mL LOXL2 (*n* = 3; mean ± SEM). (**E**) Phosphorylated PEAR1, full-length CD44, and CD44-ICD levels were detected in MDA-MB-231 cells and SUM159 cells treated with 0, 5, 10, 20, or 50 ng/mL LOXL2. (**F**) Schematic showing the structures of full-length LOXL2 protein, the LOXL2-SRCR1-3 truncation, and the LOXL2-SRCR1-2 truncation. (**G**–**I**) Quantification of invasive cells in Transwell assay and relative migration area in wound healing assay (*n* = 3; mean ± SEM) and detection of PEAR1 phosphorylation levels with Western blotting in MDA-MB-231 cells treated with 0.2 nM full-length LOXL2 or its truncation construct. (**J**) Interaction of PEAR1-ECD–His with LOXL2 in the supernatant of MDA-MB-231 cells was inhibited by 20 μg/mL simtuzumab. (**K** and **L**) Quantification of invasive cells in Transwell assay and relative migration area in wound healing assay in MDA-MB-231 cells treated with 0, 5, 10, or 20 μg/mL simtuzumab (*n* = 3; mean ± SEM). (**M**) Levels of phosphorylated PEAR1, CD44, and CD44-ICD were determined in MDA-MB-231 cells treated with 0, 5, 10, or 20 μg/mL simtuzumab. One-way ANOVA followed by Dunnett’s test was used for **C**, **D**, **G**, **H**, **K**, and **L**. *P < 0.05, **P < 0.01, ***P < 0.001, ****P < 0.0001. The Western blotting results are representative of 3 independent experiments.

Ligand binding is essential for receptor activation and intracellular signal transduction (22, 24). To identify the ligands of PEAR1, we pulled down the supernatant of MDA-MB-231 cells with PEAR1-ECD protein. Interestingly, LOXL2, which is a participant in the catalysis and cross-linking of the extracellular matrix (ECM) and a prognostic marker of various tumors (25), was enriched (Figure 5A and Supplemental Figure 7, A and B). LOXL2 is an autocrine factor in TNBC cells, and LOXL2 secretion was positively correlated with PEAR1 and CD44 expression levels in MDA-MB-231 cells (Figure 5B). Addition of exogenous LOXL2 induced PEAR1 phosphorylation and enhanced TNBC cell invasion and migration in a dose-dependent manner without affecting proliferation (Figure 5, C–E, and Supplemental Figure 7, C and D). The functions of the full-length LOXL2 protein, SRCR1-3 protein (lysyl oxidase–like region and SRCR4 domain deletion), and SRCR1-2 protein (lysyl oxidase–like region, SRCR4 domain, and SRCR3 domain deletion) were comparable (Figure 5, F–I, and Supplemental Figure 7, E and F). These results suggested that the ability of LOXL2 to promote TNBC cell invasion and migration was dependent on the presence of the SRCR1-2 domain, which lacks enzyme activity. Simtuzumab, which is an allosteric inhibitory monoclonal antibody against LOXL2 (26), suppressed the interaction between LOXL2 and PEAR1 and PEAR1 phosphorylation and inhibited MDA-MB-231 cell migration and invasion (Figure 5, J–M, and Supplemental Figure 7, G and H). Furthermore, expression levels of full-length CD44 and nuclear CD44-ICD, which are downstream effectors of PEAR1, also changed with the binding of LOXL2 to PEAR1 (Figure 5, E and M). These results suggested that LOXL2, an endogenous ligand of PEAR1, contributed to TNBC cell invasion and migration by activating PEAR1 phosphorylation and downstream CD44 signaling.

### Blocking the interaction of LOXL2 with PEAR1 inhibits TNBC metastasis.

**Figure 6:**
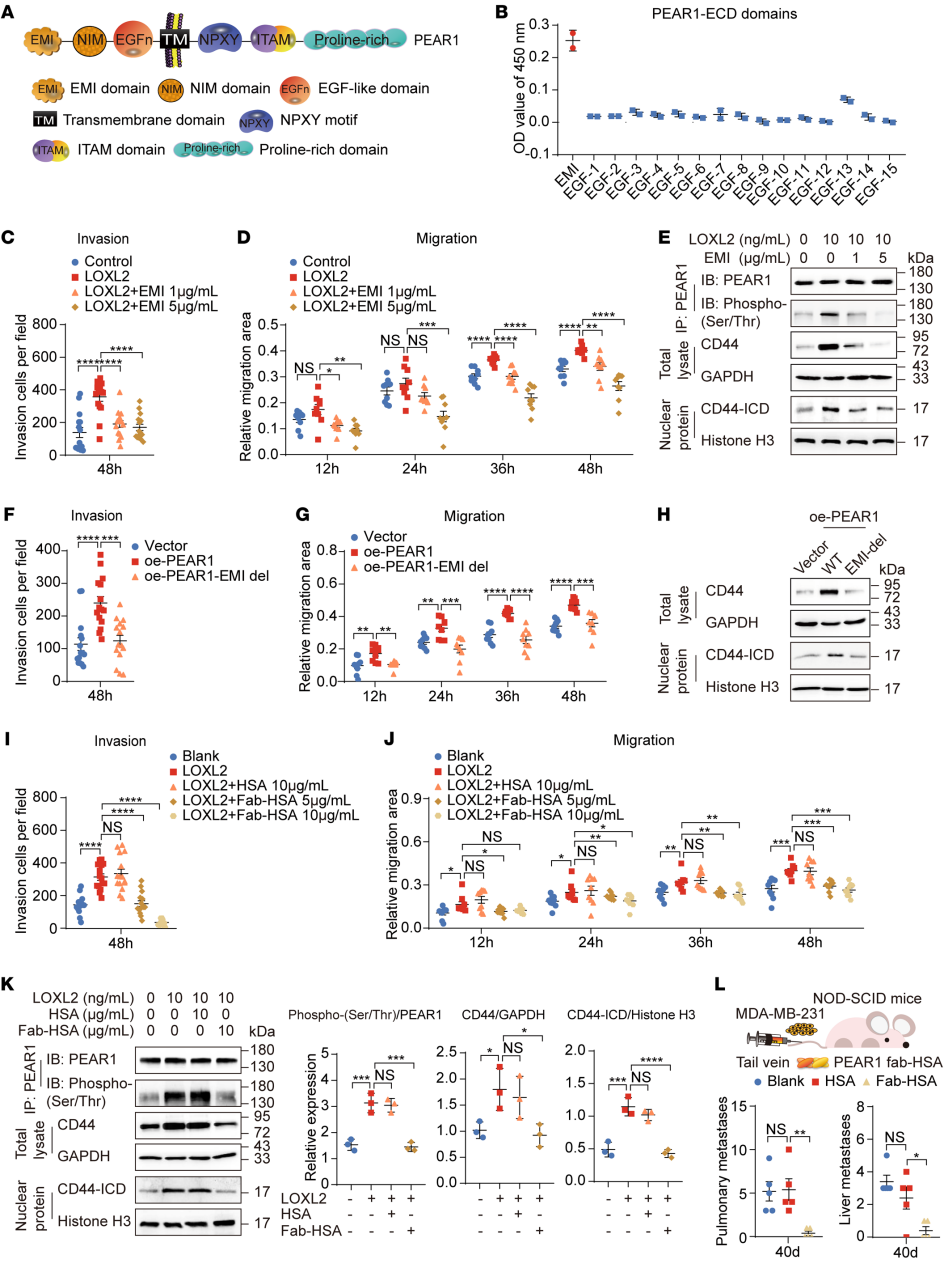
Blocking the interaction of LOXL2 with PEAR1 inhibits TNBC metastasis. (**A**) Schematic showing the structural features of PEAR1. (**B**) The interaction between LOXL2 and various PEAR1-ECD domain peptides was detected using ELISA (*n* = 2; mean ± SD). (**C**–**E**) Quantification of invasive cells in Transwell assay; quantification of relative migration area in wound healing assay (*n* = 3; mean ± SEM); detection of phosphorylated PEAR1, CD44, and CD44-ICD using Western blotting of MDA-MB-231 cells treated with or without 10 ng/mL LOXL2 and 1 or 5 μg/mL PEAR1-EMI domain protein. EMI del, EMI deletion. (**F**–**H**) Quantification of invasive cells in Transwell assay; quantification of relative migration area in wound healing assay (*n* = 3; mean ± SEM); and detection of CD44 and CD44-ICD by Western blotting of MDA-MB-231 cells with oe-PEAR1-EMI domain deficiency. EMI del, EMI deletion. (**I**–**K**) Quantification of invasive cells in Transwell assay; quantification of relative migration area in wound healing assay (*n* = 3; mean ± SEM); and detection of phosphorylated PEAR1, CD44, and CD44-ICD using Western blotting of MDA-MB-231 cells treated with 10 ng/mL LOXL2 and 5 or 10 μg/mL PEAR1 Fab-HSA. Quantitative analysis of the gray values of phospho–PEAR1 Ser/Thr to PEAR1, CD44 to GAPDH, and CD44-ICD to histone H3 (*n* = 3; mean ± SD). (**L**) The number of metastatic foci in the lung and liver was significantly reduced by i.v. injection of PEAR1 Fab-HSA at a dose of 3.35 mg/kg for 40 days in a mouse model of metastasis generated by i.v. injection of MDA-MB-231 cells (*n* = 5 mice per group; mean ± SEM). One-way ANOVA followed by Dunnett’s test was used for **C**, **D**, **F**, **G**, and **I**–**L**. *P < 0.05, **P < 0.01, ***P < 0.001, ****P < 0.0001. The Western blotting results are representative of 3 independent experiments.

The PEAR1-ECD is predominantly composed of an EMI domain and 15 EGF-like repeats (Figure 6A). ELISA results showed that LOXL2 bound mainly to the EMI domain of PEAR1 (Figure 6B). The recombinant EMI protein impaired serine phosphorylation of PEAR1 and the invasion and migration of TNBC cells induced by LOXL2 but had no significant effect on cell proliferation (Figure 6, C–E, and Supplemental Figure 8, A and B). Furthermore, in the context of increased CD44 and CD44-ICD expression, TNBC cell invasion and migration were blocked by deletion of the PEAR1-EMI domain (Figure 6, F–H, and Supplemental Figure 8, C and D). The results showed that the EMI domain of PEAR1 was crucial for LOXL2 binding and played important roles in TNBC metastasis. Inspired by these results, we developed a monoclonal antibody targeting the PEAR1-EMI domain. To avoid the possibility of PEAR1 clustering caused by the 2 fragment antigen-binding (Fab) arms of the normal antibody and to prolong the lifespan of the antibody in vivo, we coupled 1 Fab of the antibody to human serum albumin (HSA) and obtained Fab-HSA targeting the PEAR1-EMI domain (Supplemental Figure 8E). Fab-HSA blocked the binding of PEAR1 to LOXL2 and effectively inhibited the invasion and migration of MDA-MB-231 cells induced by LOXL2 in vitro (Figure 6, I and J, and Supplemental Figure 8, F–H). Mechanistically, Fab-HSA inhibited PEAR1 serine phosphorylation, downstream CD44 stability, and CD44-ICD signaling (Figure 6K). Encouragingly, lung and liver metastases were dramatically suppressed upon the administration of Fab-HSA in vivo in a NOD-SCID TNBC metastasis model (Figure 6L and Supplemental Figure 8I). These results demonstrated that targeting the LOXL2/PEAR1/CD44 axis with monoclonal antibodies is a promising strategy for TNBC treatment.

### The LOXL2/PEAR1/CD44 axis is upregulated in TNBC and is associated with worse overall survival.

**Figure 7:**
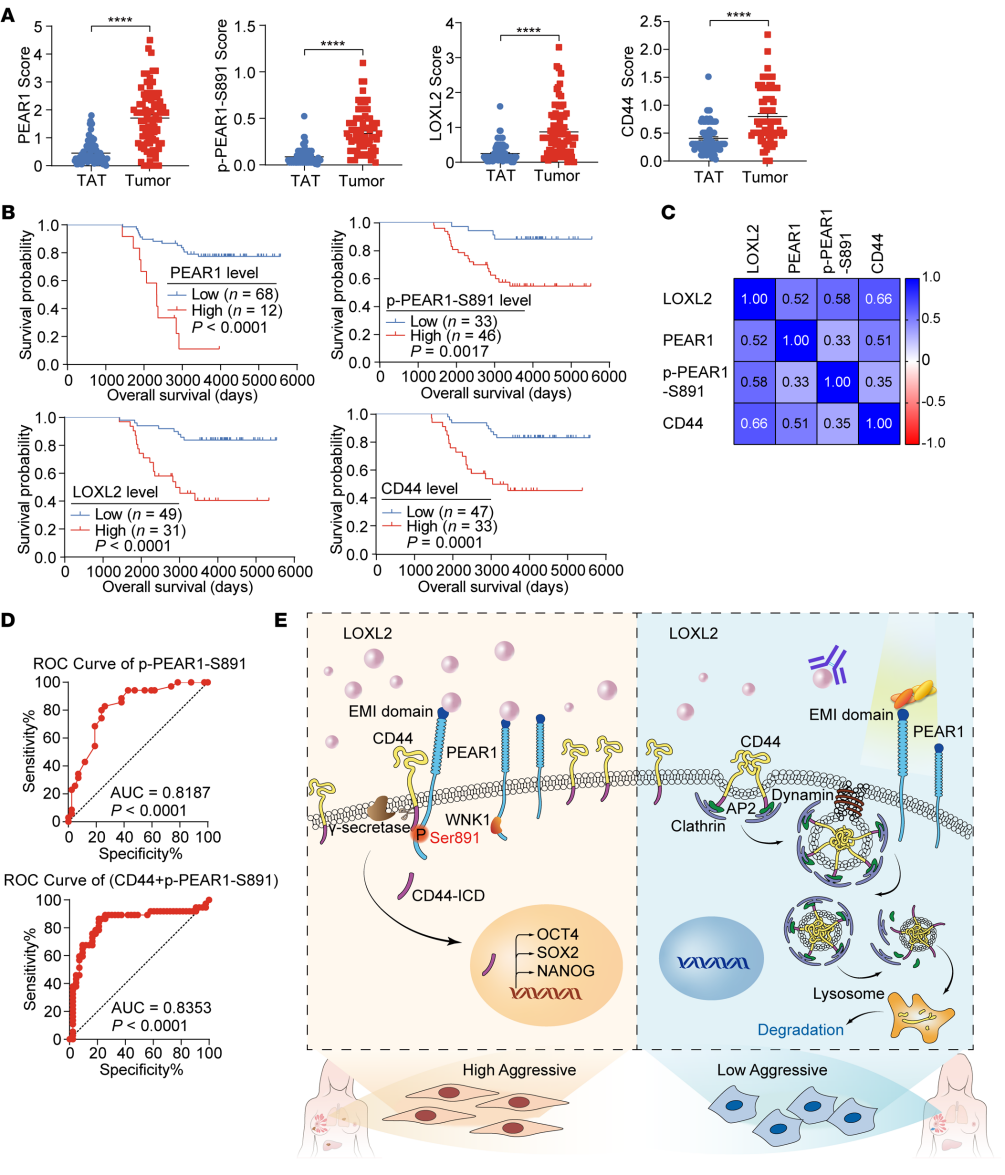
The LOXL2/PEAR1/CD44 axis is upregulated in TNBC and is associated with poor overall survival. (**A**) Quantification of PEAR1, phospho-PEAR1 Ser891, LOXL2, and CD44 staining scores by IHC in TNBC and corresponding adjacent samples (*n* = 80 for TAT and tumor samples; mean ± SEM). (**B**) Overall survival of patients with TNBC based on PEAR1, phospho-PEAR1 (Ser891), LOXL2, and CD44 expression levels (*n* values as indicated; log-rank test). (**C**) Heatmap of the correlation between the expression levels of PEAR1, phospho-PEAR1 Ser891, LOXL2, and CD44 in TNBC samples (*n* = 80; Pearson’s correlation analysis). (**D**) The prognostic effect of the risk score of phospho-PEAR1 Ser891 and its combination with CD44 for patients with TNBC (time-dependent ROC curve analysis). (**E**) Schematic diagram of the mechanisms by which the LOXL2/PEAR1/CD44 pathway regulates TNBC metastasis. Unpaired 2-tailed *t* tests were used for **A**; log-rank test was used for **B**; Pearson’s correlation analysis was used for **C**; time-dependent ROC curve analysis was used for **D**. ****P < 0.0001.

To investigate the relationships between LOXL2, PEAR1, and PEAR1 Ser891 phosphorylation and CD44 in breast cancer, we subjected human TNBC samples to IHC. There is no commercially available antibody that recognizes phosphorylated PEAR1 at Ser891. Therefore, our research group developed a polyclonal antibody, which showed great reactivity and specificity for Ser891-phosphorylated PEAR1 compared with nonphosphorylated PEAR1 (Supplemental Figure 9, A–D). IHC staining results showed that PEAR1, phosphorylated PEAR1 at Ser891, LOXL2, and CD44 expression levels were significantly increased in TNBC tissues compared with corresponding paracancerous tissues (Figure 7A and Supplemental Figure 9E). PEAR1, phosphorylated PEAR1 at Ser891, LOXL2, and CD44 expression levels in TNBC patients were negatively correlated with overall survival (Figure 7B). Furthermore, Pearson’s correlation analysis revealed a significant positive correlation among PEAR1, phosphorylated PEAR1 at Ser891, LOXL2, and CD44 expression levels (Figure 7C and Supplemental Figure 9F). Time-dependent receiver operating characteristic (ROC) curve analysis revealed that PEAR1 Ser891 phosphorylation was the best independent prognostic factor in patients with TNBC, and PEAR1 Ser891 phosphorylation combined with CD44 expression exhibited a stronger prognostic effect than PEAR1 Ser891 phosphorylation alone (Figure 7D and Supplemental Figure 9G). Overall, our results revealed the critical roles of LOXL2-induced PEAR1 Ser891 phosphorylation in protecting CD44 from endocytosis-mediated degradation and activating CD44-ICD signaling, which might serve as a promising target for TNBC therapy (Figure 7E).

## Discussion

CD44 is a well-known biomarker for CSCs and regulates metastasis and drug resistance in many cancers. CD44, a cell surface adhesion receptor, participates not only in mediation of cell adhesion but also in signaling transduction. CD44-ICD is essential for CD44-mediated signal transduction (27). However, the downstream signaling pathways of CD44-ICD are extremely complicated. On the one hand, although CD44-ICD itself lacks kinase activity, it can interact with cofactors and adaptor molecules to regulate actin-cytoskeleton network remodeling and various cellular signaling pathways, which subsequently enhance the invasive behavior of tumor cells (15). On the other hand, CD44-ICD is cleaved by presenilin-1/γ-secretase and then translocates to the nucleus due to the transportin 1–specific nuclear localization signal (residues DRKPS) (28, 29). CD44-ICD binds to several promoter response elements, thereby activating the gene expression of stemness factors (e.g., OCT4, SOX2, and NANOG), regulators of EMT (e.g., MMP9 and TWIST1), and CD44 itself (29–33). Here we found that CD44-ICD nuclear levels and TNBC cell invasion and metastasis were significantly suppressed by the γ-secretase inhibitor DAPT. These results suggested that cleaved CD44-ICD was the major mediator of CD44 signal transduction in TNBC metastasis. We also found that CD44-ICD cleavage was regulated by PEAR1 through the direct interaction between PEAR1-ICD and CD44-ICD. However, no interaction was observed between PEAR1 and subunits of γ-secretase (data not shown), suggesting that PEAR1 does not regulate γ-secretase activity directly. Given that serine phosphorylation of PEAR1-ICD is essential for CD44-ICD cleavage, we hypothesized that PEAR1-ICD regulates CD44-ICD cleavage by signal-driven CD44-ICD conformational changes and subsequent γ-secretase facilitation. However, the exact molecular mechanisms involved are unclear.

Endocytosis plays an important role in the regulation of membrane receptor–ligand signaling. Following endocytosis, receptors enter the endosystemic recirculatory complex (ERC) for recycling to the membrane or enter the lysosome for degradation (34). CD44 is endocytosed after binding to the ligand hyaluronan (35). In fact, CD44 antibody–coupled drugs are designed according to the principle of CD44 endocytosis. Here we found that the internalization of CD44 was dependent on a clathrin-mediated endocytosis pathway. Previous studies have suggested that certain sorting signals, such as ubiquitination, determine where the receptor is located (36, 37). However, CD44 was not ubiquitinated following PEAR1 knockdown or overexpression (data not shown), indicating that the fate of CD44 after endocytosis was independent of ubiquitination. We found that serine-phosphorylated PEAR1 acted as a chaperone protein for CD44 and protected it from lysosomal degradation. Interference with this interaction changed the trajectory of CD44 degradation following endocytosis, thus decreasing TNBC cell stemness and metastasis. However, the mechanism by which PEAR1 phosphorylation regulates CD44 endocytosis, whether through recycling to the membrane or degradation by lysosomes, needs to be further elucidated.

Despite the promising therapeutic potential of CD44 as a tumor target, the side effects of targeting CD44, such as severe skin toxicity, are the greatest obstacles to clinical treatment due to the wide and high-level expression of CD44 in normal tissues. PEAR1 is generally expressed at low levels in normal tissues, but PEAR1 levels are significantly elevated under pathological conditions (17, 38). These data suggest that PEAR1 may represent an important regulator of disease progression and may serve as a specific target for therapy. In addition, diverse CD44 isoforms are noted in different tumors. Several tumor cells, including MDA-MB-231 cells, mainly express CD44s (39, 40), whereas others express CD44v with an additional domain in the extracellular region close to the membrane. PEAR1 binds to the ICD of CD44, which is the same for both isoforms. Moreover, the results of online analysis with TIMER2.0 suggested a positive correlation between PEAR1 and CD44 expression levels in numerous cancers, such as blood, brain, colorectal, and ovarian cancer (data not shown). Consequently, we hypothesize that PEAR1 may serve as a more effective therapeutic target in various cancers.

LOXL2 is an extracellular enzyme that catalyzes the oxidative deamination of peptidyl lysine residues and promotes the lysyl-derived cross-linking of collagen and elastin in the ECM. Many studies have revealed that aberrant LOXL2 expression in multiple cancers is associated with tumor metastasis, poor prognosis, and chemoradiotherapy resistance (41, 42). Interestingly, we found that LOXL2 SRCR1-2 and SRCR1-3, in which the SRCR4 and lysyl oxidase–like domains had been deleted, had prometastatic effects similar to those of full-length LOXL2. These data indicated that the function of LOXL2 was independent of ECM remodeling, which was consistent with the phenotype of the *LOXL2*-knockout mouse model (43). Notably, in addition to affecting ECM remodeling, LOXL2 affects intracellular signaling independent of its catalytic activity (44, 45). However, the underlying mechanism has not yet been determined. In this study, we found that LOXL2, an extracellular ligand of PEAR1, induced PEAR1-ICD phosphorylation and its binding to CD44, and subsequently affected downstream invasion-related signaling. Blocking the interaction between LOXL2 and PEAR1 with a PEAR1-EMI monoclonal antibody significantly inhibited TNBC metastasis both in vitro and in vivo, indicating that PEAR1 is the major receptor mediating the nonenzymatic activity of LOXL2 in cancer metastasis and that the LOXL2/PEAR1 axis is a promising target for TNBC therapy.

At present, the development of LOXL2 inhibitors is focused on enzyme activity. Simtuzumab, a monoclonal antibody against LOXL2, acts as a noncompetitive inhibitor of LOXL2 through allosteric inhibition by binding to the SRCR4 domain, and this domain plays an important role in optimizing its catalytic activity (46). The binding domain of LOXL2 with PEAR1 is SRCR1-2, which is distant from the SRCR4 domain. We found that simtuzumab suppressed the interaction of LOXL2 with PEAR1 and TNBC cell invasion and migration only at high concentrations, suggesting that simtuzumab inhibits LOXL2 binding to PEAR1, probably through space occupation. Simtuzumab has shown limited efficacy in patients with pancreatic cancer or KRAS-mutant colorectal cancer (47, 48), which may be due to the indirect and weak blocking effect of simtuzumab on the interaction of LOXL2 with PEAR1. Based on the above findings, we propose that targeting LOXL2 for cancer treatment should compensate for the inhibition of its enzymatic functions and interaction with PEAR1.

Overall, this study revealed an innovative regulatory mechanism in which the interplay of the LOXL2/PEAR1/CD44 axis drives TNBC metastasis. In particular, LOXL2-induced PEAR1 phosphorylation at Ser891 activated CD44-ICD signaling and safeguarded CD44 against endocytosis-mediated degradation. Disrupting the interaction between LOXL2 and PEAR1 has been demonstrated to be a promising target for antimetastatic therapy both in vitro and in vivo. Importantly, PEAR1 phosphorylation at Ser891, which surpassed that of CD44 and LOXL2 as the better biomarker of TNBC, is considered a robust independent prognostic factor in TNBC patients, thus suggesting its potential as a therapeutic candidate for TNBC.

## Methods

### Sex as a biological variable.

This study exclusively examined female human breast tissue microarrays and female mice, because the disease modeled — breast cancer — is mainly relevant in females.

### Animals.

Six-week-old female BALB/c (stock jlc0003), BALB/c-*nu*/*nu* (jlc0005), and NOD-SCID (jlc0008) mice were purchased from Shanghai Lingchang Biotechnology Co. All mice were housed under specific pathogen–free conditions (12-hour light/12-hour dark cycle, 50% relative humidity, and 22°C ± 2°C) with free access to a normal laboratory diet (SZS9126, Xietong Pharmaceutical Bioengineering) and sterile water and were monitored by inspection twice each day.

### Cell lines and culture conditions.

The human breast cancer cell lines MDA-MB-231 (stock HTB-26), SUM159 (Y-XB-2391), MDA-MB-468 (HTB-132), and MCF7 (CRL-12584), the murine TNBC cell line 4T1 (CRL-2539), and the human cell lines HEK293T (CRL-3216) and HEK293FT (PTA-5077) were all purchased from ATCC. These cells were cultured in DMEM (L110KJ, BasalMedia) supplemented with 10% FBS) (F8318, Sigma-Aldrich) and 1% penicillin/streptomycin (C100C5, NCM Biotech) at 37°C in a humidified incubator with 5% CO_2_.

### Generation of the monoclonal antibody for human PEAR1.

Various anti–human PEAR1 monoclonal antibodies were developed by our research group by the hybridoma technique and screened by ELISA. After sequencing, the antibody was expressed in HEK293S cells and purified by protein A beads (17). For the therapeutic antibody, to avoid the possibility of PEAR1 clustering caused by the 2 Fab arms of the normal antibody and to prolong the lifespan of the antibody in vivo, 1 Fab of the antibody was coupled to HSA, and Fab-HSA targeting the PEAR1-EMI domain was obtained.

### Generation of the anti–phospho-PEAR1 Ser891 antibody.

An anti–phospho-PEAR1 Ser891 antibody (p-PEAR1 Ser891 Ab) was generated by Shanghai GL Biochem Co. Rabbit antiserum against PEAR1 Ser891 phosphorylation was generated using the peptide Cys-RGSSRLDRSY(pS)YSYSNGP coupled to the carrier protein keyhole limpet hemocyanin (KLH), the Ser891 of which is phosphorylated and indicated as pS. The antiserum was precleaned with affinity chromatography using the corresponding nonphosphorylated peptide (Cys-RGSSRLDRSYSYSYSNGP) coupled to Pierce NHS-Activated Agarose and purified by affinity chromatography. The nonphosphorylated PEAR1 antibody was purified using nonphosphorylated peptide and used as a basal control for the p-PEAR1 Ser891 antibody. The affinity and specificity of this anti–phospho-PEAR1 Ser891 antibody were evaluated as shown in Supplemental Figure 9, A–D.

### Plasmid construction and transfection.

To generate stable PEAR1-overexpressing and PEAR1-knockdown cells and stable CD44-knockdown cells, we used a lentiviral system. Specifically, PEAR1-overexpressing plasmids (WT, mutated-SA, and EMI deletion) and their corresponding negative controls were synthesized by Shanghai Saiheng Biotechnology Co. Lentiviral vectors encoding human PEAR1/murine Pear1–based shRNA (shPEAR1/shPear1) and human CD44–based short hairpin shRNA (shCD44), as well as control hairpins (shnc), were designed and synthesized by Shanghai Genechem Co. Lentivirus was produced by cotransfecting HEK293FT cells with the target plasmids alongside *psPAX2* and *pMD2.G*. After 48 and 72 hours, the lentiviral supernatants were collected, centrifuged at 2,000 *g* for 15 minutes, and filtered through 0.45 μm sterile filters (SLHVR33RB, Millex). Subsequently, breast cancer cells were infected with lentivirus overnight in the presence of 8 μg/mL polybrene (40804ES76, Yeasen) and selectively cultured with 2 μg/mL puromycin (60210ES60, Yeasen). The *PEAR1*-ICD–Flag cDNA sequence was synthesized and cloned and inserted into the pcDNA3.4 vector, and the *CD44*-ICD-HA cDNA sequence was synthesized and cloned and inserted into the pcDNA3.1+ vector. At approximately 70% confluence, cell transfections were performed using Lipofectamine 2000 (11668019, Thermo Fisher Scientific) according to the manufacturer’s instructions. Transfection efficiency was assayed after 48 hours using RT-qPCR and Western blotting. The sequences for the plasmids and shRNA are listed in Supplemental Table 1.

### Mouse experiments.

For the xenograft models, 6-week-old female BALB/c-*nu*/*nu* mice were randomly divided into 2 groups (*n* = 5 per group). Either negative control–transfected MDA-MB-231 cells or shPEAR1-transfected MDA-MB-231 cells (5 × 10^6^) suspended in 50 μL sterile PBS were orthotopically injected into the inguinal mammary fat pads of the mice. Similarly, 6-week-old female BALB/c mice were randomly assigned to 2 groups (*n* = 5 per group). Either negative control–transfected 4T1 cells or shPear1-transfected 4T1 cells (2 × 10^6^), diluted in 50 μL sterile PBS, were orthotopically injected into the inguinal mammary fat pads of the mice. Tumor volumes were assessed using caliper measurements and calculated using the following formula: *V* = *a* × *b*^2^/2 (*a*, longer diameter; *b*, shorter diameter). For the metastasis models, 6-week-old female BALB/c-*nu*/*nu* mice were randomly divided into 2 groups (*n* = 5 per group). Either negative control–transfected MDA-MB-231 cells or shPEAR1-transfected MDA-MB-231 cells (5 × 10^5^) were diluted in 100 μL sterile PBS and injected via the tail vein. The same procedures were employed for oe-PEAR1-WT–transfected MDA-MB-231 cells and oe-PEAR1–mutated–all SA/S891A-transfected MDA-MB-231 cells. For PEAR1 Fab-HSA treatment, 6-week-old female NOD-SCID mice were divided into 3 groups (*n* = 5 per group). The mice were injected with MDA-MB-231 cells (5 × 10^5^), diluted in 100 μL sterile PBS, and then treated concurrently with i.v. administration of PEAR1 Fab-HSA (3.35 mg/kg), control HSA (3.35 mg/kg), or vehicle (PBS) via the tail vein. This treatment was continued every 4 days for 40 days until the mice were sacrificed. At the end of the experiments, mice were euthanized, and the tumors, lungs, and livers were resected for analysis.

### Tissue microarray analysis.

Human breast tissue microarrays containing 126 breast tumor samples (stock HBre-Duc090Sur-01), 86 normal adjacent breast samples (HBreD145Su01), and 80 corresponding paracancer tissue samples (TNBC-1602) were acquired from Shanghai Outdo Biotech Co. IHC staining of the tissues for PEAR1 (HPA035217, Sigma-Aldrich), phospho-PEAR1 Ser891 (developed as described above), LOXL2 (67139-1-Ig, Proteintech), and CD44 (3570, Cell Signaling Technology) was performed. Subsequently, the stained tissue microarrays were scanned with an Aperio ScanScope and analyzed with Aperio ImageScope software version 12.3.2. The results were scored according to the intensity and area of the cellular staining. Staining intensity was scored as 0 (negative particles), 1 (faintly yellow particles), 2 (brownish-yellow particles), and 3 (brown particles). The final score was determined by multiplying the staining color scores by the proportion of positively stained area. Survival data were evaluated with Kaplan-Meier curves and subjected to statistical analyses through log-rank tests. Correlations between the parameters were determined using Pearson’s correlation analysis. The prognostic effects of the risk scores were assessed using the AUC in time-dependent ROC curve analysis using 10 years as the judgment period. The risk score was the product of the IHC staining score and the hazard ratio [HR = risk function *h1*(*t*) in the exposed group/risk function *h2*(*t*) in the nonexposed group, where *t* refers to the same point in time]. The clinical and pathological characteristics of the patients’ tumors are presented in Supplemental Tables 2 and 3.

### H&E staining.

Tissues were fixed for 36 hours in 4% PFA (E672002-0500, Sangon Biotech), embedded in paraffin, and sectioned at a thickness of 5 μm. The paraffin-embedded tissue sections were deparaffinized and rehydrated, incubated with hematoxylin (BA4027, BASO) for 5 minutes, incubated with eosin (BA4027, BASO) for 1 minute, and then thoroughly washed with ddH_2_O between each step. After dehydration, the slides were sealed with neutral balsam (36313ES60, Yeasen). Histologic characteristics were described and metastatic nodules were enumerated under a light microscope.

### RNA isolation and RT-qPCR.

Total RNA extraction from cells was performed using RNA Isolater Total RNA Extraction Reagent (R401-01, Vazyme), following the manufacturer’s instructions. cDNA was synthesized from 1 μg total RNA using PrimeScript RT Master Mix (Perfect Real Time, RR036A, Takara) and amplified with TB Green Premix Ex Taq II (Tli RNaseH Plus, RR820A, Takara) using the LightCycler 96 Real-Time PCR System. Relative expression of mRNA was determined after normalization to *18S* rRNA and calculated using the 2^−ΔΔCT^ method. For a detailed list of primer sequences, see Supplemental Table 4.

### Western blotting.

Cultured cells were collected with a scraper and then lysed with RIPA buffer (P0013B, Beyotime; composed of 50 mM Tris-HCl [pH 7.4], 150 mM NaCl, 1% Triton X-100, 1% sodium deoxycholate, 0.1% SDS with phosphatase and protease inhibitors) on ice for 15 minutes, and the samples were sonicated, followed by centrifugation at 12,000 *g* for 20 minutes. Nuclear and cytoplasmic protein extraction (P0028, Beyotime) and membrane and cytosol protein extraction (P0033, Beyotime) were performed following the corresponding kit protocols. The resulting cell lysates were collected and quantified with a BCA Protein Assay Kit (20201ES90, Yeasen). Following denaturation at 100°C for 10 minutes, equivalent amounts of protein samples were loaded and separated using 8%–12% SDS-PAGE and then transferred onto 0.45 μm PVDF membranes (IPVH00010, Millipore). The immunoblots were incubated in blocking buffer (consisting of 5% [wt/vol] skim milk in TBST, 10 mM Tris-HCl [pH 7.5], 500 mM NaCl, and 0.1% Tween 20) for 2 hours at room temperature, followed by incubation with specific antibodies overnight at 4°C. Then the immunoblots were washed 3 times for 10 minutes in TBST, incubated with HRP-conjugated secondary antibodies diluted in blocking buffer for 1 hour at room temperature, and washed 3 times in TBST again. The detected signals were visualized with enhanced chemiluminescence (P10300, NCM Biotech) using the Tanon 2500 Luminescence imaging system.

### co-IP.

The transfected cells were lysed with cell lysis buffer (P0013, Beyotime; composed of 20 mM Tris-HCl [pH 7.5], 150 mM NaCl, 1% Triton X-100, 1% sodium with phosphatase and protease inhibitors) on ice for 15 minutes, followed by centrifugation at 12,000 *g* for 20 minutes. For endogenous co-IP, the lysates were incubated with the anti-PEAR1 antibody (developed as described above) or anti-CD44 antibody (15675-1-AP, Proteintech) under gentle rotation overnight at 4°C, while corresponding isotype IgG (5415&2729, Cell Signaling Technology) was used as a negative control. The next day, protein A/G plus-agarose beads (sc-2003, Santa Cruz Biotechnology) were added to the lysates and incubated for 4 hours. For exogenous co-IP, the lysates were incubated with anti-Flag agarose beads (A2220, Sigma-Aldrich) or anti-HA agarose beads (26182, Thermo Fisher Scientific ) overnight under gentle rotation at 4°C. Afterward, the beads coupled to the immunocomplexes were collected and washed 3 times with TBS (composed of 10 mM Tris-HCl [pH 7.5] and 500 mM NaCl) by centrifugation at 12,000 *g* for 1 minute. Thereafter, the eluted proteins were subjected to denaturation at 100°C for 10 minutes and analyzed by MS (performed by the Public Platform of Basic Medicine, Shanghai Jiao Tong University School of Medicine) and Western blotting to detect the interacting proteins.

### Pulldown assay.

MDA-MB-231 cells were cultivated with the PEAR1 ligands until they reached approximately 85% confluence in a 10 cm culture dish. Subsequently, the cells were gently washed 3 times with serum-free medium and then cultured in serum-free medium at 37°C with 5% CO_2_ for 24 hours. Afterward, the supernatants were collected, centrifuged at 2,000 *g* for 15 minutes, and then filtered through 0.22 μm filters (SLGVR33RB, Millex). The resulting filtrate was then concentrated using Amicon Ultra-4 (3 kDa MWCO; UFC8003, Millipore). The secreted proteins in supernatant were incubated with PEAR1-ECD–His protein (17), and Dynabeads His-tag (10103D, Thermo Fisher Scientific) were used to pull down the His-tagged protein and its interacting proteins per the manufacturer’s instructions. The eluate was subjected to silver staining. Then the differential bands were cut for further MS analysis and Western blotting. Analogously, the combination between PEAR1-ECD-His (17) and CD44-ECD–Fc (221334, Abcam) was examined using His-tag pulldown with Dynabeads.

### Confocal IF.

Cells were seeded onto microscopic glass coverslips (VWRI631-0149, VWR) in 24-well culture plates and cultured overnight. Following drug administration, the cells were gently rinsed with PBS and fixed with 4% PFA for 1 hour. Then the cells were rinsed twice with PBS, followed by permeabilization with 0.2% Triton X-100 (93443, Sigma-Aldrich) in PBS for 10 minutes at room temperature. Thereafter, the cells were blocked in 2% BSA (B2064, Sigma-Aldrich) in PBS for 1 hour at room temperature. Then the cells were incubated with primary antibodies in PBS containing 1% BSA overnight at 4°C. The next day, the cells were rinsed twice with PBS and then incubated with fluorochrome-conjugated secondary antibodies for 2 hours at room temperature in the dark. Next, the cells were rinsed and sealed with DAPI Fluoromount-G (36308ES20, Yeasen). Finally, imaging was performed under a Leica TCS Sp8 STED confocal microscope with a 100× magnification objective.

### Mammosphere formation.

Cells were trypsinized (C100C1, NCM Biotech) and cultured in 24-well Nunclon Sphera plates (174930, Thermo Fisher Scientific), with a precise seeding of 1,000 cells per well in serum-free conditional medium (consisting of DMEM/nutrient mixture F-12 [DMEM/F12; L310KJ, BasalMedia] supplemented with 20 ng/mL EGF [CYT-217, Prospec], 20 ng/mL basic FGF [bFGF; CYT-288, Prospec], 4 μg/mL insulin [40107ES25, Yeasen], 0.4 ng/mL hydrocortisone [40109ES08, Yeasen], 0.4% BSA, 1× B27 [12587010, Gibco], and 1% penicillin/streptomycin) at 37°C in a humidified incubator with 5% CO_2_. The mammospheres were formed for the first time after approximately 10 days. Then the mammospheres were trypsinized again, prepared into a single-cell suspension with serum-free medium, and cultured under ultra-low adhesion condition. After 20–25 days, mammospheres were imaged and counted under a microscope.

### Transwell assay.

Matrigel (BD 356234; Matrigel/DMEM 1:3, serum-free) was added to the upper chamber of an 8-μm-pore-size insert (CLS3422, Corning) and allowed to gel at 37°C for 3 hours. Then the cells were suspended and seeded with serum-free medium containing diverse treatments into the upper chamber (1 × 10^5^ cells per well), whereas the lower 24-well plates were filled with 750 μL DMEM containing 20% FBS to support cell health. Following 48-hour incubation at 37°C, the noninvasive cells were removed by wiping with cotton swabs, whereas the cells that adhered to the underside of the chamber were fixed with methanol and stained with 0.1% crystal violet (C8470, Solarbio) for 30 minutes. The number of invading cells was calculated using microscope images taken from 5 random fields.

### Wound healing assay.

Cells were seeded into 6-well plates (2 × 10^6^ cells per well) and cultured to full confluence in complete DMEM. Subsequently, media were replaced by serum-free media for 24 hours, and a sterile 200 μL pipette tip was used to create “wounds’” by gently scratching the confluent cell monolayers. Samples were gently washed with PBS to remove cell debris. The cultured cells were then cultured in serum-free media supplemented with various drugs. Images of the wounds were captured using a microscope at 0, 12, 24, 36, and 48 hours, and the healing rates were analyzed with ImageJ software (NIH).

### Cell counting kit-8 assay.

Cell proliferation was determined by employing the Cell Counting Kit-8 (CCK-8) assay. Following diverse treatments, cells were seeded into 96-well plates (5,000 cells per well) and incubated with 10% CCK-8 reagent (CK04, Dojindo) for 2 hours. Subsequently, cell viability was measured at 6 distinct time points: 0, 24, 48, 72, 96, and 120 hours. OD was detected at 450 nm by a microplate reader.

### EdU cell proliferation assay.

The method was based on the incorporation of the thymidine analog EdU (5-ethynyl-2′-deoxyuridine) during DNA synthesis and the subsequent click reaction to label EdU with biotin. Then HRP-labeled streptavidin was added to biotin. To assess TMB (3, 3’, 5, 5’-Tetramethylbenzidine) color, we directly measured absorbance at 370 nm or 620–650 nm. Alternatively 2 M H_2_SO_4_ was added to terminate the reaction, and absorbance was subsequently measured at 450 nm. The kit instructions were followed to achieve a simple, rapid, and highly sensitive method for quantitative detection of cell proliferation in porous plates (C0088S, Beyotime Biotechnology).

### ELISA.

Briefly, 96-well EIA/RIA (enzyme immunoassay/radio immunoassay) plates (CLS3361, Corning) were coated with recombinant streptavidin (10 μg/mL, 100 μL; P5084, Beyotime) in coating buffer (15 mM Na_2_CO_3_, 35 mM NaHCO_3_, pH 9.6) for 1.5 hours at 37°C. Then the wells were washed 3 times with washing buffer (PBST, 0.05% Tween 20 in PBS), blocked with 2% BSA for 1 hour at 37°C, and coated with capture biotin-conjugated PEAR1-ECD domain peptides (EMI and EGF-like 1 through EGF-like 15; 10 μg/mL, 100 μL) in PBS overnight at 4°C. In contrast, the capture proteins were diluted in coating buffer directly and coated overnight at 4°C. The next day, the wells were washed 3 times with PBST, blocked with 2% BSA for 1 hour at 37°C, and washed 3 times with PBST again. Then 100 μL samples were added to the wells and incubated for 1 hour at 37°C, after which the plates were washed 4 times with PBST. Next, HRP-conjugated secondary antibodies (1:10,000) in 1% BSA in PBS were added to the wells for 30 minutes at 37°C, and the wells were washed 6 times with PBST before the addition of TMB (34029, Thermo Fisher Scientific). Ten minutes later, 2 M H_2_SO_4_ was added to the wells to stop the reaction, and OD was immediately measured at 450 nm using a microplate reader. The sequences of the recombinant proteins and peptides are listed in Supplemental Table 5.

### Quantification and statistical analyses.

All experiments were repeated 3 times independently, with similar results obtained, and representative data are shown in the figures. Statistical analyses were performed using GraphPad Prism 7.0 software or IBM SPSS Statistics 20.0 software. Kyoto Encyclopedia of Genes and Genomes (KEGG) pathway enrichment analyses of the MS data (Figure 1B) were performed using DAVID and KOBAS online. Differences between 2 groups were assessed by employing an unpaired 2-tailed Student’s *t* test. For comparisons, 1-way ANOVA followed by Dunnett’s post hoc test was used. To compare overall survival curves, Kaplan-Meier plots and the log-rank test were used. Correlations between parameters were assessed using Pearson’s correlation analysis. Prognostic effects of the risk scores were assessed by the AUC in a time-dependent ROC curve analysis. Details regarding data presentation and statistical analyses are provided within the figure legends. A 2-sided *P* value less than 0.05 was considered statistically significant.

### Study approval.

This study was approved by the Shanghai Jiao Tong University School of Medicine Science and Technology Ethics Committee. All experimental procedures were conducted in accordance with protocols approved by the Shanghai Jiao Tong University School of Medicine IACUC. All human tissue specimens were collected by Shanghai Outdo Biotech Co. in compliance with their informed consent policy. All animal studies were conducted in full accordance with the guidelines for the care and use of laboratory animals and were approved by the Shanghai Jiao Tong University School of Medicine IACUC.

### Data availability.

Values for all data points in graphs are reported in the Supporting Data Values file. The MS proteomics data have been deposited to the ProteomeXchange Consortium with the data set identifier PXD052128. Data can also be obtained from the corresponding author upon reasonable request.

## Author contributions

YS, XF, and JL designed and directed the project. YS performed all experiments and analyzed the data. JY, LL, HS, LZ, GL, XW, RL, XW, BH, and XS helped with the experiments. YS, XF, JL, and XS wrote the manuscript.

## Supplementary Material

Supplemental data

Unedited blot and gel images

Supporting data values

## Version 1 08/15/2024

Electronic publication
