# Supplementary material for: LOXL2-induced PEAR1 Ser891 phosphorylation suppresses CD44 degradation and promotes triple-negative breast cancer metastasis
Source: J Clin Invest. 2024 Aug 15;134(16):e177357. doi: 10.1172/JCI177357 (PMC11324313; doi:10.1172/JCI177357)
Supplement: Supplemental data [file jci-134-177357-s183.pdf]

Supplemental Figures

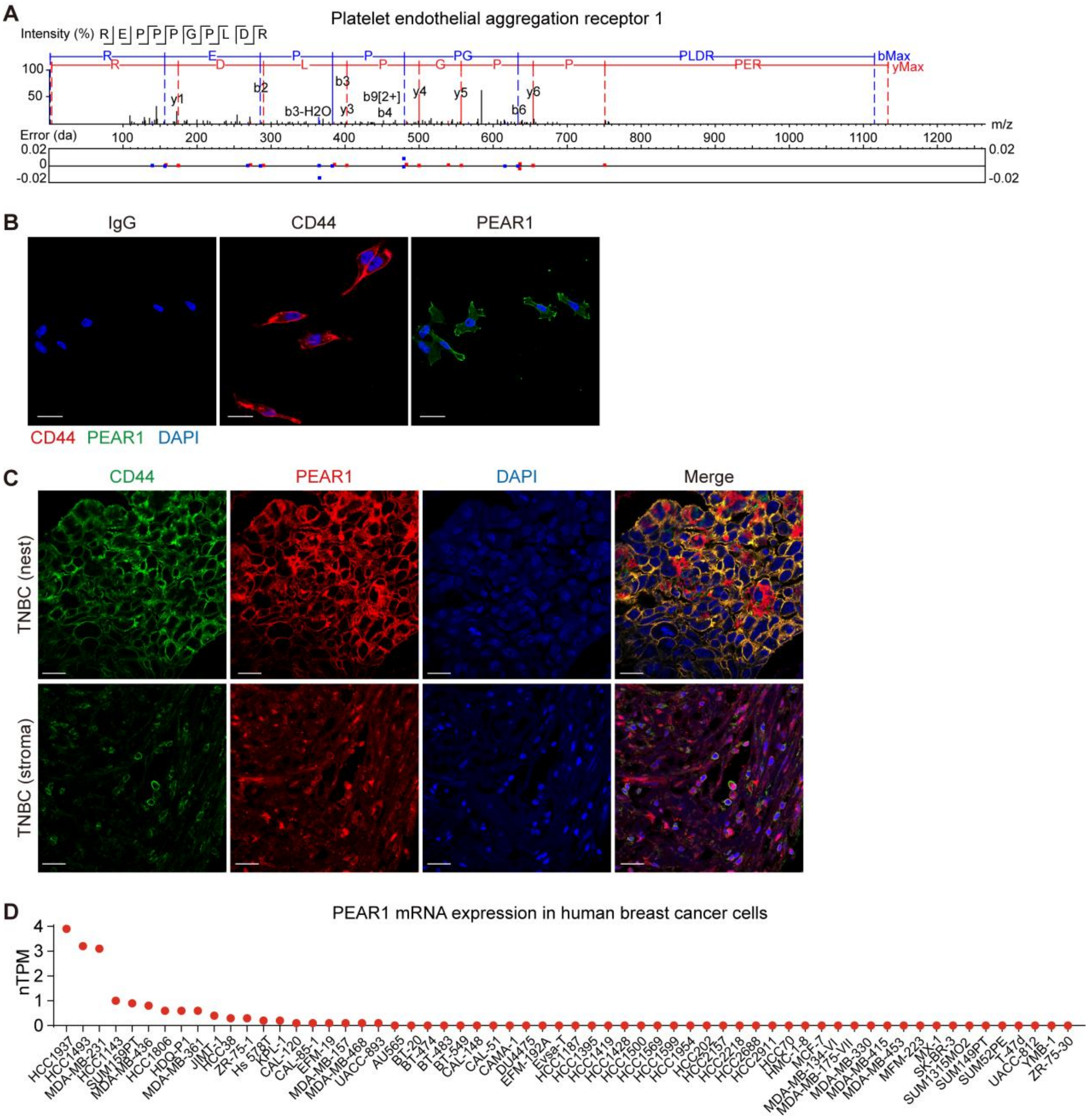

Supplemental Figure 1. PEAR1 is a CD44-associated protein that is correlated with poor survival in TNBC patients.

(**A**) MS analysis of PEAR1-specific peptide (REPPPGPLDR) from co-IP samples (CD44 and its interacting proteins) of whole-cell lysates of MDA-MB-231 cells using an anti-CD44 antibody. The IgG isotype control served as a negative control.

(**B**) Representative IF staining of MDA-MB-231 cells with anti-CD44 (red) and anti-PEAR1 (green) antibodies and nuclei (blue). Scale bar: 10 μm.

(**C**) Representative IF staining of TNBC tissue with anti-CD44 (green) and anti-PEAR1 (red) antibodies and nuclei (blue). Scale bar: 25 μm.

(**D**) *PEAR1* mRNA expression levels in various breast cancer cell lines from the Human Protein Atlas online.

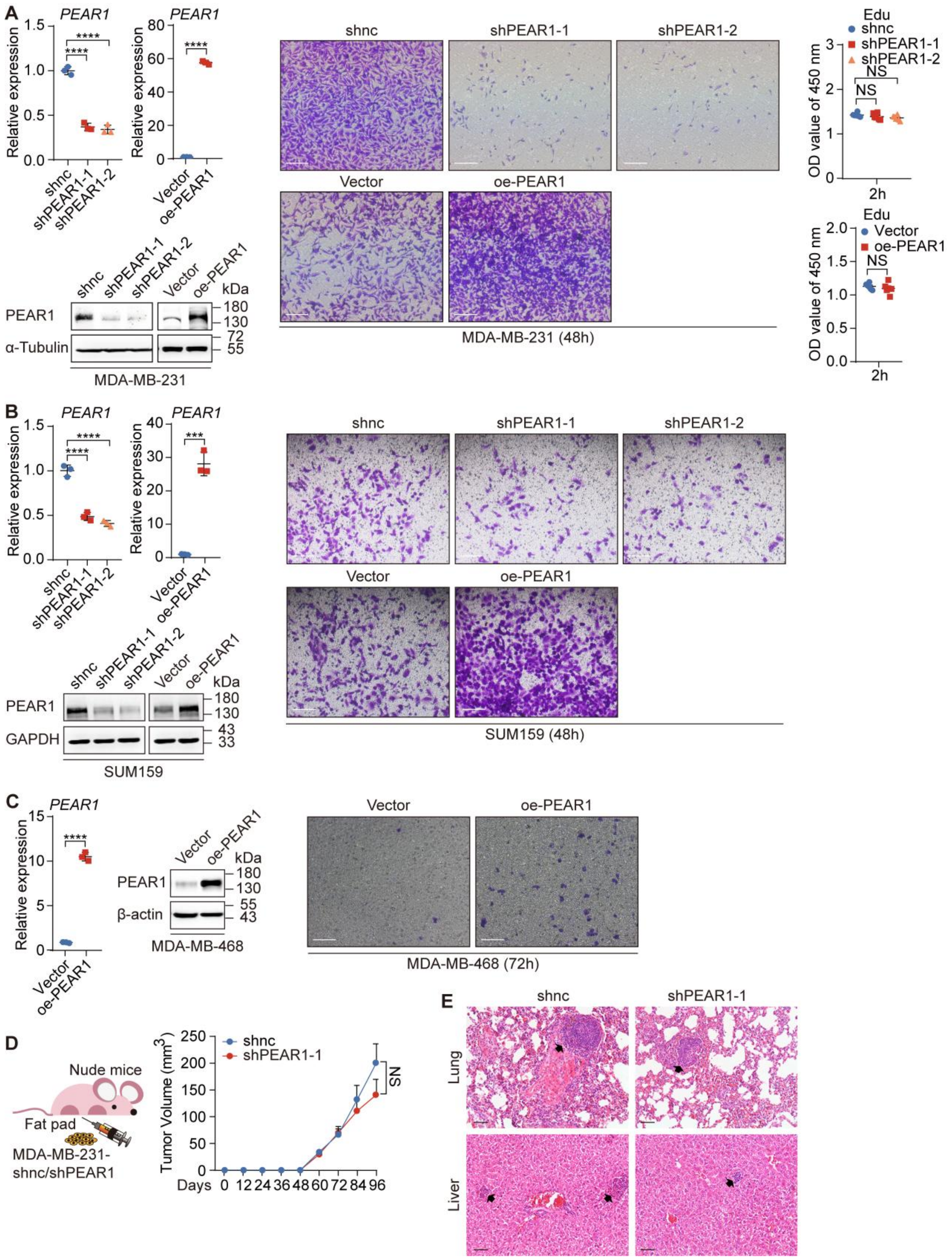

Supplemental Figure 2. PEAR1 exacerbates human TNBC cell metastasis.
(**A-C**) Stable shPEAR1 and oe-PEAR1 MDA-MB-231 and SUM159 cells, and oe-PEAR1 MDA-MB-468 cells were generated using lentivirus infection. Nontargeting shRNA was used as a negative control for shPEAR1 (shnc). The corresponding empty vector (Vector) was used as a negative control for oe-PEAR1. The expression levels of PEAR1 were confirmed with RT-qPCR and western blotting. The bar diagram shows the relative expression of mRNAs normalized to that of *18S rRNA* for qPCR (n = 3; mean ± SD). α-Tubulin, GAPDH, or β-actin was used as an

internal control for WB. Representative images of invasive shPEAR1- and oe-PEAR1-treated MDA-MB-231 and SUM159 cells, and oe-PEAR1-treated MDA-MB-468 cells in the transwell assay. Scale bar: 100 μm. Quantification of the proliferation levels of shPEAR1- and oe-PEAR1-treated MDA-MB-231 cells in the BeyoClick™ EdU assay (with TMB) (*n* = 3; mean ± SEM).

(**D**) ShPEAR1 and shnc MDA-MB-231 cells were injected into the orthotopic fat pads of nude mice and observed for 96 days, after which the growth curves of the tumors *in situ* were recorded (*n* = 5 mice per group; mean ± SEM).

(**E**) Representative images of metastatic foci in the lungs and livers of nude mice intravenously injected with shPEAR1 and shnc MDA-MB-231 cells by H&E staining (*n* = 5 mice per group). Scale bar: 50 μm.

Unpaired two-tailed t tests were used in (**A**), (**B**), (**C**), and (**D**); one-way ANOVA followed by Dunnett's test was used in (**A**) and (**B**). ***$P < 0.001$, ****$P < 0.0001$.

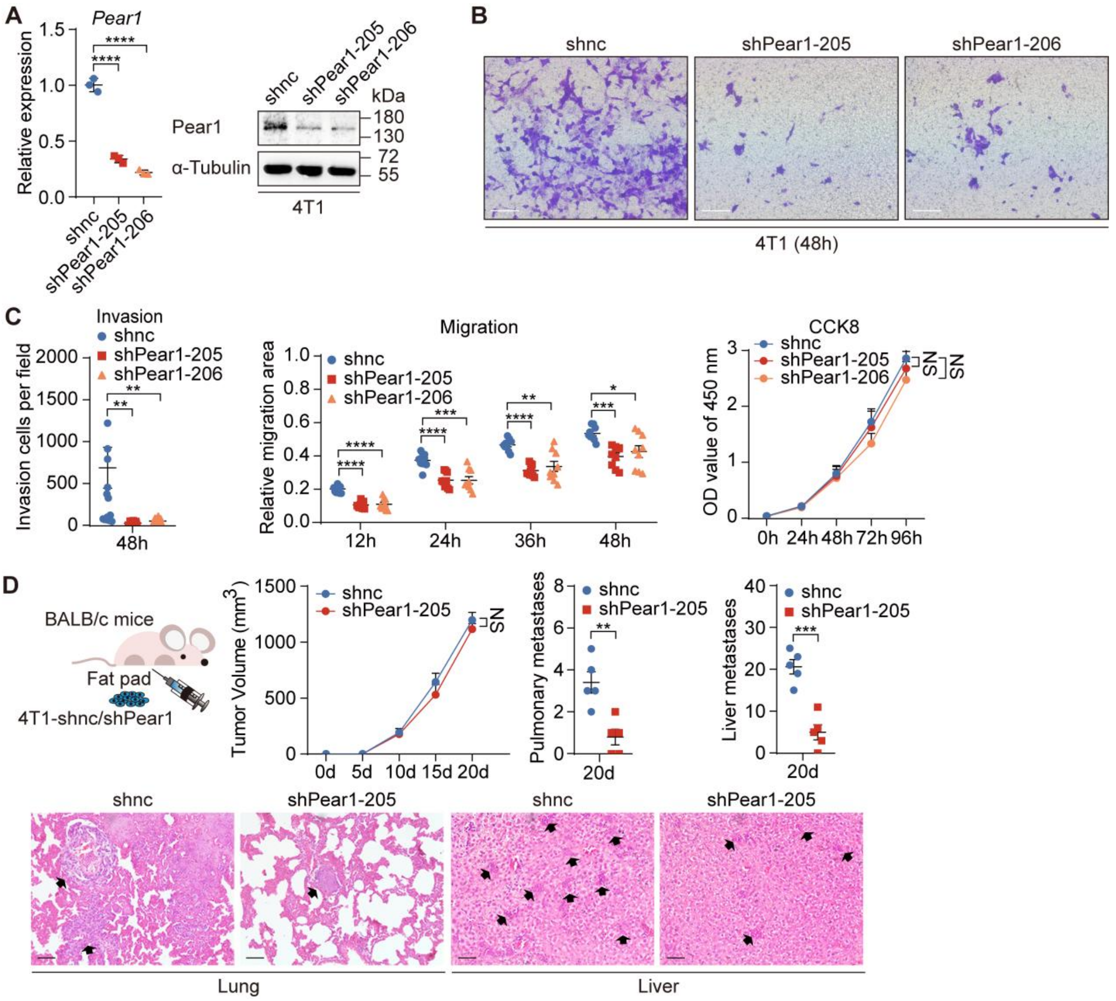

Supplemental Figure 3. PEAR1 exacerbates murine TNBC cell metastasis.

(**A**) Stable shPear1 4T1 cells were generated using lentivirus infection and confirmed with RT-qPCR and western blotting. The bar diagram shows the relative expression of mRNAs normalized to that of *18S rRNA* for qPCR (*n* = 3; mean ± SD). α-Tubulin was used as an internal control for WB.

(**B**) Representative images of invasive shPear1-treated 4T1 cells in a transwell assay. Scale bar: 100 μm.

(**C**) Quantification of invasive shPear1-treated 4T1 cells in a transwell assay, and quantification of the relative migration area of shPear1-treated 4T1 cells in the wound healing assay, and the viability of shPear1-treated 4T1 cells was assessed with a CCK8 assay kit (*n* = 3; mean ± SEM).

(**D**) ShPear1 and shnc 4T1 cells were injected into the orthotopic fat pads of BALB/c mice and observed for 20 days, after which the growth curves of the tumors *in situ* were recorded (*n* = 5 mice per group; mean ± SEM). Representative images and quantification of metastatic foci in the lungs and livers by H&E staining. Scale bar: 50 μm.

Unpaired two-tailed t tests were used in (**D**); one-way ANOVA followed by Dunnett's test was used in (**A**) and (**C**). *$P < 0.05$, **$P < 0.01$, ***$P < 0.001$, ****$P < 0.0001$.

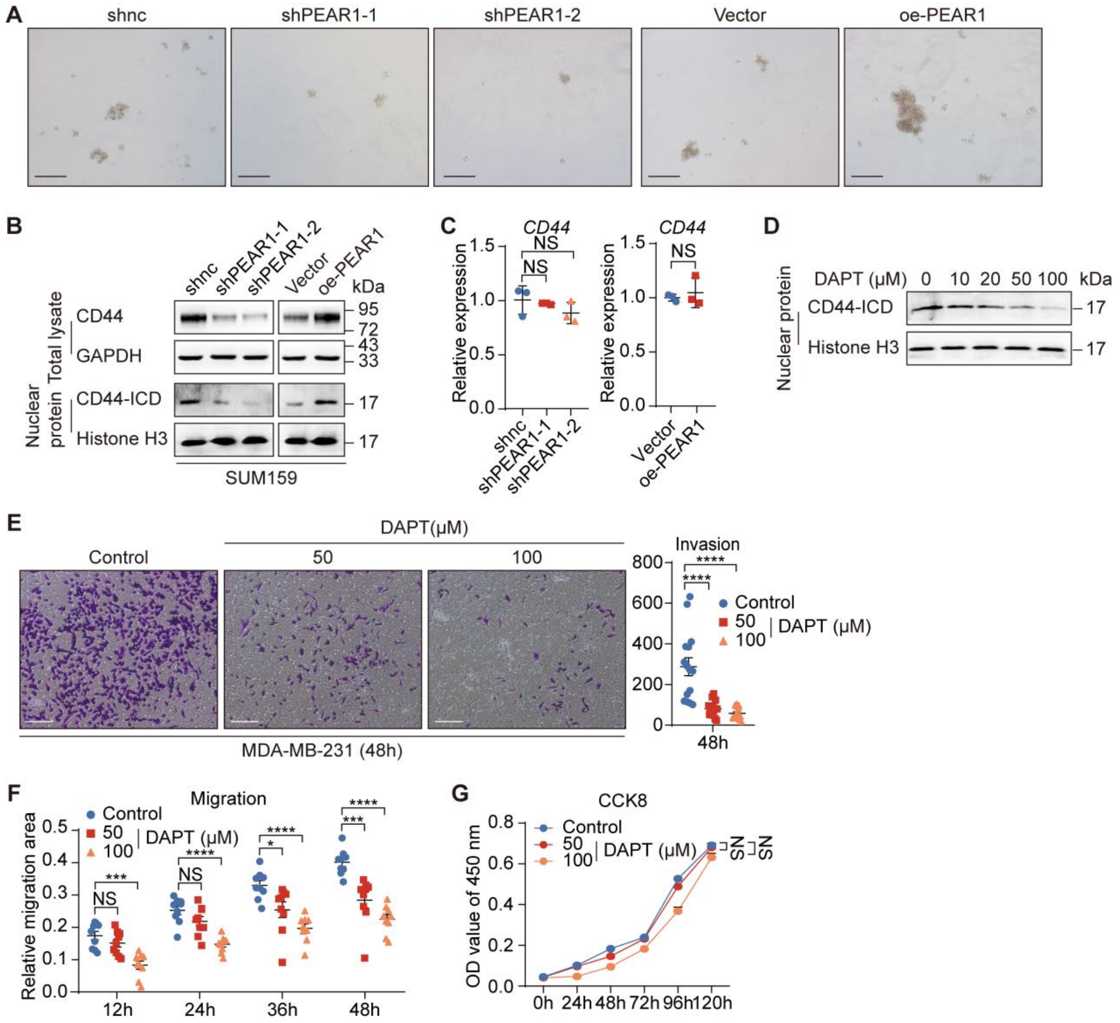

Supplemental Figure 4. CD44-ICD is an important messenger of CD44-mediated intracellular signals in MDA-MB-231 cells.

(**A**) Representative images of shPEAR1 and oe-PEAR1-treated MDA-MB-231 cells in the mammosphere formation assay. Scale bar: 50 µm.

(**B**) The expression levels of CD44 in total lysates and of CD44-ICD in nuclear proteins of shPEAR1 and oe-PEAR1 SUM159 cells determined by western blotting analysis. GAPDH was used as an internal control for total lysates; histone H3 was used as an internal control for nuclear proteins.

(**C**) *CD44* mRNA expression levels in shPEAR1 and oe-PEAR1 SUM159 cells were detected by RT-qPCR. The bar diagram shows the relative expression of mRNAs normalized to that of *18S rRNA* (n = 3; mean ± SD).

(**D**) The expression levels of CD44-ICD in nuclear proteins of MDA-MB-231 cells treated with 0, 10, 20, 50, or 100 µM DAPT (an inhibitor of γ-secretase) for 24 h. Histone H3 was used as an internal control for nuclear proteins.

(**E**) Representative images and quantification of invasive MDA-MB-231 cells treated with 0, 50, or 100 µM DAPT in a transwell assay (n = 3; mean ± SEM). Scale bar: 100 µm.

(**F**) Quantification of the relative migration area of MDA-MB-231 cells treated with 0, 50, or 100 µM DAPT in the wound healing assay (n = 3; mean ± SEM).

(**G**) The viability curves of MDA-MB-231 cells treated with 0, 50, or 100 μM DAPT were generated with a CCK8 assay kit ($n = 3$; mean ± SEM).

One-way ANOVA followed by Dunnett's test was used in (**C**), (**E**), (**F**), and (**G**); unpaired two-tailed t tests were used in (**C**). *$P < 0.05$, ***$P < 0.001$, ****$P < 0.0001$. The western blotting results were representative of three independent experiments.

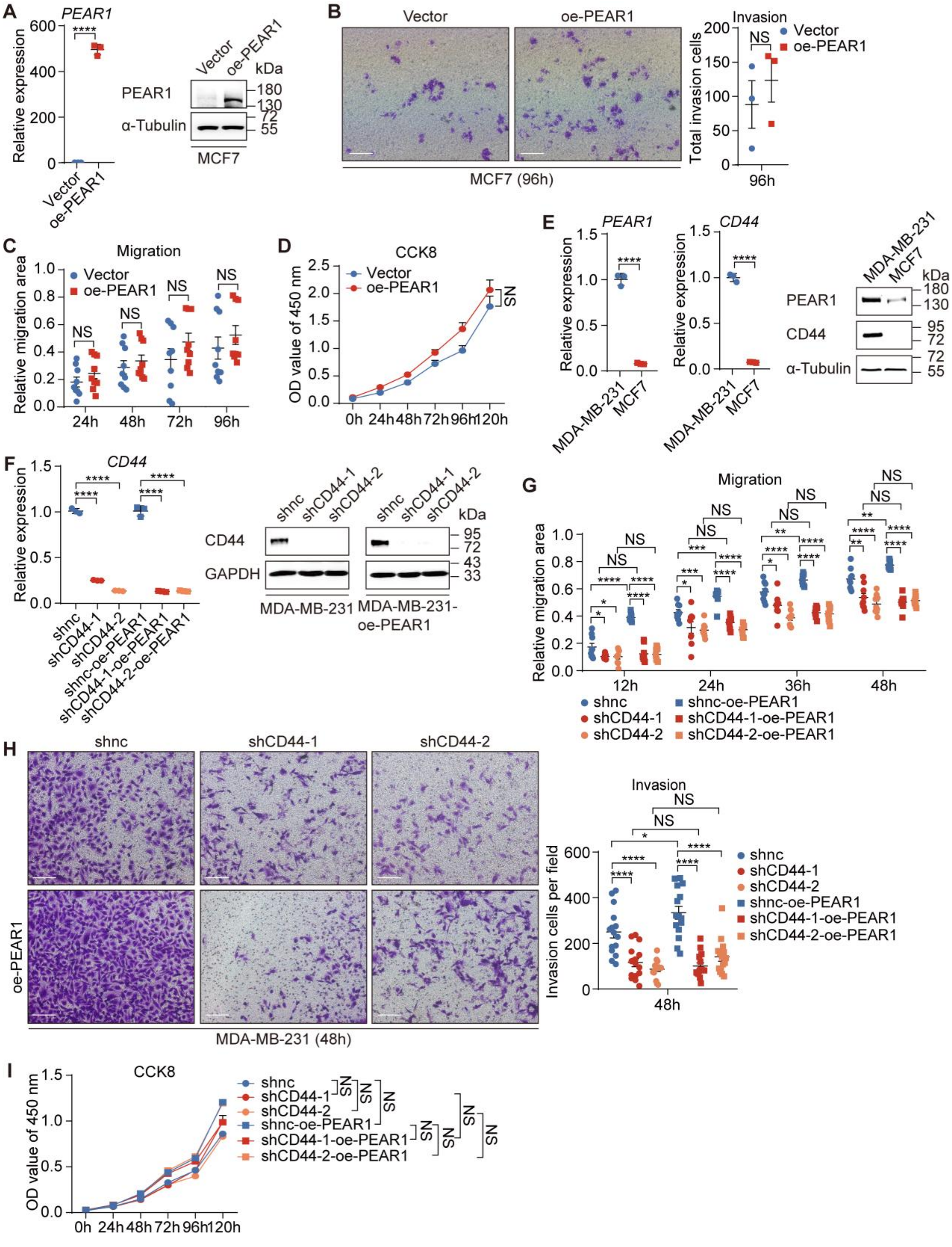

Supplemental Figure 5. PEAR1 regulates TNBC cell metastasis through CD44.

(**A**) Stable oe-PEAR1 MCF7 cells were generated using lentivirus infection, and confirmed with RT-qPCR and western blotting. The bar diagram shows the relative expression of mRNAs normalized to that of *18S rRNA* for qPCR (*n* = 3; mean ± SD). α-Tubulin was used as an internal control for WB.

(**B-D**) Representative images and quantification of invasive cells in transwell assay, the relative migration area in wound healing assay, and the viability in a CCK8 assay kit of oe-PEAR1-treated MCF7 cells (*n* = 3; mean ± SEM). Scale bar: 100 μm.

(**E**) The expression levels of PEAR1 and CD44 in MD-MB-231 cells and MCF7 cells

were detected by RT-qPCR and western blotting. The bar diagram shows the relative expression of mRNAs normalized to that of *18S rRNA* for qPCR (n = 3; mean ± SD). α-Tubulin was used as an internal control for WB.

(**F**) Stable CD44 knockdown (shCD44) and wide-type (WT) MDA-MB-231 cells, and stable shCD44 and oe-PEAR1 MDA-MB-231 cells were generated using lentivirus infection, and confirmed with RT-qPCR and western blotting. The bar diagram shows the relative expression of mRNAs normalized to that of *18S rRNA* for qPCR (n = 3; mean ± SD). GAPDH was used as an internal control for WB.

(**G-I**) The relative migration area in wound healing assay, representative images and quantification of invasive cells in transwell assay, and the viability in a CCK8 assay kit of shCD44-treated MDA-MB-231 cells (WT/oe-PEAR1-treated) cells (n = 3; mean ± SEM). Scale bar: 100 μm.

One-way ANOVA followed by Dunnett's test was used in (**F**), (**G**), (**H**), and (**I**); unpaired two-tailed t tests were used in (**A**), (**B**), (**C**), (**D**), (**E**), (**G**), (**H**), and (**I**). *$P < 0.05$, **$P < 0.01$, ***$P < 0.001$, ****$P < 0.0001$.

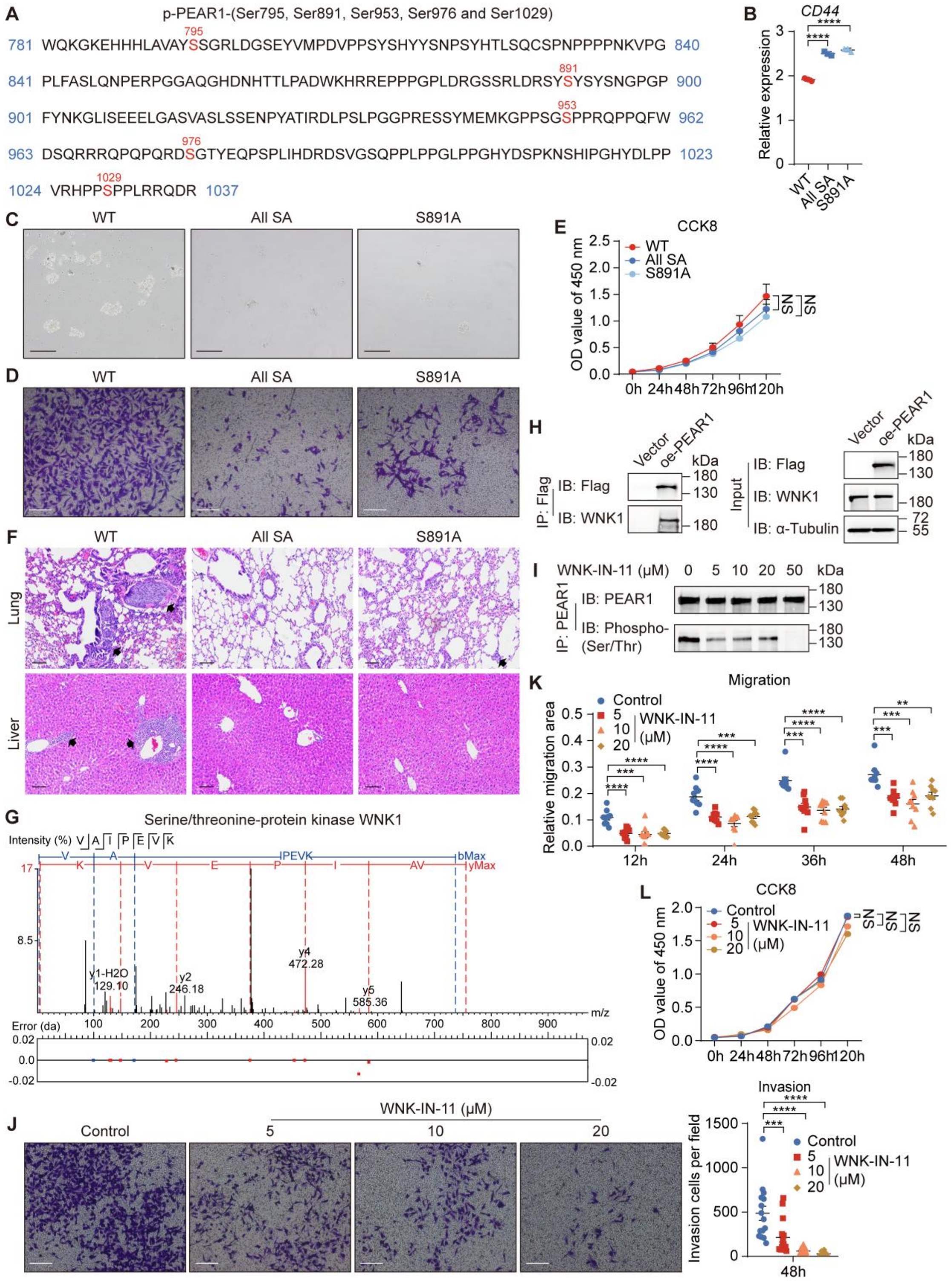

Supplemental Figure 6. PEAR1 phosphorylation at Ser891 is crucial for CD44 function.

(**A**) Five phosphorylated serine residues indicated of PEAR1 were identified in MDA-MB-231 cells by IP-MS analysis.

(**B**) *CD44* mRNA expression levels in indicated MDA-MB-231 cells were detected by RT-qPCR. The bar diagram shows the relative expression of mRNAs normalized to that of *18S rRNA* (n = 3; mean ± SD).

(**C**) Representative images of indicated MDA-MB-231 cells in the mammosphere formation assay. Scale bar: 50 μm.
(**D-E**) Representative images of invasive cells in transwell assay, and viability curves in a CCK8 assay kit of MDA-MB-231 cells indicated (n = 3; mean ± SEM). Scale bar: 100 μm.
(**F**) Representative images of metastatic foci in the lungs and livers of nude mice intravenously injected with indicated MDA-MB-231 cells by H&E staining (n = 5 mice per group). Scale bar: 50 μm.
(**G**) MS analysis of WNK1-specific peptide (VAIPEVK) from co-IP samples (PEAR1-Flag-interacting proteins) of whole-cell lysates of oe-PEAR1-Flag-treated MDA-MB-231 cells using anti-Flag agarose beads. Cells transfected with the corresponding empty vector plasmids were used as negative controls.
(**H**) Co-IP of whole-cell lysates of oe-PEAR1-Flag-treated MDA-MB-231 cells with anti-Flag agarose beads. Cells transfected with the corresponding empty vector plasmids were used as negative controls.
(**I-L**) PEAR1 phosphorylation was detected with western blotting, representative images and quantification of invasive cells in transwell assay, quantification of the relative migration area in wound healing assay, and viability curves in a CCK8 assay kit of MDA-MB-231 cells treated with 0, 5, 10, or 20 μM WNK-IN-11 in a transwell assay (n = 3; mean ± SEM). Scale bar: 100 μm.
One-way ANOVA followed by Dunnett's test was used in (**B**), (**E**), (**J**), (**K**), and (**L**). $^{**}P < 0.01$, $^{***}P < 0.001$, $^{****}P < 0.0001$. The western blotting results were representative of three independent experiments.

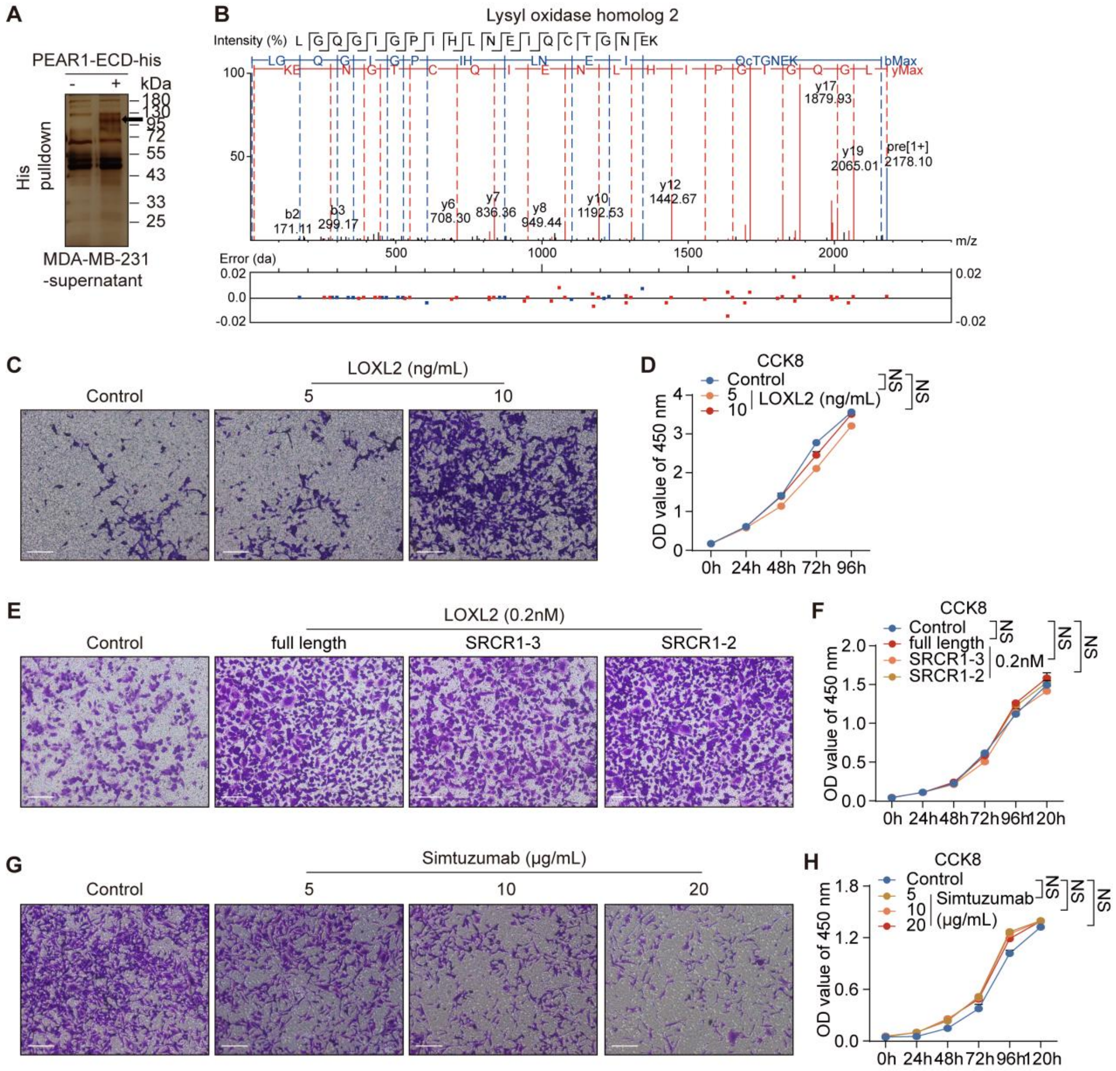

Supplemental Figure 7. LOXL2 triggers PEAR1 phosphorylation.

(**A**) Silver staining of PEAR1-ECD and its interacting proteins pulled down with Dynabeads in the supernatant of MDA-MB-231 cells, in which 15 μg of PEAR1-ECD-his was exogenously added. The arrow pointed to the specific protein bands of PEAR1-ECD interacting proteins compared to the control (PEAR1-ECD-his was not added exogenously). The bands were further to be cut for MS identification.

(**B**) MS analysis of LOXL2-specific peptide (LGQGIGPIHLNEIQCTGNEK) from the specific bands in (**A**).

(**C-D**) Representative images of invasive cells in transwell assay, and viability curves in a CCK8 assay kit of MDA-MB-231 cells treated with 0, 5, or 10 ng/mL LOXL2 (n = 3; mean ± SEM). Scale bar: 100 μm.

(**E-F**) Representative images of invasive cells in transwell assay, and viability curves in a CCK8 assay kit of MDA-MB-231 cells treated with 0.2 nM full-length LOXL2 or truncation LOXL2 (n = 3; mean ± SEM). Scale bar: 100 μm.

(**G-H**) Representative images of invasive cells in transwell assay, and viability curves in a CCK8 assay kit of MDA-MB-231 cells treated with 0, 5, 10, or 20 μg/mL Simtuzumab (n = 3; mean ± SEM). Scale bar: 100 μm.

One-way ANOVA followed by Dunnett's test was used in (**D**), (**F**), and (**H**).

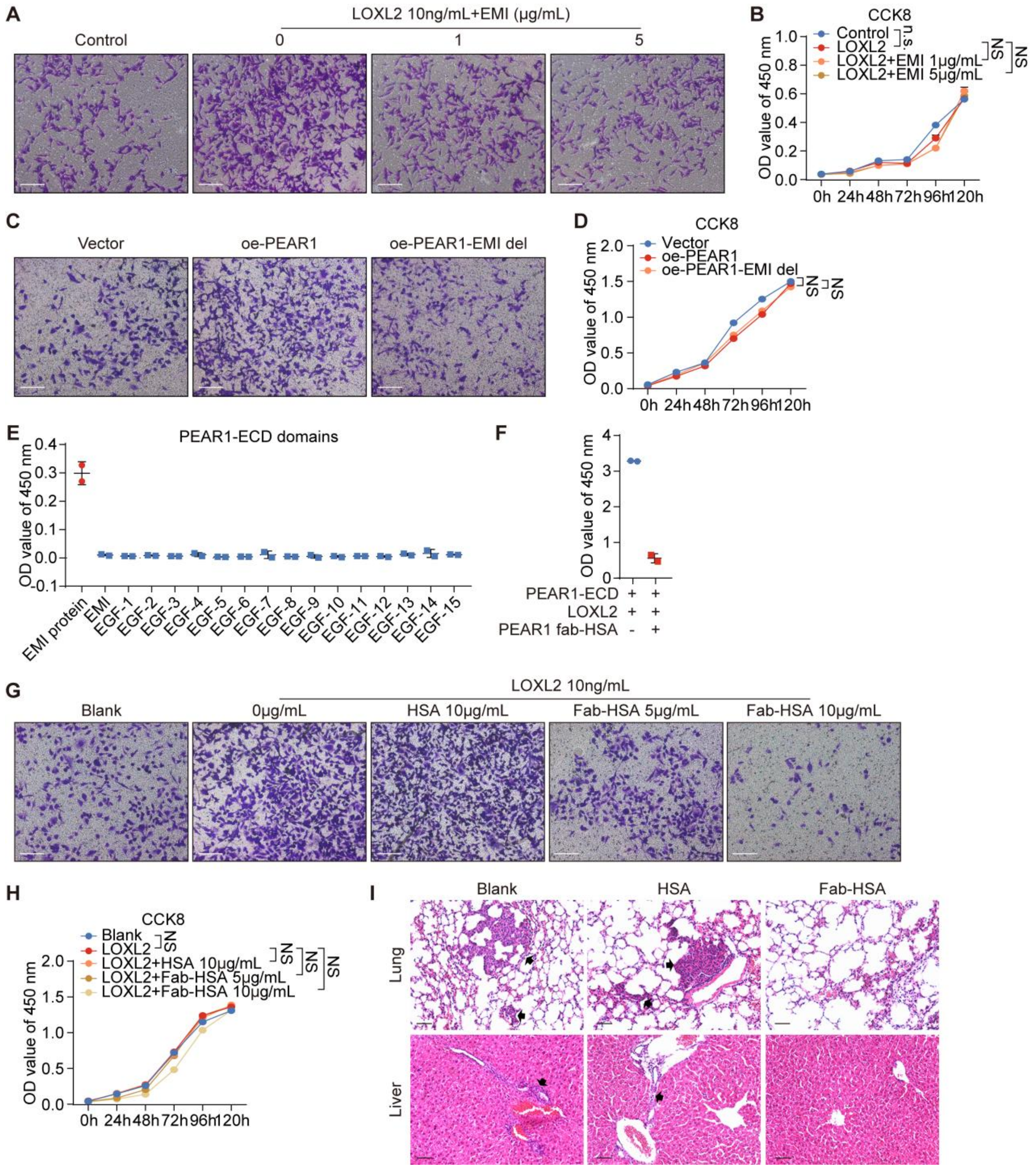

Supplemental Figure 8. Blocking the interaction of LOXL2 with PEAR1 inhibits TNBC metastasis.

(**A-B**) Representative images of invasive cells in transwell assay, and viability curves in a CCK8 assay kit of MDA-MB-231 cells treated with or without 10 ng/mL LOXL2 and 1 or 5 μg/mL PEAR1-EMI domain protein (*n* = 3; mean ± SEM). Scale bar: 100 μm.

(**C-D**) Representative images of invasive cells in transwell assay, and viability curves in a CCK8 assay kit MDA-MB-231 cells with oe-PEAR1-EMI domain deficiency (*n* = 3; mean ± SEM). Scale bar: 100 μm.

(**E**) The interaction between PEAR1 fab-HSA and various PEAR1-ECD domain peptides, PEAR1-EMI domain protein was detected using ELISA (*n* = 2; mean ± SD).

(**F**) The interaction between LOXL2 and PEAR1-ECD protein competitively blocked by 20 μg/mL PEAR1 fab-HSA was detected using ELISA (*n* = 2; mean ± SD).

(**G-H**) Representative images of invasive cells in transwell assay, and viability curves in a CCK8 assay kit MDA-MB-231 cells treated with 10 ng/mL LOXL2 and 5 or 10 μg/mL PEAR1 fab-HSA (*n* = 3; mean ± SEM). Scale bar: 100 μm.
(**I**) Representative images of metastatic foci in the lungs and livers of NOD-SCID mice intravenously injected with MDA-MB-231 cells and treated using PEAR1 fab-HSA at a dose of 3.35 mg/kg for 40 days by H&E staining (*n* = 5 mice per group). Scale bar: 50 μm.
One-way ANOVA followed by Dunnett's test was used in (**B**), (**D**) and (**H**).

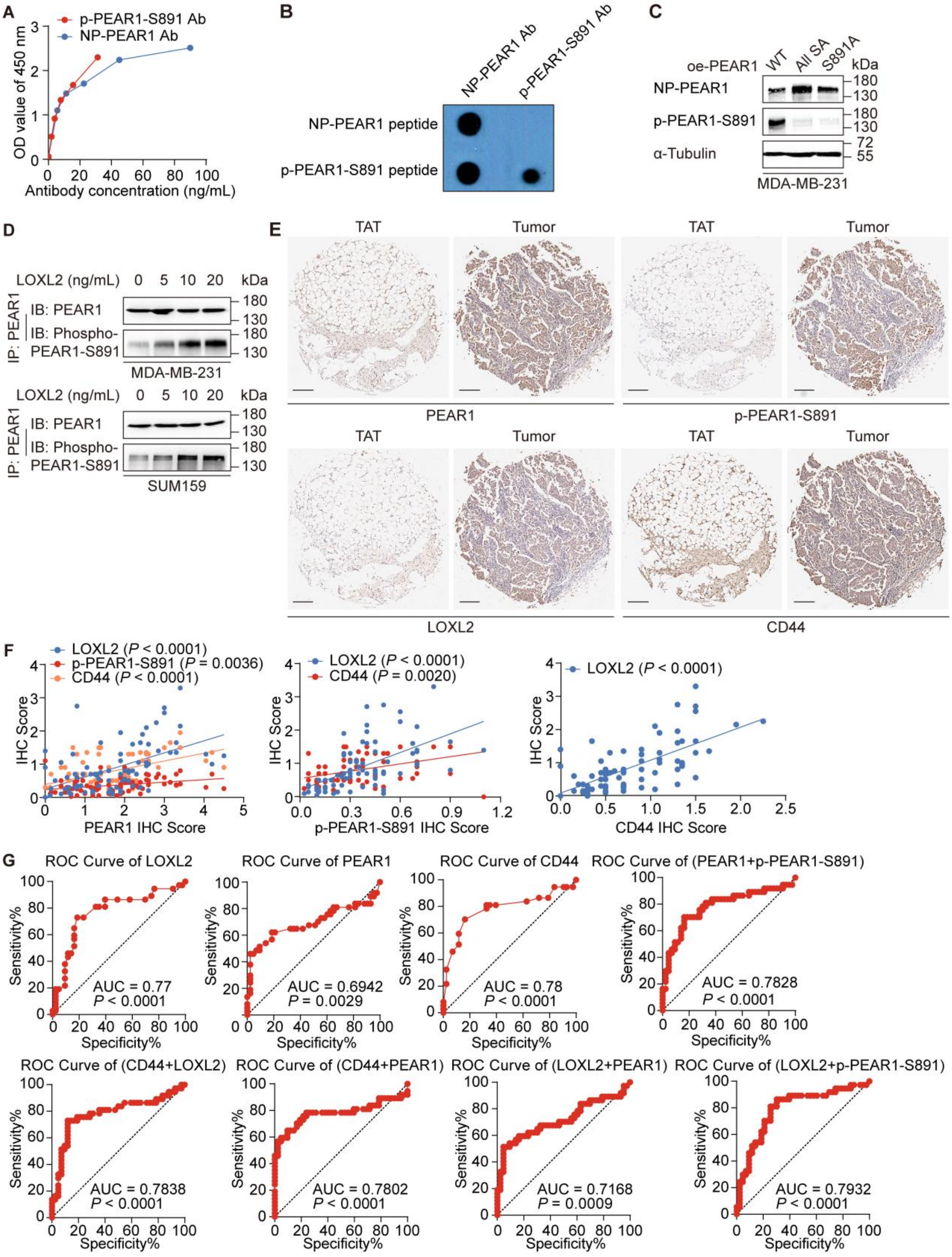

Supplemental Figure 9. The LOXL2/PEAR1/CD44 axis is upregulated in TNBC and is associated with poor overall survival.

(**A**) The interaction between anti-phospho-PEAR1 (Ser891) antibody (p-PEAR1-S891 Ab) and p-PEAR1-S891 peptide, or the interaction between the corresponding anti-nonphosphorylated PEAR1 antibody (NP-PEAR1 Ab) and NP-PEAR1 peptide was detected using ELISA. The polyclonal antibody against phosphorylated PEAR1 at Ser891 was prepared by immunizing rabbit and purified with a synthetic p-PEAR1-S891 peptide.

(**B**) The specificity of the anti-p-PEAR1-S891 Ab was evaluated by dot blotting. The loading content of p-PEAR1-S891 peptide and the corresponding NP-PEAR1 peptide was 7 ng.
(**C**) The expression levels of p-PEAR1-S891 and NP-PEAR1 in total lysates of oe-PEAR1-WT-Flag, oe-PEAR1-All SA-Flag and oe-PEAR1-S891A-Flag MDA-MB-231 cells determined by western blotting analysis. α-Tubulin was used as an internal control.
(**D**) Levels of PEAR1 phosphorylation at Ser891 in MDA-MB-231 cells and SUM159 cells treated with 0, 5, 10, 20 ng/mL LOXL2 for 1 h were detected using western blotting after IP with an anti-PEAR1 antibody.
(**E**) Representative IHC staining of the tissue microarray containing TNBC and corresponding adjacent samples with anti-PEAR1, anti-phospho-PEAR1 (Ser891), anti-LOXL2 and anti-CD44 antibodies. Scale bar: 50 μm.
(**F**) Correlation between the expression levels of PEAR1, phospho-PEAR1 (Ser891), LOXL2 and CD44 in TNBC samples (n = 80; pearson correlation analysis).
(**G**) The prognostic effect of the risk score of LOXL2, PEAR1, phospho-PEAR1 (Ser891), CD44 and their combination with each other on TNBC patients, respectively (time-dependent ROC curve analysis).
Pearson correlation analysis was used in (**F**); time-dependent ROC curve analysis was used in (**G**). The western blotting results were representative of three independent experiments.

Supplemental Tables

Name	Sequences 5’-3’
pCDH-CMV-*PEAR1*-Flag-EF1-copGFP-T2A-puromycin (C-terminal Flag-tagged *PEAR1*)	GAATTCGCCACCATGTCACCGCCTCTGTGTCCCCTCCTTCTC<br>CTGGCTGTGGGCCTGCGGCTGGCTGGAACTCTCAACCCCAG<br>TGATCCCAATACCTGCAGCTTCTGGGAAAGCTTCACTACCAC<br>CACCAAGGAGTCCCACTCCCGCCCCTTCAGCCTGCTCCCCT<br>CAGAGCCCTGCGAGCGGCCCTGGGAGGGCCCCCATACTTGC<br>CCCCAGCCCACGGTTGTATACCGGACCGTGTACCGTCAGGT<br>GGTGAAGACGGACCACCGCCAGCGCCTGCAGTGCTGCCAT<br>GGCTTCTATGAGAGCAGGGGGTTCTGTGTCCCGCTCTGTGC<br>CCAGGAGTGTGTCCATGGCCGTTGTGTGGCACCCAATCAGT<br>GCCAATGTGTGCCAGGCTGGCGGGGCGACGACTGTTCCAGT<br>GAGTGTGCCCCAGGAATGTGGGGGCCACAGTGTGACAAGC<br>CCTGCAGCTGCGGCAACAACAGCTCGTGTGATCCCAAGAGT<br>GGGGTATGTTCTTGCCCTTCTGGTCTGCAGCCCCCGAACTGC<br>CTTCAGCCCTGTACCCCTGGCTACTATGGCCCTGCCTGCCAG<br>TTCCGCTGCCAGTGCCATGGGGCACCCTGCGATCCCCAGAC<br>TGGAGCCTGCTTCTGCCCCGCAGAGAGAACTGGGCCCAGCT<br>GTGACGTGTCCTGTTCCCAGGGCACTTCTGGCTTCTTCTGCC<br>CCAGCACCCATTCTTGCCAAAATGGAGGTGTCTTCCAAACC<br>CCACAGGGCTCCTGCAGCTGCCCCCCTGGCTGGATGGGCAC<br>CATCTGCTCCCTGCCCTGCCCAGAGGGCTTTCACGGACCCA<br>ACTGCTCCCAGGAATGTCGCTGCCACAACGGCGGCCTCTGT<br>GACCGATTCACTGGGCAGTGCCGCTGCGCTCCGGGTTACAC<br>TGGGGATCGGTGCCGGGAGGAGTGCCCGGTGGGCCGCTTT<br>GGGCAGGACTGTGCTGAGACGTGCGACTGCGCCCCGGACG<br>CCCGTTGCTTCCCGGCCAACGGCGCATGTCTGTGCGAACAC<br>GGCTTCACTGGGGACCGCTGCACGGATCGCCTCTGCCCCGA<br>CGGCTTCTACGGTCTCAGCTGCCAGGCCCCCTGCACCTGCG<br>ACCGGGAGCACAGCCTCAGCTGCCACCCGATGAACGGGGA<br>GTGCTCCTGCCTGCCGGGCTGGGCGGGCCTCCACTGCAACG<br>AGAGCTGCCCGCAGGACACGCATGGGCCAGGGTGCCAGGA<br>GCACTGTCTCTGCCTGCACGGTGGCGTCTGCCAGGCTACCA<br>GCGGCCTCTGTCAGTGCGCGCCGGGTTACACGGGCCCTCAC<br>TGTGCTAGTCTTTGTCCTCCTGACACCTACGGTGTCAACTGT<br>TCTGCACGCTGCTCATGTGAAAATGCCATCGCCTGCTCACCC<br>ATCGACGGCGAGTGCGTCTGCAAGGAAGGTTGGCAGCGTG<br>GTAACTGCTCTGTGCCCTGCCCACCCGGAACCTGGGGCTTC<br>AGTTGCAATGCCAGCTGCCAGTGTGCCCATGAGGCAGTCTG<br>CAGCCCCCAAACTGGAGCCTGTACCTGCACCCCTGGGTGGC<br>ATGGGGCCCACTGCCAGCTGCCCTGTCCGAAGGGGCAGTTT<br>GGAGAAGGTTGTGCCAGTCGCTGTGACTGTGACCACTCTGA<br>TGGCTGTGACCCTGTTCATGGACGCTGTCAGTGCCAGGCTG

	GCTGGATGGGTGCCCGCTGCCACCTGTCCTGCCCTGAGGGC<br>TTATGGGGAGTCAACTGTAGCAACACCTGCACCTGCAAGAA<br>TGGGGGCACCTGTCTCCCTGAGAATGGCAACTGCGTGTGTG<br>CACCCGGATTCCGGGGCCCCTCCTGCCAGAGATCCTGTCAG<br>CCTGGCCGCTATGGCAAACGCTGTGTGCCCTGCAAGTGCGC<br>TAACCACTCCTTCTGCCACCCCTCGAACGGGACCTGCTACT<br>GCCTGGCTGGCTGGACAGGCCCCGACTGCTCCCAGCCATGC<br>CCTCCAGGACACTGGGGAGAAAACTGTGCCCAGACCTGCC<br>AATGTCACCATGGTGGGACCTGCCATCCCCAGGATGGGAGC<br>TGTATCTGCCCCCTAGGCTGGACTGGACACCACTGCTTAGA<br>AGGCTGCCCTCTGGGGACATTTGGTGCTAACTGCTCCCAGC<br>CATGCCAGTGTGGTCCTGGAGAAAAGTGCCACCCAGAGAC<br>TGGGGCCTGTGTATGTCCCCCAGGGCACAGTGGTGCACCTT<br>GCAGGATTGGAATCCAGGAGCCCTTTACTGTGATGCCGACC<br>ACTCCAGTAGCGTATAACTCGCTGGGTGCAGTGATTGGCATT<br>GCAGTGCTGGGGTCCCTTGTGGTAGCCCTGGTGGCACTGTT<br>CATTGGCTATCGGCACTGGCAAAAAGGCAAGGAGCACCAC<br>CACCTGGCTGTGGCTTACAGCAGCGGGCGCCTGGACGGCTC<br>CGAGTATGTCATGCCAGATGTCCCTCCGAGCTACAGTCACTA<br>CTACTCCAACCCCAGCTACCACACCCTGTCGCAGTGCTCCC<br>CAAACCCCCCACCCCCTAACAAGGTTCCAGGCCCGCTCTTT<br>GCCAGCCTGCAGAACCCTGAGCGGCCAGGTGGGGCCCAAG<br>GGCATGATAACCACACCACCCTGCCTGCTGACTGGAAGCAC<br>CGCCGGGAGCCCCCTCCAGGGCCTCTGGACAGGGGGAGCA<br>GCCGCCTGGACCGAAGCTACAGCTATAGCTACAGCAATGGC<br>CCAGGCCCATTCTACAATAAAGGGCTCATCTCTGAAGAGGA<br>GCTCGGGGCCAGTGTGGCTTCCCTGAGCAGTGAGAACCCAT<br>ATGCCACCATCCGGGACCTGCCCAGCTTGCCAGGGGGCCCC<br>CGGGAGAGCAGCTACATGGAGATGAAAGGCCCTCCCTCAG<br>GATCTCCCCCCAGGCAGCCTCCTCAGTTCTGGGACAGCCAG<br>AGGCGGCGGCAACCCCAGCCACAGAGAGACAGTGGCACCT<br>ACGAGCAGCCCAGCCCCCTGATCCATGACCGAGACTCTGTG<br>GGCTCCCAGCCCCCTCTGCCTCCGGGCCTACCCCCCGGCCA<br>CTATGACTCACCCAAGAACAGCCACATCCCTGGACATTATGA<br>CTTGCCTCCAGTACGGCATCCCCCATCACCTCCACTTCGACG<br>CCAGGACCGTGATTACAAGGATGACGACGATAAGTGAGGAT<br>CC
pcDNA3.4-*PEAR1*-ICD-Flag (C-terminal Flag-tagged *PEAR1*-ICD)	GCGGCCGCCACCatgggctggagctggatcctgctgttcctcctgagcgtgacagcag<br>gagtgcacagcCGGCACTGGCAGAAAGGTAAAGAACATCATCAT<br>CTTGCTGTTGCATATTCCTCCGGGCGCCTGGACGGATCAGAG<br>TATGTAATGCCAGACGTCCCACCCAGCTATAGCCATTATTACT<br>CAAACCCCTCATACCACACCTTGTCCCAATGCTCTCCTAACC<br>CACCACCCCCAAATAAAGTACCCGGGCCCTTGTTCGCCAGT<br>CTCCAGAACCCAGAACGACCGGGAGGTGCTCAGGGGCACG

	ACAACCACACTACTCTGCCTGCTGACTGGAAACACCGCAGG<br>GAACCGCCCCCCGGGCCGCTGGACAGAGGATCTTCAAGGC<br>TGGACCGATCATACAGCTATTCATATAGCAACGGCCCTGGTC<br>CTTTTTATAATAAGGGCTTGATCTCTGAAGAGGAATTGGGCG<br>CGTCCGTGGCCAGCCTTAGCAGCGAAAATCCATACGCCACT<br>ATACGCGATCTTCCCAGCCTTCCAGGAGGGCCCCGGGAAAG<br>TTCATACATGGAGATGAAGGGTCCTCCCTCCGGCTCTCCACC<br>TAGACAGCCCCCCCAATTTTGGGATAGTCAGCGCCGCAGGC<br>AGCCGCAACCGCAGAGAGACTCCGGGACCTACGAACAACC<br>ATCTCCATTGATACATGACCGGGACAGCGTCGGCTCACAAC<br>CACCGCTCCCGCCTGGGCTGCCGCCTGGACACTATGACTCC<br>CCGAAAAACTCACACATCCCCGGTCACTACGATCTTCCTCC<br>AGTTCGACACCCACCAAGCCCGCCTCTCAGGCGACAGGATC<br>GAGACTACAAGGACGACGATGATAAATGATTCTAGA
pcDNA3.1+-*CD44*-ICD-HA (C-terminal HA-tagged *CD44*-ICD)	AAGCTTGCCACCatgggctggagctggatcctgctgttcctcctgagcgtgacagcag<br>gagtgcacagcAACAGTCGGCGCAGGTGCGGACAAAAAAAGAA<br>ACTCGTGATAAATAGTGGCAATGGTGCCGTTGAAGACCGAA<br>AGCCGTCTGGTCTGAACGGTGAAGCATCCAAAAGCCAAGA<br>GATGGTACATCTCGTAAATAAAGAATCCTCCGAAACACCAG<br>ATCAATTTATGACAGCAGATGAAACACGAAACCTTCAAAAT<br>GTCGATATGAAAATAGGAGTGTACCCCTACGACGTGCCTGA<br>CTACGCATGACTCGAG
pCDH-CMV-*PEAR1* EMI deletion-flag-EF1-copGFP-T2A-puromycin (C-terminal Flag-tagged *PEAR1*)	GAATTCGCCACCATGTCACCGCCTCTGTGTCCCCTCCTTCTC<br>CTGGCTGTGGGCCTGCGGCTGGCTGGAACTCTCAACCCCAG<br>TCTCTGTGCCCAGGAGTGTGTCCATGGCCGTTGTGTGGCAC<br>CCAATCAGTGCCAATGTGTGCCAGGCTGGCGGGGCGACGAC<br>TGTTCCAGTGAGTGTGCCCCAGGAATGTGGGGGCCACAGTG<br>TGACAAGCCCTGCAGCTGCGGCAACAACAGCTCGTGTGATC<br>CCAAGAGTGGGGTATGTTCTTGCCCTTCTGGTCTGCAGCCC<br>CCGAACTGCCTTCAGCCCTGTACCCCTGGCTACTATGGCCCT<br>GCCTGCCAGTTCCGCTGCCAGTGCCATGGGGCACCCTGCGA<br>TCCCCAGACTGGAGCCTGCTTCTGCCCCGCAGAGAGAACTG<br>GGCCCAGCTGTGACGTGTCCTGTTCCCAGGGCACTTCTGGC<br>TTCTTCTGCCCCAGCACCCATTCTTGCCAAAATGGAGGTGTC<br>TTCCAAACCCCACAGGGCTCCTGCAGCTGCCCCCCTGGCTG<br>GATGGGCACCATCTGCTCCCTGCCCTGCCCAGAGGGCTTTC<br>ACGGACCCAACTGCTCCCAGGAATGTCGCTGCCACAACGGC<br>GGCCTCTGTGACCGATTCACTGGGCAGTGCCGCTGCGCTCC<br>GGGTTACACTGGGGATCGGTGCCGGGAGGAGTGCCCGGTG<br>GGCCGCTTTGGGCAGGACTGTGCTGAGACGTGCGACTGCG<br>CCCCGGACGCCCGTTGCTTCCCGGCCAACGGCGCATGTCTG<br>TGCGAACACGGCTTCACTGGGGACCGCTGCACGGATCGCCT<br>CTGCCCCGACGGCTTCTACGGTCTCAGCTGCCAGGCCCCCT<br>GCACCTGCGACCGGGAGCACAGCCTCAGCTGCCACCCGAT

	GAACGGGGAGTGCTCCTGCCTGCCGGGCTGGGCGGGCCTC<br>CACTGCAACGAGAGCTGCCCGCAGGACACGCATGGGCCAG<br>GGTGCCAGGAGCACTGTCTCTGCCTGCACGGTGGCGTCTGC<br>CAGGCTACCAGCGGCCTCTGTCAGTGCGCGCCGGGTTACAC<br>GGGCCCTCACTGTGCTAGTCTTTGTCCTCCTGACACCTACGG<br>TGTCAACTGTTCTGCACGCTGCTCATGTGAAAATGCCATCGC<br>CTGCTCACCCATCGACGGCGAGTGCGTCTGCAAGGAAGGTT<br>GGCAGCGTGGTAACTGCTCTGTGCCCTGCCCACCCGGAACC<br>TGGGGCTTCAGTTGCAATGCCAGCTGCCAGTGTGCCCATGA<br>GGCAGTCTGCAGCCCCCAAACTGGAGCCTGTACCTGCACCC<br>CTGGGTGGCATGGGGCCCACTGCCAGCTGCCCTGTCCGAAG<br>GGGCAGTTTGGAGAAGGTTGTGCCAGTCGCTGTGACTGTGA<br>CCACTCTGATGGCTGTGACCCTGTTCATGGACGCTGTCAGT<br>GCCAGGCTGGCTGGATGGGTGCCCGCTGCCACCTGTCCTGC<br>CCTGAGGGCTTATGGGGAGTCAACTGTAGCAACACCTGCAC<br>CTGCAAGAATGGGGGCACCTGTCTCCCTGAGAATGGCAACT<br>GCGTGTGTGCACCCGGATTCCGGGGCCCCTCCTGCCAGAGA<br>TCCTGTCAGCCTGGCCGCTATGGCAAACGCTGTGTGCCCTG<br>CAAGTGCGCTAACCACTCCTTCTGCCACCCCTCGAACGGGA<br>CCTGCTACTGCCTGGCTGGCTGGACAGGCCCCGACTGCTCC<br>CAGCCATGCCCTCCAGGACACTGGGGAGAAAACTGTGCCC<br>AGACCTGCCAATGTCACCATGGTGGGACCTGCCATCCCCAG<br>GATGGGAGCTGTATCTGCCCCCTAGGCTGGACTGGACACCA<br>CTGCTTAGAAGGCTGCCCTCTGGGGACATTTGGTGCTAACT<br>GCTCCCAGCCATGCCAGTGTGGTCCTGGAGAAAAGTGCCAC<br>CCAGAGACTGGGGCCTGTGTATGTCCCCCAGGGCACAGTGG<br>TGCACCTTGCAGGATTGGAATCCAGGAGCCCTTTACTGTGAT<br>GCCGACCACTCCAGTAGCGTATAACTCGCTGGGTGCAGTGA<br>TTGGCATTGCAGTGCTGGGGTCCCTTGTGGTAGCCCTGGTG<br>GCACTGTTCATTGGCTATCGGCACTGGCAAAAAGGCAAGGA<br>GCACCACCACCTGGCTGTGGCTTACAGCAGCGGGCGCCTGG<br>ACGGCTCCGAGTATGTCATGCCAGATGTCCCTCCGAGCTACA<br>GTCACTACTACTCCAACCCCAGCTACCACACCCTGTCGCAG<br>TGCTCCCCAAACCCCCCACCCCCTAACAAGGTTCCAGGCCC<br>GCTCTTTGCCAGCCTGCAGAACCCTGAGCGGCCAGGTGGG<br>GCCCAAGGGCATGATAACCACACCACCCTGCCTGCTGACTG<br>GAAGCACCGCCGGGAGCCCCCTCCAGGGCCTCTGGACAGG<br>GGGAGCAGCCGCCTGGACCGAAGCTACAGCTATAGCTACAG<br>CAATGGCCCAGGCCCATTCTACAATAAAGGGCTCATCTCTGA<br>AGAGGAGCTCGGGGCCAGTGTGGCTTCCCTGAGCAGTGAG<br>AACCCATATGCCACCATCCGGGACCTGCCCAGCTTGCCAGG<br>GGGCCCCCGGGAGAGCAGCTACATGGAGATGAAAGGCCCT<br>CCCTCAGGATCTCCCCCCAGGCAGCCTCCTCAGTTCTGGGA<br>CAGCCAGAGGCGGCGGCAACCCCAGCCACAGAGAGACAGT

	GGCACCTACGAGCAGCCAGCCCCTGATCCATGACCGAGA<br>CTCTGTGGGCTCCCAGCCCCCTCTGCCTCCGGGCCTACCCC<br>CCGGCCACTATGACTCACCCAAGAACAGCCACATCCCTGGA<br>CATTATGACTTGCCTCCAGTACGGCATCCCCCATCACCTCCA<br>CTTCGACGCCAGGACCGTGATTACAAGGATGACGACGATAA<br>GTGAGGATCC
pCDH-CMV-*PEAR1*-S795A-flag-EF1-copGFP-T2A-puromycin (C-terminal Flag-tagged *PEAR1*)	GAATTCGCCACCATGTCACCGCCTCTGTGTCCCCTCCTTCTC<br>CTGGCTGTGGGCCTGCGGCTGGCTGGAACTCTCAACCCCAG<br>TGATCCCAATACCTGCAGCTTCTGGGAAAGCTTCACTACCAC<br>CACCAAGGAGTCCCACTCCCGCCCCTTCAGCCTGCTCCCCT<br>CAGAGCCCTGCGAGCGGCCCTGGGAGGGCCCCCATACTTGC<br>CCCCAGCCCACGGTTGTATACCGGACCGTGTACCGTCAGGT<br>GGTGAAGACGGACCACCGCCAGCGCCTGCAGTGCTGCCAT<br>GGCTTCTATGAGAGCAGGGGGTTCTGTGTCCCGCTCTGTGC<br>CCAGGAGTGTGTCCATGGCCGTTGTGTGGCACCCAATCAGT<br>GCCAATGTGTGCCAGGCTGGCGGGGCGACGACTGTTCCAGT<br>GAGTGTGCCCCAGGAATGTGGGGGCCACAGTGTGACAAGC<br>CCTGCAGCTGCGGCAACAACAGCTCGTGTGATCCCAAGAGT<br>GGGGTATGTTCTTGCCCTTCTGGTCTGCAGCCCCCGAACTGC<br>CTTCAGCCCTGTACCCCTGGCTACTATGGCCCTGCCTGCCAG<br>TTCCGCTGCCAGTGCCATGGGGCACCCTGCGATCCCCAGAC<br>TGGAGCCTGCTTCTGCCCCGCAGAGAGAACTGGGCCCAGCT<br>GTGACGTGTCCTGTTCCCAGGGCACTTCTGGCTTCTTCTGCC<br>CCAGCACCCATTCTTGCCAAAATGGAGGTGTCTTCCAAACC<br>CCACAGGGCTCCTGCAGCTGCCCCCCTGGCTGGATGGGCAC<br>CATCTGCTCCCTGCCCTGCCCAGAGGGCTTTCACGGACCCA<br>ACTGCTCCCAGGAATGTCGCTGCCACAACGGCGGCCTCTGT<br>GACCGATTCACTGGGCAGTGCCGCTGCGCTCCGGGTTACAC<br>TGGGGATCGGTGCCGGGAGGAGTGCCCGGTGGGCCGCTTT<br>GGGCAGGACTGTGCTGAGACGTGCGACTGCGCCCCGGACG<br>CCCGTTGCTTCCCGGCCAACGGCGCATGTCTGTGCGAACAC<br>GGCTTCACTGGGGACCGCTGCACGGATCGCCTCTGCCCCGA<br>CGGCTTCTACGGTCTCAGCTGCCAGGCCCCCTGCACCTGCG<br>ACCGGGAGCACAGCCTCAGCTGCCACCCGATGAACGGGGA<br>GTGCTCCTGCCTGCCGGGCTGGGCGGGCCTCCACTGCAACG<br>AGAGCTGCCCGCAGGACACGCATGGGCCAGGGTGCCAGGA<br>GCACTGTCTCTGCCTGCACGGTGGCGTCTGCCAGGCTACCA<br>GCGGCCTCTGTCAGTGCGCGCCGGGTTACACGGGCCCTCAC<br>TGTGCTAGTCTTTGTCCTCCTGACACCTACGGTGTCAACTGT<br>TCTGCACGCTGCTCATGTGAAAATGCCATCGCCTGCTCACCC<br>ATCGACGGCGAGTGCGTCTGCAAGGAAGGTTGGCAGCGTG<br>GTAACTGCTCTGTGCCCTGCCCACCCGGAACCTGGGGCTTC<br>AGTTGCAATGCCAGCTGCCAGTGTGCCCATGAGGCAGTCTG<br>CAGCCCCCAAACTGGAGCCTGTACCTGCACCCCTGGGTGGC

	ATGGGGCCCACTGCCAGCTGCCCTGTCCGAAGGGGCAGTTT<br>GGAGAAGGTTGTGCCAGTCGCTGTGACTGTGACCACTCTGA<br>TGGCTGTGACCCTGTTCATGGACGCTGTCAGTGCCAGGCTG<br>GCTGGATGGGTGCCCGCTGCCACCTGTCCTGCCCTGAGGGC<br>TTATGGGGAGTCAACTGTAGCAACACCTGCACCTGCAAGAA<br>TGGGGGCACCTGTCTCCCTGAGAATGGCAACTGCGTGTGTG<br>CACCCGGATTCCGGGGCCCCTCCTGCCAGAGATCCTGTCAG<br>CCTGGCCGCTATGGCAAACGCTGTGTGCCCTGCAAGTGCGC<br>TAACCACTCCTTCTGCCACCCCTCGAACGGGACCTGCTACT<br>GCCTGGCTGGCTGGACAGGCCCCGACTGCTCCCAGCCATGC<br>CCTCCAGGACACTGGGGAGAAAACTGTGCCCAGACCTGCC<br>AATGTCACCATGGTGGGACCTGCCATCCCCAGGATGGGAGC<br>TGTATCTGCCCCCTAGGCTGGACTGGACACCACTGCTTAGA<br>AGGCTGCCCTCTGGGGACATTTGGTGCTAACTGCTCCCAGC<br>CATGCCAGTGTGGTCCTGGAGAAAAGTGCCACCCAGAGAC<br>TGGGGCCTGTGTATGTCCCCCAGGGCACAGTGGTGCACCTT<br>GCAGGATTGGAATCCAGGAGCCCTTTACTGTGATGCCGACC<br>ACTCCAGTAGCGTATAACTCGCTGGGTGCAGTGATTGGCATT<br>GCAGTGCTGGGGTCCCTTGTGGTAGCCCTGGTGGCACTGTT<br>CATTGGCTATCGGCACTGGCAAAAAGGCAAGGAGCACCAC<br>CACCTGGCTGTGGCTTACGCAAGCGGGCGCCTGGACGGCTC<br>CGAGTATGTCATGCCAGATGTCCCTCCGAGCTACAGTCACTA<br>CTACTCCAACCCCAGCTACCACACCCTGTCGCAGTGCTCCC<br>CAAACCCCCCACCCCCTAACAAGGTTCCAGGCCCGCTCTTT<br>GCCAGCCTGCAGAACCCTGAGCGGCCAGGTGGGGCCCAAG<br>GGCATGATAACCACACCACCCTGCCTGCTGACTGGAAGCAC<br>CGCCGGGAGCCCCCTCCAGGGCCTCTGGACAGGGGGAGCA<br>GCCGCCTGGACCGAAGCTACAGCTATAGCTACAGCAATGGC<br>CCAGGCCCATTCTACAATAAAGGGCTCATCTCTGAAGAGGA<br>GCTCGGGGCCAGTGTGGCTTCCCTGAGCAGTGAGAACCCAT<br>ATGCCACCATCCGGGACCTGCCCAGCTTGCCAGGGGGCCCC<br>CGGGAGAGCAGCTACATGGAGATGAAAGGCCCTCCCTCAG<br>GATCTCCCCCCAGGCAGCCTCCTCAGTTCTGGGACAGCCAG<br>AGGCGGCGGCAACCCCAGCCACAGAGAGACAGTGGCACCT<br>ACGAGCAGCCCAGCCCCCTGATCCATGACCGAGACTCTGTG<br>GGCTCCCAGCCCCCTCTGCCTCCGGGCCTACCCCCCGGCCA<br>CTATGACTCACCCAAGAACAGCCACATCCCTGGACATTATGA<br>CTTGCCTCCAGTACGGCATCCCCCATCACCTCCACTTCGACG<br>CCAGGACCGTGATTACAAGGATGACGACGATAAGTGAGGAT<br>CC
pCDH-CMV-<br>*PEAR1*-S891<br>A-flag-EF1-c<br>opGFP-T2A-	GAATTCGCCACCATGTCACCGCCTCTGTGTCCCCTCCTTCTC<br>CTGGCTGTGGGCCTGCGGCTGGCTGGAACTCTCAACCCCAG<br>TGATCCCAATACCTGCAGCTTCTGGGAAAGCTTCACTACCAC<br>CACCAAGGAGTCCCACTCCCGCCCCTTCAGCCTGCTCCCCT

puromycin<br>(C-terminal<br>Flag-tagged<br>*PEAR1*)	CAGAGCCCTGCGAGCGGCCCTGGGAGGGCCCCCATACTTGC<br>CCCCAGCCCACGGTTGTATACCGGACCGTGTACCGTCAGGT<br>GGTGAAGACGGACCACCGCCAGCGCCTGCAGTGCTGCCAT<br>GGCTTCTATGAGAGCAGGGGGTTCTGTGTCCCGCTCTGTGC<br>CCAGGAGTGTGTCCATGGCCGTTGTGTGGCACCCAATCAGT<br>GCCAATGTGTGCCAGGCTGGCGGGGCGACGACTGTTCCAGT<br>GAGTGTGCCCCAGGAATGTGGGGGCCACAGTGTGACAAGC<br>CCTGCAGCTGCGGCAACAACAGCTCGTGTGATCCCAAGAGT<br>GGGGTATGTTCTTGCCCTTCTGGTCTGCAGCCCCCGAACTGC<br>CTTCAGCCCTGTACCCCTGGCTACTATGGCCCTGCCTGCCAG<br>TTCCGCTGCCAGTGCCATGGGGCACCCTGCGATCCCCAGAC<br>TGGAGCCTGCTTCTGCCCCGCAGAGAGAACTGGGCCCAGCT<br>GTGACGTGTCCTGTTCCCAGGGCACTTCTGGCTTCTTCTGCC<br>CCAGCACCCATTCTTGCCAAAATGGAGGTGTCTTCCAAACC<br>CCACAGGGCTCCTGCAGCTGCCCCCCTGGCTGGATGGGCAC<br>CATCTGCTCCCTGCCCTGCCCAGAGGGCTTTCACGGACCCA<br>ACTGCTCCCAGGAATGTCGCTGCCACAACGGCGGCCTCTGT<br>GACCGATTCACTGGGCAGTGCCGCTGCGCTCCGGGTTACAC<br>TGGGGATCGGTGCCGGGAGGAGTGCCCGGTGGGCCGCTTT<br>GGGCAGGACTGTGCTGAGACGTGCGACTGCGCCCCGGACG<br>CCCGTTGCTTCCCGGCCAACGGCGCATGTCTGTGCGAACAC<br>GGCTTCACTGGGGACCGCTGCACGGATCGCCTCTGCCCCGA<br>CGGCTTCTACGGTCTCAGCTGCCAGGCCCCCTGCACCTGCG<br>ACCGGGAGCACAGCCTCAGCTGCCACCCGATGAACGGGGA<br>GTGCTCCTGCCTGCCGGGCTGGGCGGGCCTCCACTGCAACG<br>AGAGCTGCCCGCAGGACACGCATGGGCCAGGGTGCCAGGA<br>GCACTGTCTCTGCCTGCACGGTGGCGTCTGCCAGGCTACCA<br>GCGGCCTCTGTCAGTGCGCGCCGGGTTACACGGGCCCTCAC<br>TGTGCTAGTCTTTGTCCTCCTGACACCTACGGTGTCAACTGT<br>TCTGCACGCTGCTCATGTGAAAATGCCATCGCCTGCTCACCC<br>ATCGACGGCGAGTGCGTCTGCAAGGAAGGTTGGCAGCGTG<br>GTAACTGCTCTGTGCCCTGCCCACCCGGAACCTGGGGCTTC<br>AGTTGCAATGCCAGCTGCCAGTGTGCCCATGAGGCAGTCTG<br>CAGCCCCCAAACTGGAGCCTGTACCTGCACCCCTGGGTGGC<br>ATGGGGCCCACTGCCAGCTGCCCTGTCCGAAGGGGCAGTTT<br>GGAGAAGGTTGTGCCAGTCGCTGTGACTGTGACCACTCTGA<br>TGGCTGTGACCCTGTTCATGGACGCTGTCAGTGCCAGGCTG<br>GCTGGATGGGTGCCCGCTGCCACCTGTCCTGCCCTGAGGGC<br>TTATGGGGAGTCAACTGTAGCAACACCTGCACCTGCAAGAA<br>TGGGGGCACCTGTCTCCCTGAGAATGGCAACTGCGTGTGTG<br>CACCCGGATTCCGGGGCCCCTCCTGCCAGAGATCCTGTCAG<br>CCTGGCCGCTATGGCAAACGCTGTGTGCCCTGCAAGTGCGC<br>TAACCACTCCTTCTGCCACCCCTCGAACGGACCTGCTACT<br>GCCTGGCTGGCTGGACAGGCCCCGACTGCTCCCAGCCATGC

	CCTCCAGGACACTGGGGAGAAAACTGTGCCCAGACCTGCC AATGTCACCATGGTGGGACCTGCCATCCCCAGGATGGGAGC TGTATCTGCCCCCTAGGCTGGACTGGACACCACTGCTTAGA AGGCTGCCCTCTGGGGACATTTGGTGCTAACTGCTCCCAGC CATGCCAGTGTGGTCCTGGAGAAAAGTGCCACCCAGAGAC TGGGGCCTGTGTATGTCCCCCAGGGCACAGTGGTGCACCTT GCAGGATTGGAATCCAGGAGCCCTTTACTGTGATGCCGACC ACTCCAGTAGCGTATAACTCGCTGGGTGCAGTGATTGGCATT GCAGTGCTGGGGTCCCTTGTGGTAGCCCTGGTGGCACTGTT CATTGGCTATCGGCACTGGCAAAAAGGCAAGGAGCACCAC CACCTGGCTGTGGCTTACAGCAGCGGGCGCCTGGACGGCTC CGAGTATGTCATGCCAGATGTCCCTCCGAGCTACAGTCACTA CTACTCCAACCCCAGCTACCACACCCTGTCGCAGTGCTCCC CAAACCCCCCACCCCCTAACAAGGTTCCAGGCCCGCTCTTT GCCAGCCTGCAGAACCCTGAGCGGCCAGGTGGGGCCCAAG GGCATGATAACCACACCACCCTGCCTGCTGACTGGAAGCAC CGCCGGGAGCCCCCTCCAGGGCCTCTGGACAGGGGGAGCA GCCGCCTGGACCGAAGCTACGCCTATAGCTACAGCAATGGC CCAGGCCCATTCTACAATAAAGGGCTCATCTCTGAAGAGGA GCTCGGGGCCAGTGTGGCTTCCCTGAGCAGTGAGAACCCAT ATGCCACCATCCGGGACCTGCCCAGCTTGCCAGGGGGCCCC CGGGAGAGCAGCTACATGGAGATGAAAGGCCCTCCCTCAG GATCTCCCCCCAGGCAGCCTCCTCAGTTCTGGGACAGCCAG AGGCGGCGGCAACCCCAGCCACAGAGAGACAGTGGCACCT ACGAGCAGCCCAGCCCCCTGATCCATGACCGAGACTCTGTG GGCTCCCAGCCCCCTCTGCCTCCGGGCCTACCCCCCGGCCA CTATGACTCACCCAAGAACAGCCACATCCCTGGACATTATGA CTTGCCTCCAGTACGGCATCCCCCATCACCTCCACTTCGACG CCAGGACCGTGATTACAAGGATGACGACGATAAGTGAGGAT CC
pCDH-CMV-*PEAR1*-S953A-flag-EF1-copGFP-T2A-puromycin (C-terminal Flag-tagged *PEAR1*)	GAATTCGCCACCATGTCACCGCCTCTGTGTCCCCTCCTTCTC CTGGCTGTGGGCCTGCGGCTGGCTGGAACTCTCAACCCCAG TGATCCCAATACCTGCAGCTTCTGGGAAAGCTTCACTACCAC CACCAAGGAGTCCCACTCCCGCCCCTTCAGCCTGCTCCCCT CAGAGCCCTGCGAGCGGCCCTGGGAGGGCCCCCATACTTGC CCCCAGCCCACGGTTGTATACCGGACCGTGTACCGTCAGGT GGTGAAGACGGACCACCGCCAGCGCCTGCAGTGCTGCCAT GGCTTCTATGAGAGCAGGGGGTTCTGTGTCCCGCTCTGTGC CCAGGAGTGTGTCCATGGCCGTTGTGTGGCACCCAATCAGT GCCAATGTGTGCCAGGCTGGCGGGGCGACGACTGTTCCAGT GAGTGTGCCCCAGGAATGTGGGGGCCACAGTGTGACAAGC CCTGCAGCTGCGGCAACAACAGCTCGTGTGATCCCAAGAGT GGGGTATGTTCTTGCCCTTCTGGTCTGCAGCCCCCGAACTGC CTTCAGCCCTGTACCCCTGGCTACTATGGCCCTGCCTGCCAG

	TTCCGCTGCCAGTGCCATGGGGCACCCTGCGATCCCCAGAC<br>TGGAGCCTGCTTCTGCCCCGCAGAGAGAACTGGGCCCAGCT<br>GTGACGTGTCCTGTTCCCAGGGCACTTCTGGCTTCTTCTGCC<br>CCAGCACCCATTCTTGCCAAAATGGAGGTGTCTTCCAAACC<br>CCACAGGGCTCCTGCAGCTGCCCCCCTGGCTGGATGGGCAC<br>CATCTGCTCCCTGCCCTGCCCAGAGGGCTTTCACGGACCCA<br>ACTGCTCCCAGGAATGTCGCTGCCACAACGGCGGCCTCTGT<br>GACCGATTCACTGGGCAGTGCCGCTGCGCTCCGGGTTACAC<br>TGGGGATCGGTGCCGGGAGGAGTGCCCGGTGGGCCGCTTT<br>GGGCAGGACTGTGCTGAGACGTGCGACTGCGCCCCGGACG<br>CCCGTTGCTTCCCGGCCAACGGCGCATGTCTGTGCGAACAC<br>GGCTTCACTGGGGACCGCTGCACGGATCGCCTCTGCCCCGA<br>CGGCTTCTACGGTCTCAGCTGCCAGGCCCCCTGCACCTGCG<br>ACCGGGAGCACAGCCTCAGCTGCCACCCGATGAACGGGGA<br>GTGCTCCTGCCTGCCGGGCTGGGCGGGCCTCCACTGCAACG<br>AGAGCTGCCCGCAGGACACGCATGGGCCAGGGTGCCAGGA<br>GCACTGTCTCTGCCTGCACGGTGGCGTCTGCCAGGCTACCA<br>GCGGCCTCTGTCAGTGCGCGCCGGGTTACACGGGCCCTCAC<br>TGTGCTAGTCTTTGTCCTCCTGACACCTACGGTGTCAACTGT<br>TCTGCACGCTGCTCATGTGAAAATGCCATCGCCTGCTCACCC<br>ATCGACGGCGAGTGCGTCTGCAAGGAAGGTTGGCAGCGTG<br>GTAACTGCTCTGTGCCCTGCCCACCCGGAACCTGGGGCTTC<br>AGTTGCAATGCCAGCTGCCAGTGTGCCCATGAGGCAGTCTG<br>CAGCCCCCAAACTGGAGCCTGTACCTGCACCCCTGGGTGGC<br>ATGGGGCCCACTGCCAGCTGCCCTGTCCGAAGGGGCAGTTT<br>GGAGAAGGTTGTGCCAGTCGCTGTGACTGTGACCACTCTGA<br>TGGCTGTGACCCTGTTCATGGACGCTGTCAGTGCCAGGCTG<br>GCTGGATGGGTGCCCGCTGCCACCTGTCCTGCCCTGAGGGC<br>TTATGGGGAGTCAACTGTAGCAACACCTGCACCTGCAAGAA<br>TGGGGGCACCTGTCTCCCTGAGAATGGCAACTGCGTGTGTG<br>CACCCGGATTCCGGGGCCCCTCCTGCCAGAGATCCTGTCAG<br>CCTGGCCGCTATGGCAAACGCTGTGTGCCCTGCAAGTGCGC<br>TAACCACTCCTTCTGCCACCCCTCGAACGGGACCTGCTACT<br>GCCTGGCTGGCTGGACAGGCCCCGACTGCTCCCAGCCATGC<br>CCTCCAGGACACTGGGGAGAAAACTGTGCCCAGACCTGCC<br>AATGTCACCATGGTGGGACCTGCCATCCCCAGGATGGGAGC<br>TGTATCTGCCCCCTAGGCTGGACTGGACACCACTGCTTAGA<br>AGGCTGCCCTCTGGGGACATTTGGTGCTAACTGCTCCCAGC<br>CATGCCAGTGTGGTCCTGGAGAAAAGTGCCACCCAGAGAC<br>TGGGGCCTGTGTATGTCCCCCAGGGCACAGTGGTGCACCTT<br>GCAGGATTGGAATCCAGGAGCCCTTTACTGTGATGCCGACC<br>ACTCCAGTAGCGTATAACTCGCTGGGTGCAGTGATTGGCATT<br>GCAGTGCTGGGGTCCCTTGTGGTAGCCCTGGTGGCACTGTT<br>CATTGGCTATCGGCACTGGCAAAAAGGCAAGGAGCACCAC

	CACCTGGCTGTGGCTTACAGCAGCGGGCGCCTGGACGGCTC<br>CGAGTATGTCATGCCAGATGTCCCTCCGAGCTACAGTCACTA<br>CTACTCCAACCCCAGCTACCACACCCTGTCGCAGTGCTCCC<br>CAAACCCCCCACCCCCTAACAAGGTTCCAGGCCCGCTCTTT<br>GCCAGCCTGCAGAACCCTGAGCGGCCAGGTGGGGCCCAAG<br>GGCATGATAACCACACCACCCTGCCTGCTGACTGGAAGCAC<br>CGCCGGGAGCCCCCTCCAGGGCCTCTGGACAGGGGGAGCA<br>GCCGCCTGGACCGAAGCTACAGCTATAGCTACAGCAATGGC<br>CCAGGCCCATTCTACAATAAAGGGCTCATCTCTGAAGAGGA<br>GCTCGGGGCCAGTGTGGCTTCCCTGAGCAGTGAGAACCCAT<br>ATGCCACCATCCGGGACCTGCCCAGCTTGCCAGGGGGCCCC<br>CGGGAGAGCAGCTACATGGAGATGAAAGGCCCTCCCTCAG<br>GAGCACCCCCCAGGCAGCCTCCTCAGTTCTGGGACAGCCA<br>GAGGCGGCGGCAACCCCAGCCACAGAGAGACAGTGGCACC<br>TACGAGCAGCCCAGCCCCCTGATCCATGACCGAGACTCTGT<br>GGGCTCCCAGCCCCCTCTGCCTCCGGGCCTACCCCCCGGCC<br>ACTATGACTCACCCAAGAACAGCCACATCCCTGGACATTATG<br>ACTTGCCTCCAGTACGGCATCCCCCATCACCTCCACTTCGAC<br>GCCAGGACCGTGATTACAAGGATGACGACGATAAGTGAGGA<br>TCC
pCDH-CMV-*PEAR1*-S976A-flag-EF1-copGFP-T2A-puromycin (C-terminal Flag-tagged *PEAR1*)	GAATTCGCCACCATGTCACCGCCTCTGTGTCCCCTCCTTCTC<br>CTGGCTGTGGGCCTGCGGCTGGCTGGAACTCTCAACCCCAG<br>TGATCCCAATACCTGCAGCTTCTGGGAAAGCTTCACTACCAC<br>CACCAAGGAGTCCCACTCCCGCCCCTTCAGCCTGCTCCCCT<br>CAGAGCCCTGCGAGCGGCCCTGGGAGGGCCCCCATACTTGC<br>CCCCAGCCCACGGTTGTATACCGGACCGTGTACCGTCAGGT<br>GGTGAAGACGGACCACCGCCAGCGCCTGCAGTGCTGCCAT<br>GGCTTCTATGAGAGCAGGGGGTTCTGTGTCCCGCTCTGTGC<br>CCAGGAGTGTGTCCATGGCCGTTGTGTGGCACCCAATCAGT<br>GCCAATGTGTGCCAGGCTGGCGGGGCGACGACTGTTCCAGT<br>GAGTGTGCCCCAGGAATGTGGGGGCCACAGTGTGACAAGC<br>CCTGCAGCTGCGGCAACAACAGCTCGTGTGATCCCAAGAGT<br>GGGGTATGTTCTTGCCCTTCTGGTCTGCAGCCCCCGAACTGC<br>CTTCAGCCCTGTACCCCTGGCTACTATGGCCCTGCCTGCCAG<br>TTCCGCTGCCAGTGCCATGGGGCACCCTGCGATCCCCAGAC<br>TGGAGCCTGCTTCTGCCCCGCAGAGAGAACTGGGCCCAGCT<br>GTGACGTGTCCTGTTCCCAGGGCACTTCTGGCTTCTTCTGCC<br>CCAGCACCCATTCTTGCCAAAATGGAGGTGTCTTCCAAACC<br>CCACAGGGCTCCTGCAGCTGCCCCCCTGGCTGGATGGGCAC<br>CATCTGCTCCCTGCCCTGCCCAGAGGGCTTTCACGGACCCA<br>ACTGCTCCCAGGAATGTCGCTGCCACAACGGCGGCCTCTGT<br>GACCGATTCACTGGGCAGTGCCGCTGCGCTCCGGGTTACAC<br>TGGGGATCGGTGCCGGGAGGAGTGCCCGGTGGGCCGCTTT<br>GGGCAGGACTGTGCTGAGACGTGCGACTGCGCCCCGGACG

	CCCGTTGCTTCCCGGCCAACGGCGCATGTCTGTGCGAACAC<br>GGCTTCACTGGGGACCGCTGCACGGATCGCCTCTGCCCCGA<br>CGGCTTCTACGGTCTCAGCTGCCAGGCCCCCTGCACCTGCG<br>ACCGGGAGCACAGCCTCAGCTGCCACCCGATGAACGGGGA<br>GTGCTCCTGCCTGCCGGGCTGGGCGGGCCTCCACTGCAACG<br>AGAGCTGCCCGCAGGACACGCATGGGCCAGGGTGCCAGGA<br>GCACTGTCTCTGCCTGCACGGTGGCGTCTGCCAGGCTACCA<br>GCGGCCTCTGTCAGTGCGCGCCGGGTTACACGGGCCCTCAC<br>TGTGCTAGTCTTTGTCCTCCTGACACCTACGGTGTCAACTGT<br>TCTGCACGCTGCTCATGTGAAAATGCCATCGCCTGCTCACCC<br>ATCGACGGCGAGTGCGTCTGCAAGGAAGGTTGGCAGCGTG<br>GTAACTGCTCTGTGCCCTGCCCACCCGGAACCTGGGGCTTC<br>AGTTGCAATGCCAGCTGCCAGTGTGCCCATGAGGCAGTCTG<br>CAGCCCCCAAACTGGAGCCTGTACCTGCACCCCTGGGTGGC<br>ATGGGGCCCACTGCCAGCTGCCCTGTCCGAAGGGGCAGTTT<br>GGAGAAGGTTGTGCCAGTCGCTGTGACTGTGACCACTCTGA<br>TGGCTGTGACCCTGTTCATGGACGCTGTCAGTGCCAGGCTG<br>GCTGGATGGGTGCCCGCTGCCACCTGTCCTGCCCTGAGGGC<br>TTATGGGGAGTCAACTGTAGCAACACCTGCACCTGCAAGAA<br>TGGGGGCACCTGTCTCCCTGAGAATGGCAACTGCGTGTGTG<br>CACCCGGATTCCGGGGCCCCTCCTGCCAGAGATCCTGTCAG<br>CCTGGCCGCTATGGCAAACGCTGTGTGCCCTGCAAGTGCGC<br>TAACCACTCCTTCTGCCACCCCTCGAACGGGACCTGCTACT<br>GCCTGGCTGGCTGGACAGGCCCCGACTGCTCCCAGCCATGC<br>CCTCCAGGACACTGGGGAGAAAACTGTGCCCAGACCTGCC<br>AATGTCACCATGGTGGGACCTGCCATCCCCAGGATGGGAGC<br>TGTATCTGCCCCCTAGGCTGGACTGGACACCACTGCTTAGA<br>AGGCTGCCCTCTGGGGACATTTGGTGCTAACTGCTCCCAGC<br>CATGCCAGTGTGGTCCTGGAGAAAAGTGCCACCCAGAGAC<br>TGGGGCCTGTGTATGTCCCCCAGGGCACAGTGGTGCACCTT<br>GCAGGATTGGAATCCAGGAGCCCTTTACTGTGATGCCGACC<br>ACTCCAGTAGCGTATAACTCGCTGGGTGCAGTGATTGGCATT<br>GCAGTGCTGGGGTCCCTTGTGGTAGCCCTGGTGGCACTGTT<br>CATTGGCTATCGGCACTGGCAAAAAGGCAAGGAGCACCAC<br>CACCTGGCTGTGGCTTACAGCAGCGGGCGCCTGGACGGCTC<br>CGAGTATGTCATGCCAGATGTCCCTCCGAGCTACAGTCACTA<br>CTACTCCAACCCCAGCTACCACACCCTGTCGCAGTGCTCCC<br>CAAACCCCCCACCCCCTAACAAGGTTCCAGGCCCGCTCTTT<br>GCCAGCCTGCAGAACCCTGAGCGGCCAGGTGGGGCCCAAG<br>GGCATGATAACCACACCACCCTGCCTGCTGACTGGAAGCAC<br>CGCCGGGAGCCCCCTCCAGGGCCTCTGGACAGGGGGAGCA<br>GCCGCCTGGACCGAAGCTACAGCTATAGCTACAGCAATGGC<br>CCAGGCCCATTCTACAATAAAGGGCTCATCTCTGAAGAGGA<br>GCTCGGGGCCAGTGTGGCTTCCCTGAGCAGTGAGAACCCAT

	ATGCCACCATCCGGGACCTGCCCAGCTTGCCAGGGGGCCCC<br>CGGGAGAGCAGCTACATGGAGATGAAAGGCCCTCCCTCAG<br>GATCTCCCCCCAGGCAGCCTCCTCAGTTCTGGGACAGCCAG<br>AGGCGGCGGCAACCCCAGCCACAGAGAGACGCAGGCACCT<br>ACGAGCAGCCCAGCCCCCTGATCCATGACCGAGACTCTGTG<br>GGCTCCCAGCCCCCTCTGCCTCCGGGCCTACCCCCCGGCCA<br>CTATGACTCACCCAAGAACAGCCACATCCCTGGACATTATGA<br>CTTGCCTCCAGTACGGCATCCCCCATCACCTCCACTTCGACG<br>CCAGGACCGTGATTACAAGGATGACGACGATAAGTGAGGAT<br>CC
pCDH-CMV-*PEAR1*-S1029A-flag-EF1-copGFP-T2A-puromycin (C-terminal Flag-tagged *PEAR1*)	GAATTCGCCACCATGTCACCGCCTCTGTGTCCCCTCCTTCTC<br>CTGGCTGTGGGCCTGCGGCTGGCTGGAACTCTCAACCCCAG<br>TGATCCCAATACCTGCAGCTTCTGGGAAAGCTTCACTACCAC<br>CACCAAGGAGTCCCACTCCCGCCCCTTCAGCCTGCTCCCCT<br>CAGAGCCCTGCGAGCGGCCCTGGGAGGGCCCCCATACTTGC<br>CCCCAGCCCACGGTTGTATACCGGACCGTGTACCGTCAGGT<br>GGTGAAGACGGACCACCGCCAGCGCCTGCAGTGCTGCCAT<br>GGCTTCTATGAGAGCAGGGGGTTCTGTGTCCCGCTCTGTGC<br>CCAGGAGTGTGTCCATGGCCGTTGTGTGGCACCCAATCAGT<br>GCCAATGTGTGCCAGGCTGGCGGGGCGACGACTGTTCCAGT<br>GAGTGTGCCCCAGGAATGTGGGGGCCACAGTGTGACAAGC<br>CCTGCAGCTGCGGCAACAACAGCTCGTGTGATCCCAAGAGT<br>GGGGTATGTTCTTGCCCTTCTGGTCTGCAGCCCCCGAACTGC<br>CTTCAGCCCTGTACCCCTGGCTACTATGGCCCTGCCTGCCAG<br>TTCCGCTGCCAGTGCCATGGGGCACCCTGCGATCCCCAGAC<br>TGGAGCCTGCTTCTGCCCCGCAGAGAGAACTGGGCCCAGCT<br>GTGACGTGTCCTGTTCCCAGGGCACTTCTGGCTTCTTCTGCC<br>CCAGCACCCATTCTTGCCAAAATGGAGGTGTCTTCCAAACC<br>CCACAGGGCTCCTGCAGCTGCCCCCCTGGCTGGATGGGCAC<br>CATCTGCTCCCTGCCCTGCCCAGAGGGCTTTCACGGACCCA<br>ACTGCTCCCAGGAATGTCGCTGCCACAACGGCGGCCTCTGT<br>GACCGATTCACTGGGCAGTGCCGCTGCGCTCCGGGTTACAC<br>TGGGGATCGGTGCCGGGAGGAGTGCCCGGTGGGCCGCTTT<br>GGGCAGGACTGTGCTGAGACGTGCGACTGCGCCCCGGACG<br>CCCGTTGCTTCCCGGCCAACGGCGCATGTCTGTGCGAACAC<br>GGCTTCACTGGGGACCGCTGCACGGATCGCCTCTGCCCCGA<br>CGGCTTCTACGGTCTCAGCTGCCAGGCCCCCTGCACCTGCG<br>ACCGGGAGCACAGCCTCAGCTGCCACCCGATGAACGGGGA<br>GTGCTCCTGCCTGCCGGGCTGGGCGGGCCTCCACTGCAACG<br>AGAGCTGCCCGCAGGACACGCATGGGCCAGGGTGCCAGGA<br>GCACTGTCTCTGCCTGCACGGTGGCGTCTGCCAGGCTACCA<br>GCGGCCTCTGTCAGTGCGCGCCGGGTTACACGGGCCCTCAC<br>TGTGCTAGTCTTTGTCCTCCTGACACCTACGGTGTCAACTGT<br>TCTGCACGCTGCTCATGTGAAAATGCCATCGCCTGCTCACCC

	ATCGACGGCGAGTGCGTCTGCAAGGAAGGTTGGCAGCGTG<br>GTAACTGCTCTGTGCCCTGCCCACCCGGAACCTGGGGCTTC<br>AGTTGCAATGCCAGCTGCCAGTGTGCCCATGAGGCAGTCTG<br>CAGCCCCCAAACTGGAGCCTGTACCTGCACCCCTGGGTGGC<br>ATGGGGCCCACTGCCAGCTGCCCTGTCCGAAGGGGCAGTTT<br>GGAGAAGGTTGTGCCAGTCGCTGTGACTGTGACCACTCTGA<br>TGGCTGTGACCCTGTTCATGGACGCTGTCAGTGCCAGGCTG<br>GCTGGATGGGTGCCCGCTGCCACCTGTCCTGCCCTGAGGGC<br>TTATGGGGAGTCAACTGTAGCAACACCTGCACCTGCAAGAA<br>TGGGGGCACCTGTCTCCCTGAGAATGGCAACTGCGTGTGTG<br>CACCCGGATTCCGGGGCCCCTCCTGCCAGAGATCCTGTCAG<br>CCTGGCCGCTATGGCAAACGCTGTGTGCCCTGCAAGTGCGC<br>TAACCACTCCTTCTGCCACCCCTCGAACGGGACCTGCTACT<br>GCCTGGCTGGCTGGACAGGCCCCGACTGCTCCCAGCCATGC<br>CCTCCAGGACACTGGGGAGAAAACTGTGCCCAGACCTGCC<br>AATGTCACCATGGTGGGACCTGCCATCCCCAGGATGGGAGC<br>TGTATCTGCCCCCTAGGCTGGACTGGACACCACTGCTTAGA<br>AGGCTGCCCTCTGGGGACATTTGGTGCTAACTGCTCCCAGC<br>CATGCCAGTGTGGTCCTGGAGAAAAGTGCCACCCAGAGAC<br>TGGGGCCTGTGTATGTCCCCCAGGGCACAGTGGTGCACCTT<br>GCAGGATTGGAATCCAGGAGCCCTTTACTGTGATGCCGACC<br>ACTCCAGTAGCGTATAACTCGCTGGGTGCAGTGATTGGCATT<br>GCAGTGCTGGGGTCCCTTGTGGTAGCCCTGGTGGCACTGTT<br>CATTGGCTATCGGCACTGGCAAAAAGGCAAGGAGCACCAC<br>CACCTGGCTGTGGCTTACAGCAGCGGGCGCCTGGACGGCTC<br>CGAGTATGTCATGCCAGATGTCCCTCCGAGCTACAGTCACTA<br>CTACTCCAACCCCAGCTACCACACCCTGTCGCAGTGCTCCC<br>CAAACCCCCCACCCCCTAACAAGGTTCCAGGCCCGCTCTTT<br>GCCAGCCTGCAGAACCCTGAGCGGCCAGGTGGGGCCCAAG<br>GGCATGATAACCACACCACCCTGCCTGCTGACTGGAAGCAC<br>CGCCGGGAGCCCCCTCCAGGGCCTCTGGACAGGGGGAGCA<br>GCCGCCTGGACCGAAGCTACAGCTATAGCTACAGCAATGGC<br>CCAGGCCCATTCTACAATAAAGGGCTCATCTCTGAAGAGGA<br>GCTCGGGGCCAGTGTGGCTTCCCTGAGCAGTGAGAACCCAT<br>ATGCCACCATCCGGGACCTGCCCAGCTTGCCAGGGGGCCCC<br>CGGGAGAGCAGCTACATGGAGATGAAAGGCCCTCCCTCAG<br>GATCTCCCCCCAGGCAGCCTCCTCAGTTCTGGGACAGCCAG<br>AGGCGGCGGCAACCCCAGCCACAGAGAGACAGTGGCACCT<br>ACGAGCAGCCCAGCCCCCTGATCCATGACCGAGACTCTGTG<br>GGCTCCCAGCCCCCTCTGCCTCCGGGCCTACCCCCCGGCCA<br>CTATGACTCACCCAAGAACAGCCACATCCCTGGACATTATGA<br>CTTGCCTCCAGTACGGCATCCCCCAGCTCCTCCACTTCGACG<br>CCAGGACCGTGATTACAAGGATGACGACGATAAGTGAGGAT<br>CC

pCDH-CMV-*PEAR1*-all SA-flag-EF1-copGFP-T2A -puromycin (C-terminal Flag-tagged *PEAR1*)	GAATTCGCCACCATGTCACCGCCTCTGTGTCCCCTCCTTCTC<br>CTGGCTGTGGGCCTGCGGCTGGCTGGAACTCTCAACCCCAG<br>TGATCCCAATACCTGCAGCTTCTGGGAAAGCTTCACTACCAC<br>CACCAAGGAGTCCCACTCCCGCCCCTTCAGCCTGCTCCCCT<br>CAGAGCCCTGCGAGCGGCCCTGGGAGGGCCCCCATACTTGC<br>CCCCAGCCCACGGTTGTATACCGGACCGTGTACCGTCAGGT<br>GGTGAAGACGGACCACCGCCAGCGCCTGCAGTGCTGCCAT<br>GGCTTCTATGAGAGCAGGGGGTTCTGTGTCCCGCTCTGTGC<br>CCAGGAGTGTGTCCATGGCCGTTGTGTGGCACCCAATCAGT<br>GCCAATGTGTGCCAGGCTGGCGGGGCGACGACTGTTCCAGT<br>GAGTGTGCCCCAGGAATGTGGGGGCCACAGTGTGACAAGC<br>CCTGCAGCTGCGGCAACAACAGCTCGTGTGATCCCAAGAGT<br>GGGGTATGTTCTTGCCCTTCTGGTCTGCAGCCCCCGAACTGC<br>CTTCAGCCCTGTACCCCTGGCTACTATGGCCCTGCCTGCCAG<br>TTCCGCTGCCAGTGCCATGGGGCACCCTGCGATCCCCAGAC<br>TGGAGCCTGCTTCTGCCCCGCAGAGAGAACTGGGCCCAGCT<br>GTGACGTGTCCTGTTCCCAGGGCACTTCTGGCTTCTTCTGCC<br>CCAGCACCCATTCTTGCCAAAATGGAGGTGTCTTCCAAACC<br>CCACAGGGCTCCTGCAGCTGCCCCCCTGGCTGGATGGGCAC<br>CATCTGCTCCCTGCCCTGCCCAGAGGGCTTTCACGGACCCA<br>ACTGCTCCCAGGAATGTCGCTGCCACAACGGCGGCCTCTGT<br>GACCGATTCACTGGGCAGTGCCGCTGCGCTCCGGGTTACAC<br>TGGGGATCGGTGCCGGGAGGAGTGCCCGGTGGGCCGCTTT<br>GGGCAGGACTGTGCTGAGACGTGCGACTGCGCCCCGGACG<br>CCCGTTGCTTCCCGGCCAACGGCGCATGTCTGTGCGAACAC<br>GGCTTCACTGGGGACCGCTGCACGGATCGCCTCTGCCCCGA<br>CGGCTTCTACGGTCTCAGCTGCCAGGCCCCCTGCACCTGCG<br>ACCGGGAGCACAGCCTCAGCTGCCACCCGATGAACGGGGA<br>GTGCTCCTGCCTGCCGGGCTGGGCGGGCCTCCACTGCAACG<br>AGAGCTGCCCGCAGGACACGCATGGGCCAGGGTGCCAGGA<br>GCACTGTCTCTGCCTGCACGGTGGCGTCTGCCAGGCTACCA<br>GCGGCCTCTGTCAGTGCGCGCCGGGTTACACGGGCCCTCAC<br>TGTGCTAGTCTTTGTCCTCCTGACACCTACGGTGTCAACTGT<br>TCTGCACGCTGCTCATGTGAAAATGCCATCGCCTGCTCACCC<br>ATCGACGGCGAGTGCGTCTGCAAGGAAGGTTGGCAGCGTG<br>GTAACTGCTCTGTGCCCTGCCCACCCGGAACCTGGGGCTTC<br>AGTTGCAATGCCAGCTGCCAGTGTGCCCATGAGGCAGTCTG<br>CAGCCCCCAAACTGGAGCCTGTACCTGCACCCCTGGGTGGC<br>ATGGGGCCCACTGCCAGCTGCCCTGTCCGAAGGGGCAGTTT<br>GGAGAAGGTTGTGCCAGTCGCTGTGACTGTGACCACTCTGA<br>TGGCTGTGACCCTGTTCATGGACGCTGTCAGTGCCAGGCTG<br>GCTGGATGGGTGCCCGCTGCCACCTGTCCTGCCCTGAGGGC<br>TTATGGGGAGTCAACTGTAGCAACACCTGCACCTGCAAGAA<br>TGGGGGCACCTGTCTCCCTGAGAATGGCAACTGCGTGTGTG

	CACCCGGATTCCGGGGCCCCTCCTGCCAGAGATCCTGTCAG<br>CCTGGCCGCTATGGCAAACGCTGTGTGCCCTGCAAGTGCGC<br>TAACCACTCCTTCTGCCACCCCTCGAACGGACCTGCTACT<br>GCCTGGCTGGCTGGACAGGCCCCGACTGCTCCCAGCCATGC<br>CCTCCAGGACACTGGGGAGAAAACTGTGCCCAGACCTGCC<br>AATGTCACCATGGTGGGACCTGCCATCCCCAGGATGGGAGC<br>TGTATCTGCCCCCTAGGCTGGACTGGACACCACTGCTTAGA<br>AGGCTGCCCTCTGGGGACATTTGGTGCTAACTGCTCCCAGC<br>CATGCCAGTGTGGTCCTGGAGAAAAGTGCCACCCAGAGAC<br>TGGGGCCTGTGTATGTCCCCCAGGGCACAGTGGTGCACCTT<br>GCAGGATTGGAATCCAGGAGCCCTTTACTGTGATGCCGACC<br>ACTCCAGTAGCGTATAACTCGCTGGGTGCAGTGATTGGCATT<br>GCAGTGCTGGGGTCCCTTGTGGTAGCCCTGGTGGCACTGTT<br>CATTGGCTATCGGCACTGGCAAAAAGGCAAGGAGCACCAC<br>CACCTGGCTGTGGCTTACGCAAGCGGGCGCCTGGACGGCTC<br>CGAGTATGTCATGCCAGATGTCCCTCCGAGCTACAGTCACTA<br>CTACTCCAACCCCAGCTACCACACCCTGTCGCAGTGCTCCC<br>CAAACCCCCCACCCCCTAACAAGGTTCCAGGCCCGCTCTTT<br>GCCAGCCTGCAGAACCCTGAGCGGCCAGGTGGGGCCCAAG<br>GGCATGATAACCACACCACCCTGCCTGCTGACTGGAAGCAC<br>CGCCGGGAGCCCCCTCCAGGGCCTCTGGACAGGGGGAGCA<br>GCCGCCTGGACCGAAGCTACGCCTATAGCTACAGCAATGGC<br>CCAGGCCCATTCTACAATAAAGGGCTCATCTCTGAAGAGGA<br>GCTCGGGGCCAGTGTGGCTTCCCTGAGCAGTGAGAACCCAT<br>ATGCCACCATCCGGGACCTGCCCAGCTTGCCAGGGGGCCCC<br>CGGGAGAGCAGCTACATGGAGATGAAAGGCCCTCCCTCAG<br>GAGCACCCCCCAGGCAGCCTCCTCAGTTCTGGGACAGCCA<br>GAGGCGGCGGCAACCCCAGCCACAGAGAGACGCAGGCACC<br>TACGAGCAGCCCAGCCCCCTGATCCATGACCGAGACTCTGT<br>GGGCTCCCAGCCCCCTCTGCCTCCGGGCCTACCCCCCGGCC<br>ACTATGACTCACCCAAGAACAGCCACATCCCTGGACATTATG<br>ACTTGCCTCCAGTACGGCATCCCCCAGCTCCTCCACTTCGAC<br>GCCAGGACCGTGATTACAAGGATGACGACGATAAGTGAGGA<br>TCC
shRNA targeting sequence: *PEAR1* #1	ggACCGAAGCTACAGCTATAG
shRNA targeting sequence: *PEAR1* #2	gaCCACTCCAGTAGCGTATAA
shRNA targeting	gcAATGCAAGTGTAACAACAA

sequence: *Pear1* #205	
shRNA targeting sequence: *Pear1* #206	gcCCGTTGCTTTCCTGCTAAT
shRNA targeting sequence: *CD44* #1	AAGCTCTGAGCATCGGATTTG
shRNA targeting sequence: *CD44* #2	TTGCAGTCAACAGTCGAAGAA
shRNA targeting sequence: Control	TTCTCCGAACGTGTCACGT

Supplemental Table 1. The sequences of plasmids and shRNA used in the study.

Name	Survival (Yes or No)	Lifetime (months)	Pathological type
A1	Y	150	1
A2	N	32	1
A3	Y	148	/
A4	Y	148	1
A5	N	147	1
A6	Y	147	1
A7	Y	147	/
A8	N	33	4
A9	Y	146	1
A10	N	54	4
A11	Y	145	3
A12	N	93	1
A13	Y	145	1
A14	N	114	1
A15	Y	145	/
A16	N	97	4
B1	Y	144	2
B2	Y	143	1
B3	Y	143	3
B4	N	19	/
B5	Y	143	/

B6	N	18	4
B7	N	17	3
B8	Y	143	1
B9	N	11	4
B10	N	82	4
B11	Y	142	2
B12	Y	142	1
B13	Y	141	/
B14	Y	141	1
B15	Y	141	1
B16	Y	141	1
C1	Y	141	1
C2	Y	141	2
C3	Y	140	1
C4	Y	140	1
C5	Y	139	4
C6	Y	138	1
C7	Y	138	/
C8	N	32	1
C9	Y	138	1
C10	Y	137	1
C11	Y	137	1
C12	N	7	/
C13	Y	137	2
C14	Y	137	3
C15	Y	137	2
C16	Y	136	2
D1	Y	136	/
D2	N	125	/
D3	N	23	3
D4	Y	135	4
D5	Y	135	1
D6	Y	135	1
D7	Y	135	3
D8	N	47	/
D9	N	77	3
D10	Y	134	1
D11	Y	134	1
D12	N	92	/
D13	N	31	1
D14	N	78	/
D15	Y	133	/
D16	Y	132	/

E1	Y	132	1
E2	Y	132	/
E3	Y	132	1
E4	N	53	1
E5	Y	131	/
E6	Y	131	/
E7	Y	131	1
E8	Y	130	/
E9	N	23	/
E10	Y	130	/
E11	Y	130	/
E12	N	15	/
E13	Y	129	1
E14	N	60	1
E15	N	110	1
E16	Y	128	2
F1	Y	128	/
F2	N	63	1
F3	N	35	4
F4	Y	128	/
F5	Y	128	/
F6	N	44	1
F7	N	78	/
F8	Y	128	1
F9	Y	127	1
F10	N	4	4
F11	N	4	/
F12	Y	126	1
F13	N	62	4
F14	Y	120	3
F15	Y	120	1
F16	N	72	/
G1	N	19	/
G2	N	2	4
G3	N	110	4
G4	Y	118	2
G5	Y	118	1
G6	Y	117	3
G7	Y	117	2
G8	N	85	/
G9	Y	116	3
G10	Y	115	1
G11	Y	115	/

G12	N	68	3
G13	N	61	/
G14	Y	115	/
G15	Y	115	4
G16	Y	114	3
H1	Y	114	2
H2	Y	114	/
H3	Y	113	/
H4	Y	112	/
H5	Y	112	/
H6	Y	112	3
H7	Y	112	4
H8	Y	112	1
H9	Y	112	/
H10	Y	111	/
H11	Y	111	/
H12	Y	111	2
H13	Y	111	1
H14	Y	110	2
H15	Y	110	/
H16	N	79	2
I1	N	80	/
I2	Y	110	/
I3	Y	110	/
I4	N	46	4
I5	Y	109	1
I6	N	59	1
I7	Y	109	/
I8	Y	109	/
I9	Y	109	/
I10	Y	109	1
I11	Y	109	/
I12	Y	108	/
I13	Y	108	/
I14	Y	108	4
I15	N	9	2
I16	Y	108	2
J1	Y	107	1

Supplemental Table 2. Clinical and pathological characteristics of human breast tumor tissues.

Y: Yes, N: No. “1”: Lumina A (ER+PR+HER2-), “2”: Lumina B (ER+PR+HER2+), “3”: HER2+ER-PR-, “4”: TNBC (ER-PR-HER2-).

Name	Survival (Yes or No)	Lifetime (days)	Sample type
A1	/	/	TAT
A2	Y	2843	T
A3	N	4514	T
A4	/	/	TAT
A5	N	5073	T
A6	/	/	TAT
A7	N	4327	T
A8	/	/	TAT
A9	Y	1879	T
A10	/	/	TAT
A11	Y	1959	T
A12	/	/	TAT
A13	N	5563	T
A14	/	/	TAT
A15	N	4355	T
A16	/	/	TAT
B1	Y	1920	T
B2	/	/	TAT
B3	N	2823	T
B4	/	/	TAT
B5	/	/	TAT
B6	N	3961	T
B7	N	3861	T
B8	/	/	TAT
B9	Y	2916	T
B10	/	/	TAT
B11	N	4199	T
B12	/	/	TAT
B13	N	4228	T
B14	/	/	TAT
B15	N	2476	T
B16	/	/	TAT
C1	N	3961	T
C2	/	/	TAT
C3	N	4675	T
C4	/	/	TAT
C5	N	4090	T
C6	/	/	TAT
C7	N	4115	T
C8	/	/	TAT
C9	N	4278	T

C10	/	/	TAT
C11	Y	1838	T
C12	/	/	TAT
C13	/	/	TAT
C14	N	4183	T
C15	Y	2238	T
C16	/	/	TAT
D1	Y	3134	T
D2	/	/	TAT
D3	/	/	TAT
D4	N	5360	T
D5	/	/	TAT
D6	N	4629	T
D7	N	4226	T
D8	/	/	TAT
D9	N	2470	T
D10	/	/	TAT
D11	Y	1856	T
D12	/	/	TAT
D13	N	4022	T
D14	/	/	TAT
D15	/	/	TAT
D16	N	5198	T
E1	N	3494	T
E2	/	/	TAT
E3	N	4251	T
E4	/	/	TAT
E5	N	4640	T
E6	/	/	TAT
E7	N	3135	T
E8	/	/	TAT
E9	Y	3422	T
E10	/	/	TAT
E11	Y	1434	T
E12	/	/	TAT
E13	Y	2069	T
E14	/	/	TAT
E15	/	/	TAT
E16	N	3610	T
F1	N	3306	T
F2	/	/	TAT
F3	N	3898	T
F4	/	/	TAT

F5	Y	2333	T
F6	/	/	TAT
F7	Y	2336	T
F8	/	/	TAT
F9	N	3946	T
F10	/	/	TAT
F11	Y	2467	T
F12	/	/	TAT
F13	N	3795	T
F14	/	/	TAT
F15	/	/	TAT
F16	N	5122	T
G1	N	5054	T
G2	/	/	TAT
G3	N	3966	T
G4	/	/	TAT
G5	/	/	TAT
G6	Y	3048	T
G7	N	3229	T
G8	/	/	TAT
G9	N	4029	T
G10	/	/	TAT
G11	Y	1721	T
G12	/	/	TAT
G13	N	3721	T
G14	/	/	TAT
G15	N	3866	T
G16	/	/	TAT
H1	Y	1803	T
H2	/	/	TAT
H3	Y	1902	T
H4	/	/	TAT
H5	N	3831	T
H6	/	/	TAT
H7	N	4963	T
H8	/	/	TAT
H9	N	2793	T
H10	/	/	TAT
H11	Y	3024	T
H12	/	/	TAT
H13	Y	2366	T
H14	/	/	TAT
H15	N	3550	T

H16	/	/	TAT
I1	Y	1889	T
I2	/	/	TAT
I3	/	/	TAT
I4	Y	2980	T
I5	N	3671	T
I6	/	/	TAT
I7	Y	2871	T
I8	/	/	TAT
I9	N	4199	T
I10	/	/	TAT
I11	N	4594	T
I12	/	/	TAT
I13	N	5530	T
I14	/	/	TAT
I15	N	4137	T
I16	/	/	TAT
J1	/	/	TAT
J2	N	3832	T
J3	N	4995	T
J4	/	/	TAT
J5	N	3611	T
J6	/	/	TAT
J7	N	3587	T
J8	/	/	TAT
J9	/	/	TAT
J10	N	5407	T
J11	N	4624	T
J12	/	/	TAT
J13	Y	1446	T
J14	/	/	TAT
J15	N	4030	T
J16	/	/	TAT

Supplemental Table 3. Clinical characteristics of human TNBC and corresponding paracancer tissues.

Y: Yes, N: No. "T": Tumor, "TAT": Tumor-adjacent tissues.

Name	Sequences 5'-3'
human *PEAR1*-F	CAGTTTTCTCCCCAGTGTCC
human *PEAR1*-R	GCGCTAACCACTCCTTCTG
human *CD44*-F	CTGCCGCTTTGCAGGTGTA
human *CD44*-R	CATTGTGGGCAAGGTGCTATT
human *18S rRNA*-F	ACCCGTTGAACCCCATTCGTGA

human *18S rRNA*-R	GCCTCACTAAACCATCCAATCGG
mouse *Pear1*-F	CACCACGACCACTAAGGAGTC
mouse *Pear1*-R	ACAGTCCGGTAGACAACCGTA
mouse *18S rRNA*-F	TTGACTCAACACGGGAAACC
mouse *18S rRNA*-R	AGACAAATCGCTCCACCAAC

Supplemental Table 4. The sequences of PCR primers used in the study.
F: forward, R: reverse.

Name	Sequences
Protein: PEAR1 EMI-his	DPNTCSFWESFTTTTKESHSRPFSLLPSEPCERPWEGPHTCPQPTVVYRTVYRQVVKTDHRQRLQCCHGFYESRGFCVPLCAHHHHHH
Peptide: EMI	biotin-LPSEPCERPWEGPHTCPQPTVVYRTVYRQVVKTDHRQRLQC
Peptide: EGF-like 1	biotin-CHGFYESRGFCVPLCAQECVHGRCVAPNQCQCVPGWRGDDCSSE
Peptide: EGF-like 2	biotin-CAPGMWGPQCDKPCSCGNNSSCDPKSGVCSCPSGLQPPNCLQP
Peptide: EGF-like 3	biotin-CTPGYYGPACQFRCQCHGAPCDPQTGACFCPAERTGPSCDVS
Peptide: EGF-like 4	biotin-CSQGTSGFFCPSTHSCQNGGVFQTPQGSCSCPPGWMGTICSLP
Peptide: EGF-like 5	biotin-CPEGFHGPNCSQECRCHNGGLCDRFTGQCRCAPGYTGDRCREE
Peptide: EGF-like 6	biotin-CPVGRFGQDCAETCDCAPDARCFPANGACLCEHGFTGDRCTDRL
Peptide: EGF-like 7	biotin-CPDGFYGLSCQAPCTCDREHSLSCHPMNGECSCLPGWAGLHCNES
Peptide: EGF-like 8	biotin-CPQDTHGPGCQEHCLCLHGGVCQATSGLCQCAPGYTGPHCASL
Peptide: EGF-like 9	biotin-CPPDTYGVNCSARCSCENAIACSPIDGECVCKEGWQRGNCSVP
Peptide: EGF-like 10	biotin-CPPGTWGFSCNASCQCAHEAVCSPQTGACTCTPGWHGAHCQLP
Peptide: EGF-like 11	biotin-CPKGQFGEGCASRCDCDHSDGCDPVHGRCQCQAGWMGARCHLS
Peptide: EGF-like 12	biotin-CPEGLWGVNCSNTCTCKNGGTCLPENGNCVCAPGFRGPSCQRS
Peptide: EGF-like 13	biotin-CQPGRYGKRCVPCKCANHSFCHPSNGTCYCLAGWTGPDCSQP
Peptide: EGF-like 14	biotin-CPPGHWGENCAQTCQCHHGGTCHPQDGSCICPLGWTGHHCLEG
Peptide:	biotin-CPLGTFGANCSQPCQCGPGEKCHPETGACVCPPGHSG

EGF-like 15	APC
Protein: LOXL2 full-his	MERPLCSHLCSCLAMLALLSPLSLAQYDSWPHYPEYFQQPAP EYHQPQAPANVAKIQLRLAGQKRKHSEGRVEVYYDGQWGT VCDDDFSIHAAHVVCRELGYVEAKSWTASSSYGKGEGPIWL DNLHCTGNEATLAACTSNGWGVTDCKHTEDVGVVCSDKRIP GFKFDNSLINQIENLNIQVEDIRIRAILSTYRKRTPVMEGYVEV KEGKTWKQICDKHWTAKNSRVVCGMFGFPGERTYNTKVYK MFASRRKQRYWPFSMDCTGTEAHISSCKLGPQVSLDPMKNV TCENGLPAVVSCVPGQVFSPDGPSRFRKAYKPEQPLVRLRGG AYIGEGRVEVLKNGEWGTVCDDKWDLVSASVVCRELGFGS AKEAVTGSRLGQGIGPIHLNEIQCTGNEKSIIDCKFNAESQGC NHEEDAGVRCNTPAMGLQKKLRLNGGRNPYEGRVEVLVER NGSLVWGMVCGQNWGIVEAMVVCRQLGLGFASNAFQETW YWHGDVNSNKVVMSGVKCSGTELSLAHCRHDGEDVACPQG GVQYGAGVACSETAPDLVLNAEMVQQTTYLEDRPMFMLQC AMEENCLSASAAQTDPTTGYRRLLRFSSQIHNNGQSDFRPKN GRHAWIWHDCHRHYHSMEVFTHYDLLNLNGTKVAEGHKAS FCLEDTECEGDIQKNYECANFGDQGITMGCWDMYRHDIDCQ WVDITDVPPGDYLFQVVINPNFEVAESDYSNNIMKCRSRYDG HRIWMYNCHIGGSFSEETEKKFEHFSGLLNNQLSPQHHHHH H
Protein: LOXL2 SR1-2-his	MERPLCSHLCSCLAMLALLSPLSLAQYDSWPHYPEYFQQPAP EYHQPQAPANVAKIQLRLAGQKRKHSEGRVEVYYDGQWGT VCDDDFSIHAAHVVCRELGYVEAKSWTASSSYGKGEGPIWL DNLHCTGNEATLAACTSNGWGVTDCKHTEDVGVVCSDKRIP GFKFDNSLINQIENLNIQVEDIRIRAILSTYRKRTPVMEGYVEV KEGKTWKQICDKHWTAKNSRVVCGMFGFPGERTYNTKVYK MFASRRKQRYWPFSMDCTGTEAHISSCKLGPQVSLDPMKNV TCENGLPAVVSCVPGQVFSPDGPSRFRKAYKPEQPLHHHHHH
Protein: LOXL2 SR1-3-his	MERPLCSHLCSCLAMLALLSPLSLAQYDSWPHYPEYFQQPAP EYHQPQAPANVAKIQLRLAGQKRKHSEGRVEVYYDGQWGT VCDDDFSIHAAHVVCRELGYVEAKSWTASSSYGKGEGPIWL DNLHCTGNEATLAACTSNGWGVTDCKHTEDVGVVCSDKRIP GFKFDNSLINQIENLNIQVEDIRIRAILSTYRKRTPVMEGYVEV KEGKTWKQICDKHWTAKNSRVVCGMFGFPGERTYNTKVYK MFASRRKQRYWPFSMDCTGTEAHISSCKLGPQVSLDPMKNV TCENGLPAVVSCVPGQVFSPDGPSRFRKAYKPEQPLVRLRGG AYIGEGRVEVLKNGEWGTVCDDKWDLVSASVVCRELGFGS AKEAVTGSRLGQGIGPIHLNEIQCTGNEKSIIDCKFNAESQGC NHEEDAGVRCNTHHHHHH

Supplemental Table 5. The sequences of recombinant proteins and peptides used in the study.
