# Supplementary material for: LOXL2-induced PEAR1 Ser891 phosphorylation suppresses CD44 degradation and promotes triple-negative breast cancer metastasis
Source: J Clin Invest. 2024 Aug 15;134(16):e177357. doi: 10.1172/JCI177357 (PMC11324313; doi:10.1172/JCI177357)

Full unedited blot for Figure [1D]

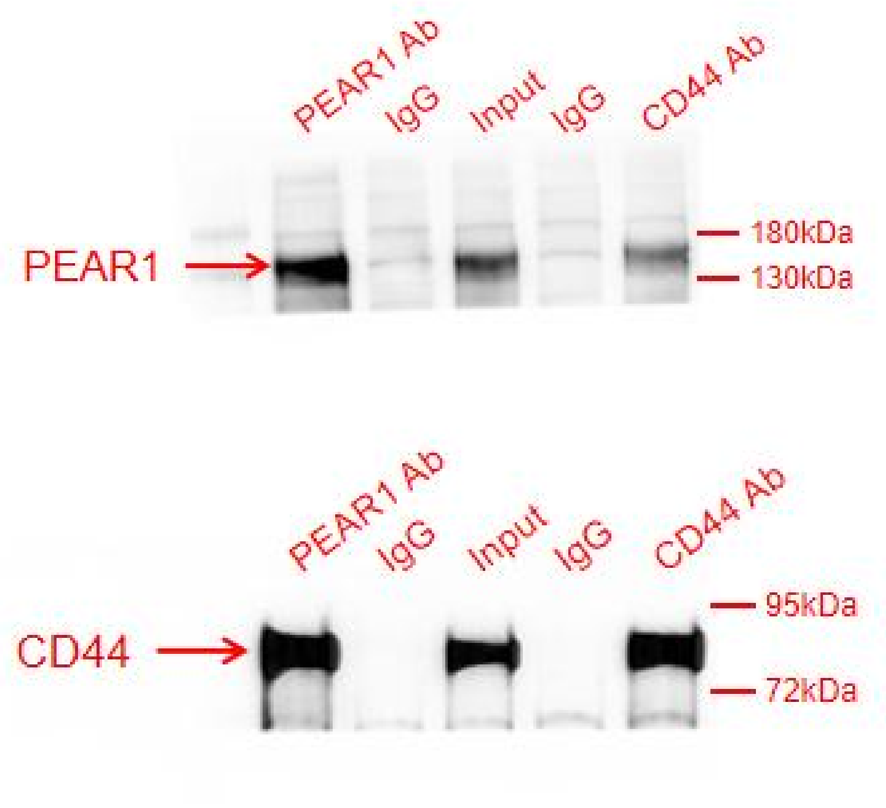

Full unedited blot for Figure [3A]

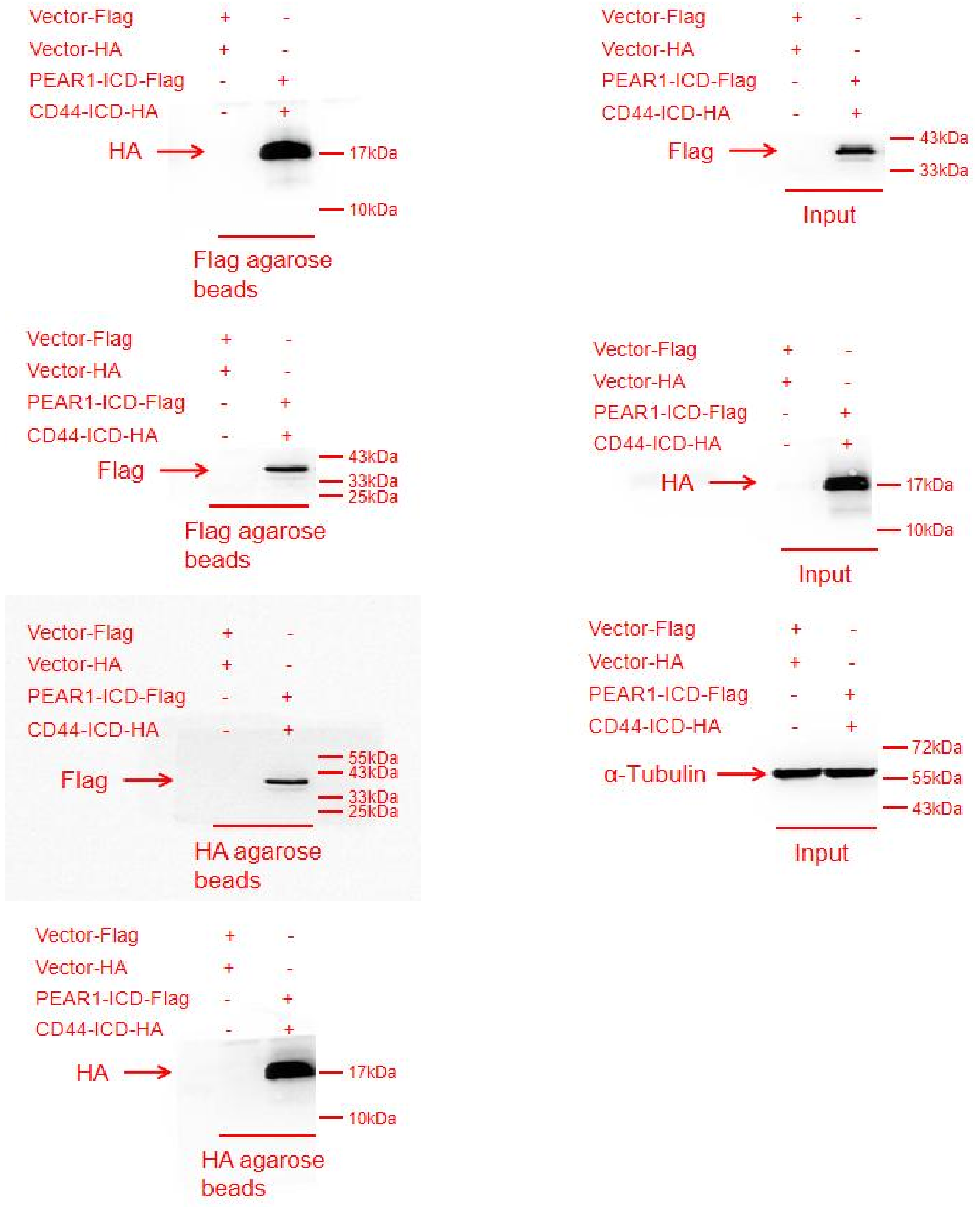

Full unedited blot for Figure [3B]

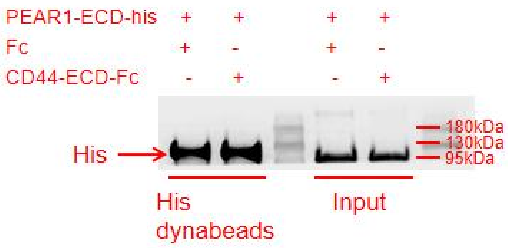

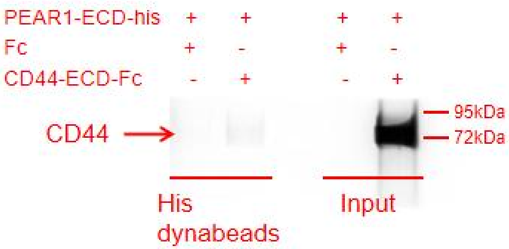

Full unedited blot for Figure [3D]

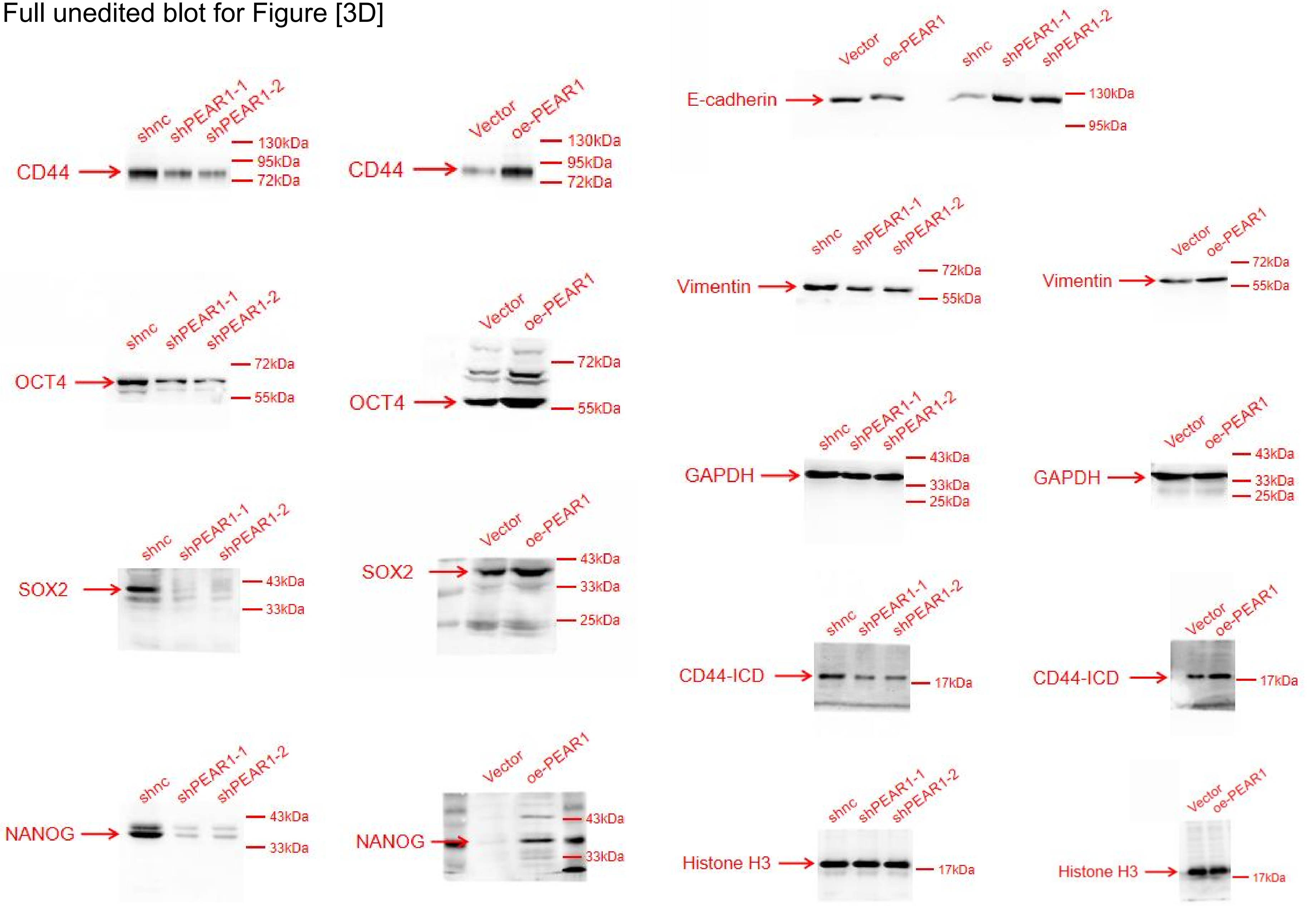

Full unedited blot for Figure [3F]

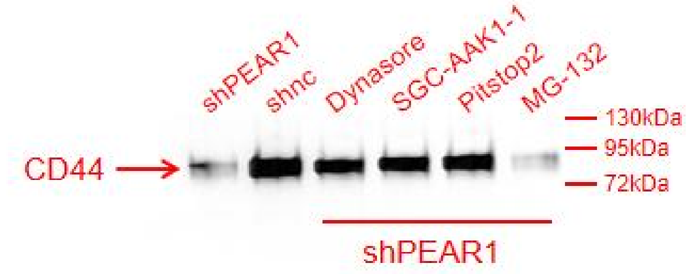

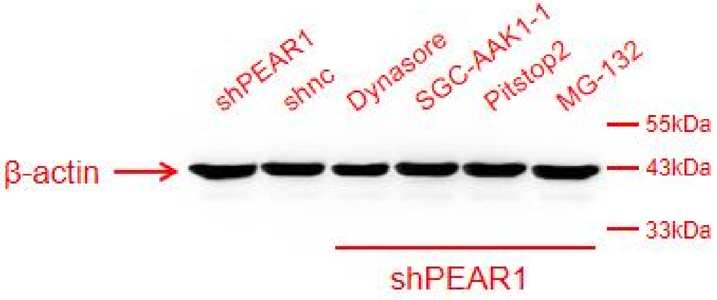

Full unedited blot for Figure [4A]

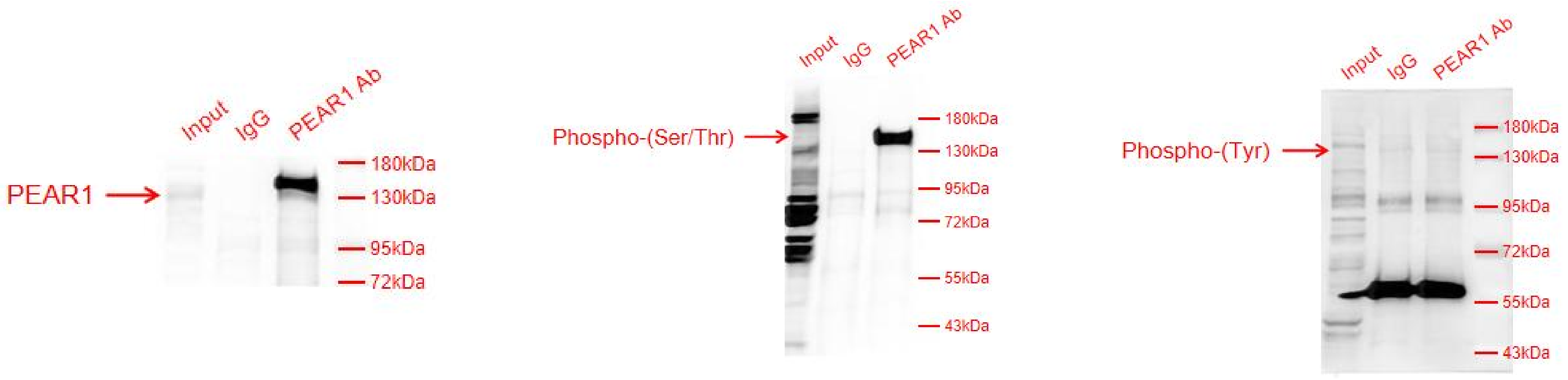

Full unedited blot for Figure [4B]

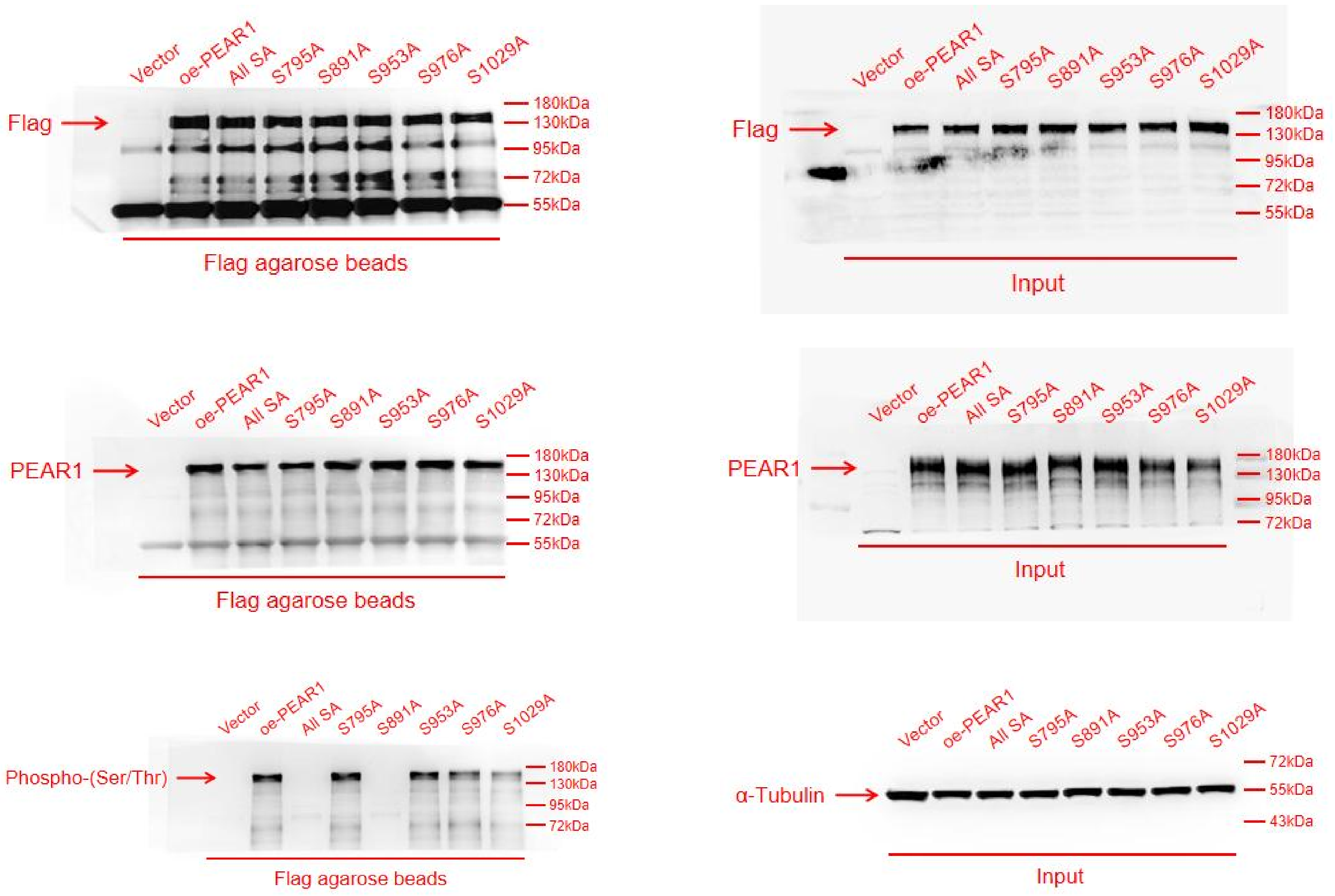

Full unedited blot for Figure [4C]

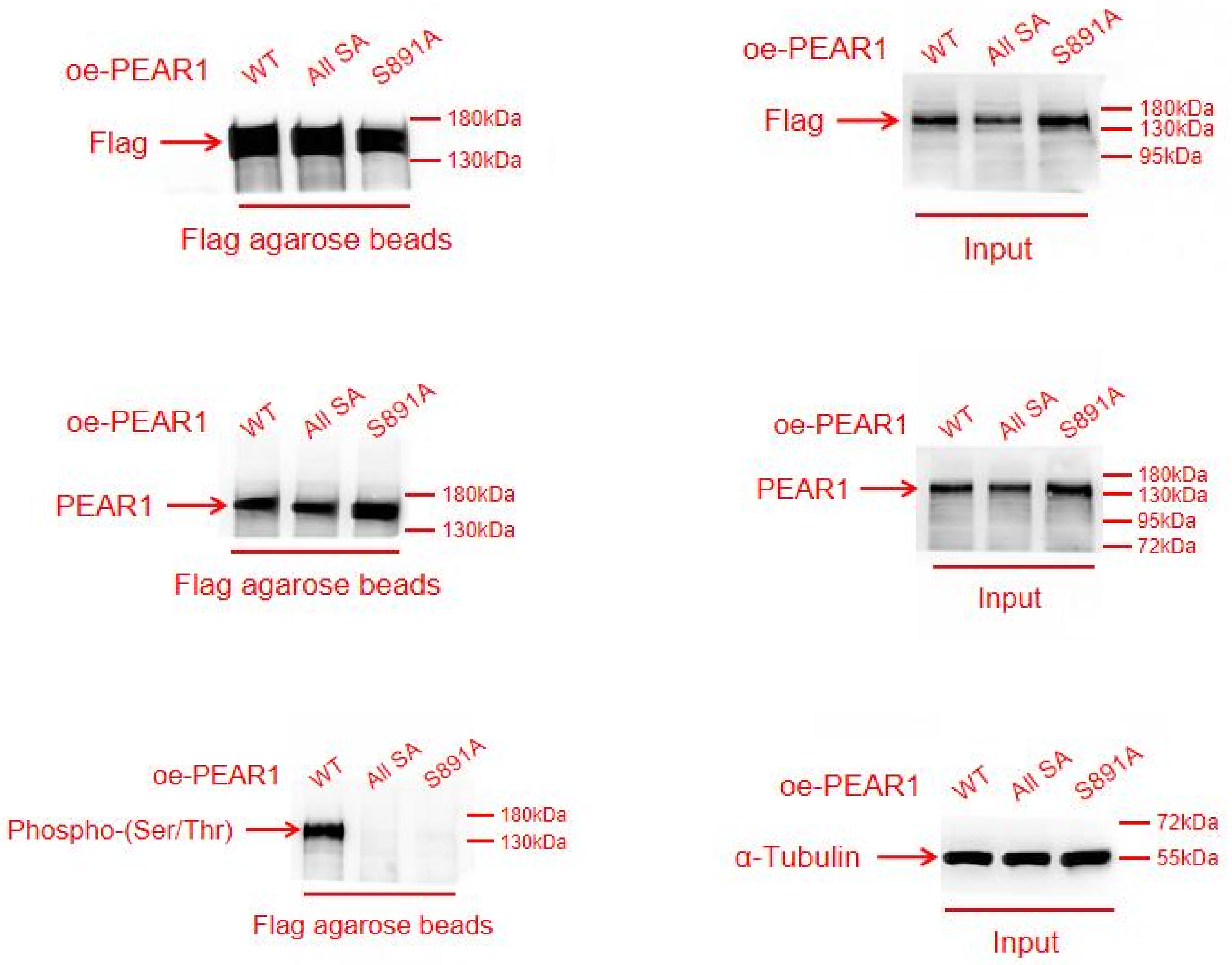

Full unedited blot for Figure [4D]

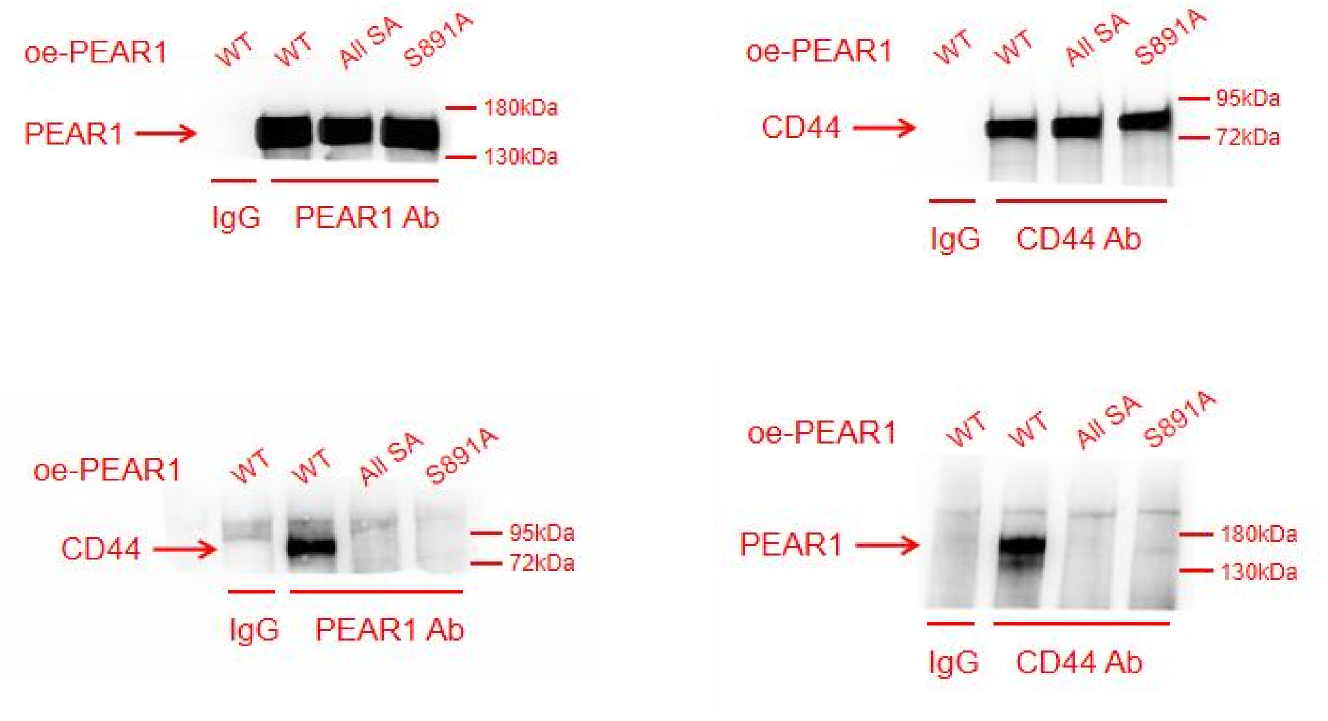

Full unedited blot for Figure [4E]

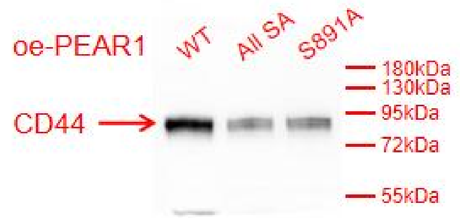

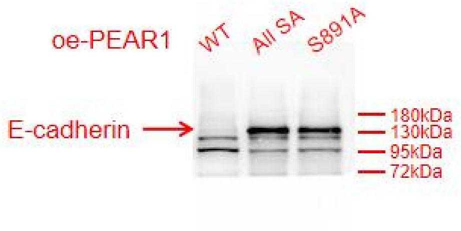

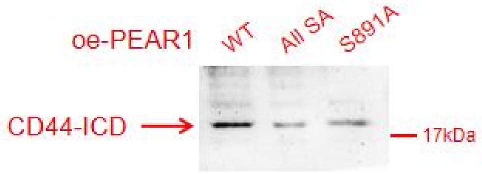

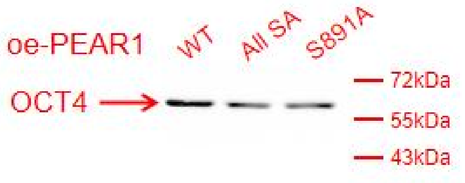

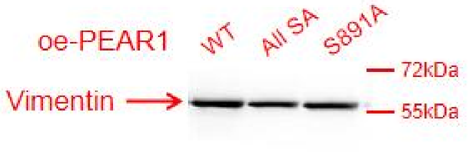

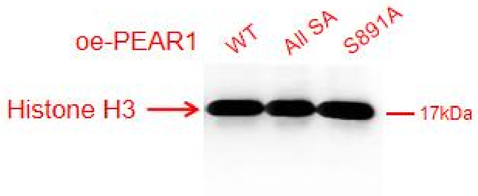

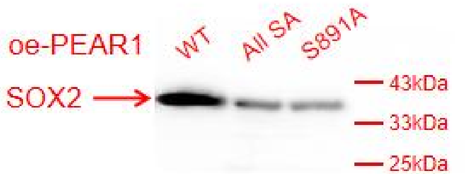

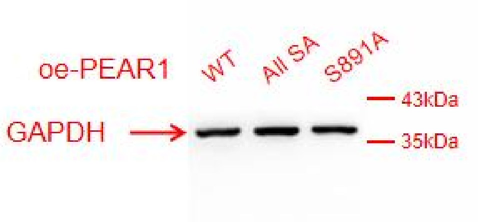

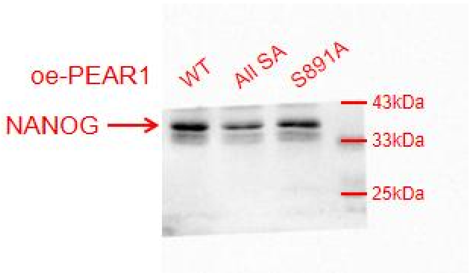

Full unedited blot for Figure [4F]

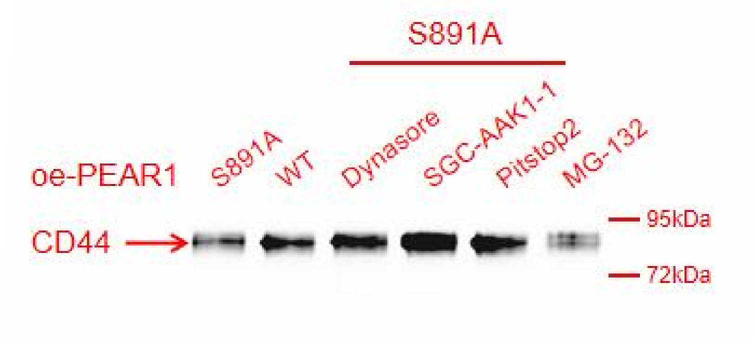

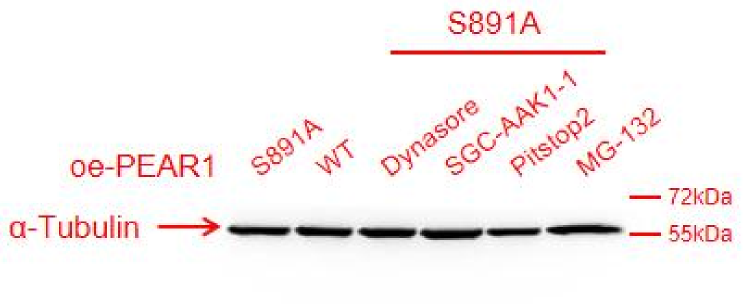

Full unedited blot for Figure [5A]

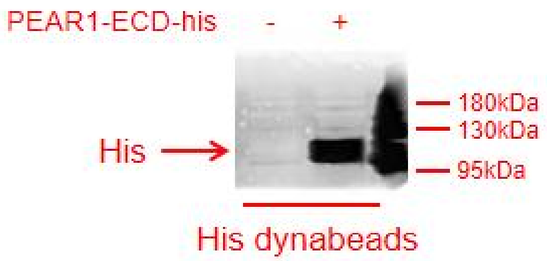

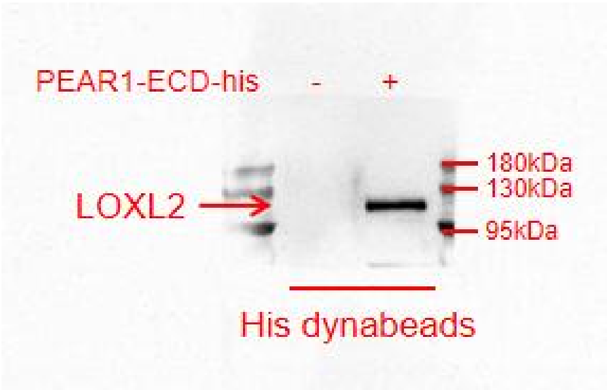

Full unedited blot for Figure [5E]

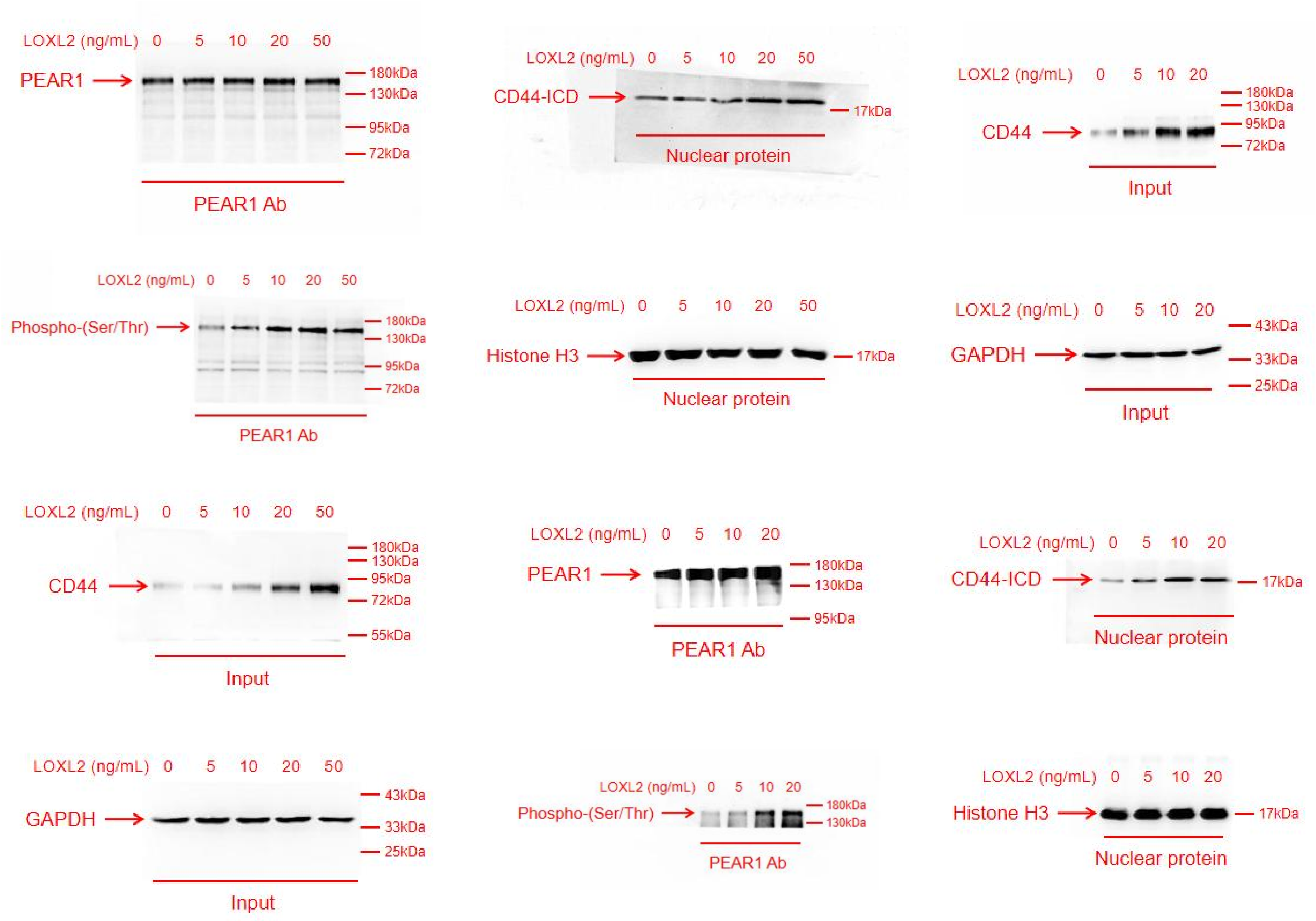

Full unedited blot for Figure [5I]

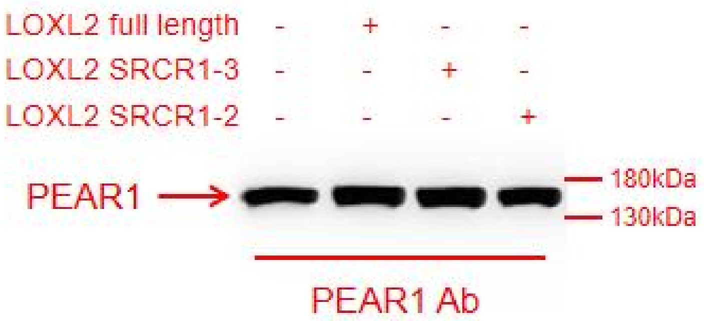

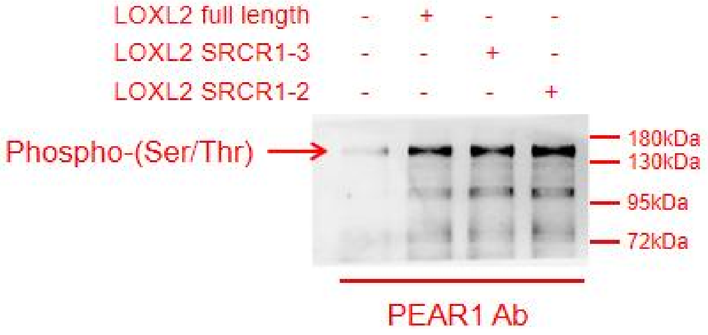

Full unedited blot for Figure [5J]

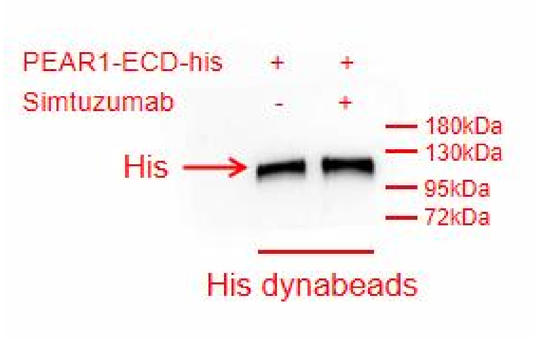

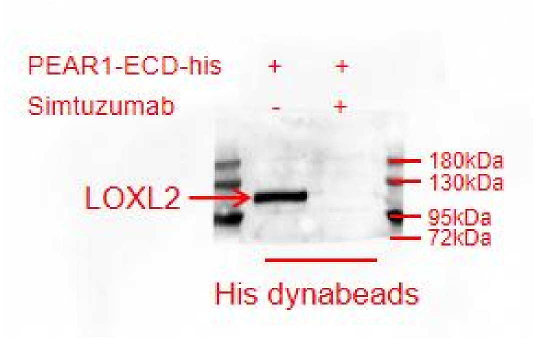

Full unedited blot for Figure [5M]

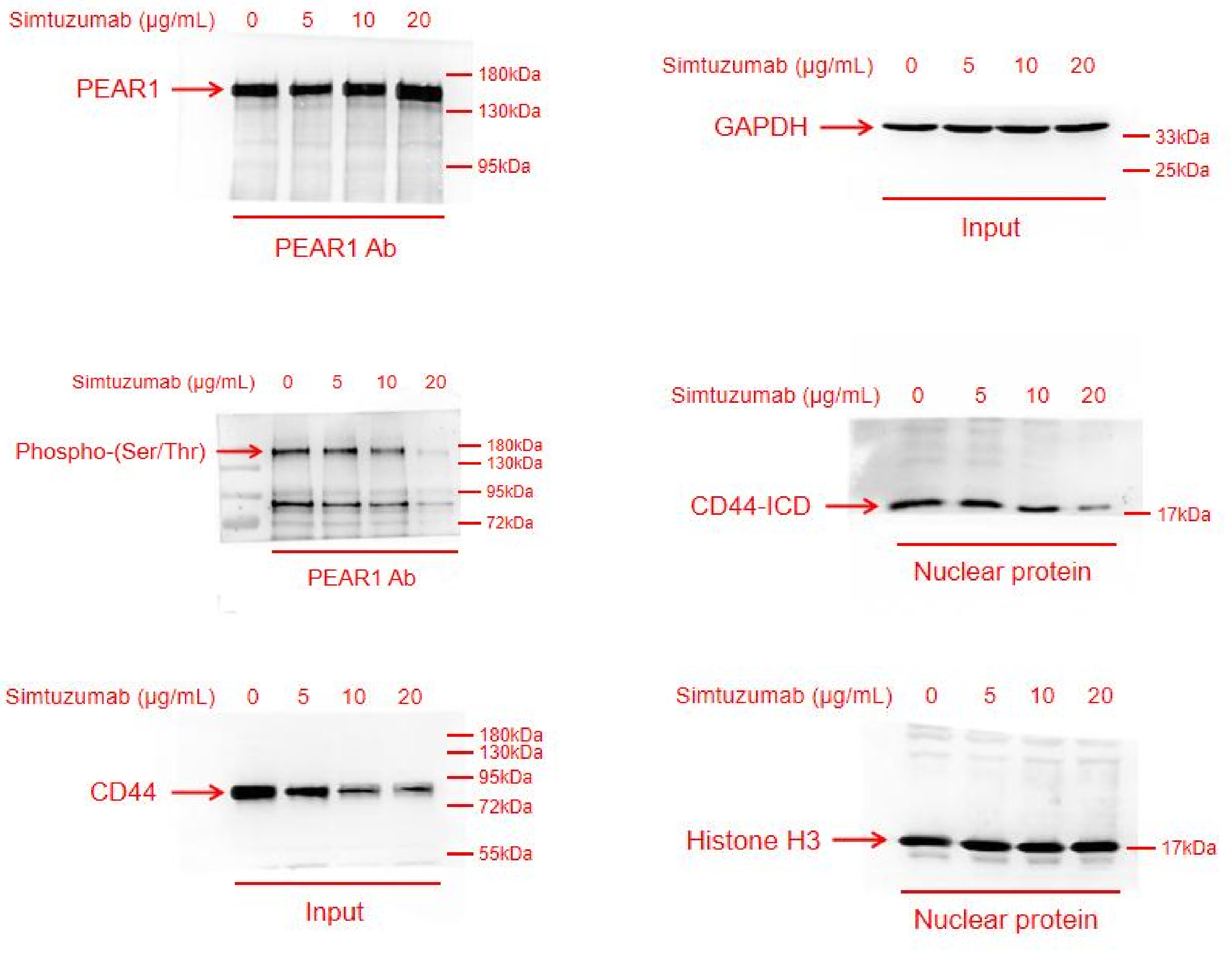

Full unedited blot for Figure [6E]

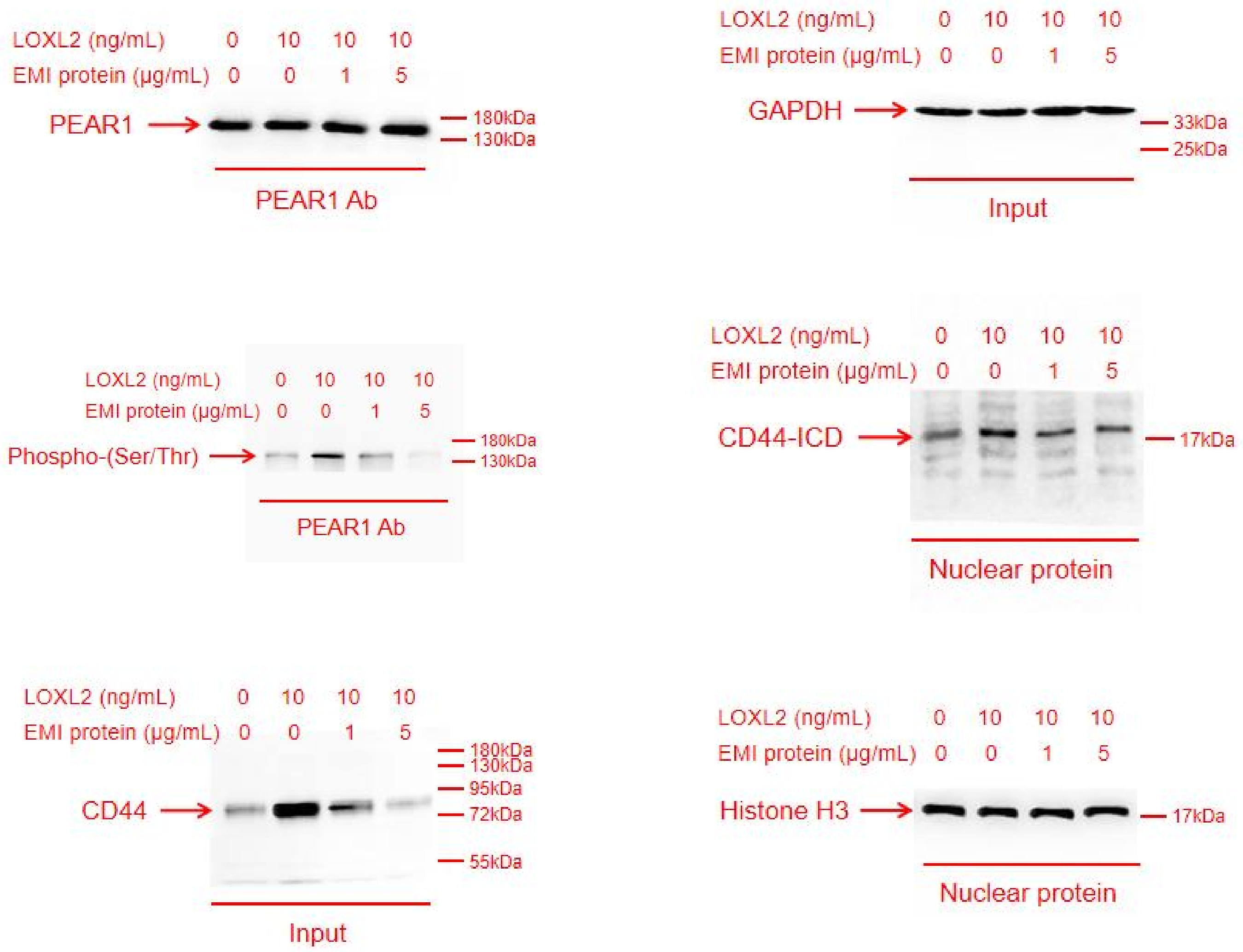

Full unedited blot for Figure [6H]

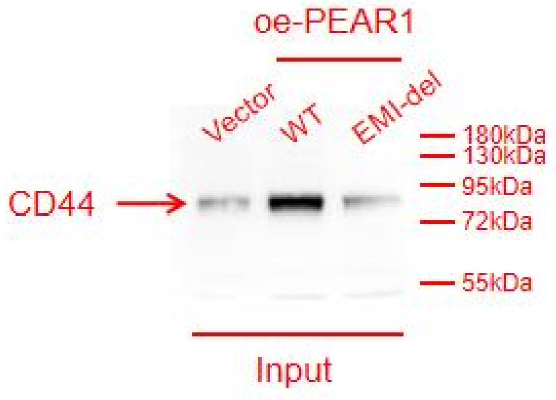

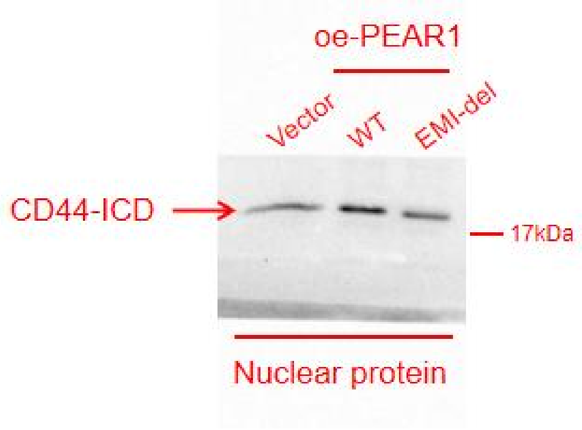

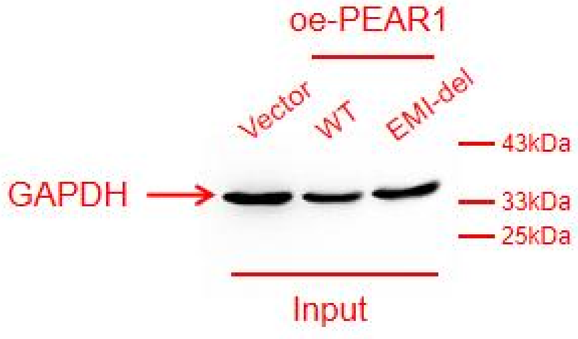

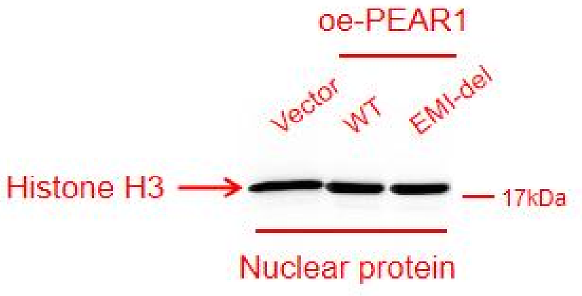

Full unedited blot for Figure [6K]

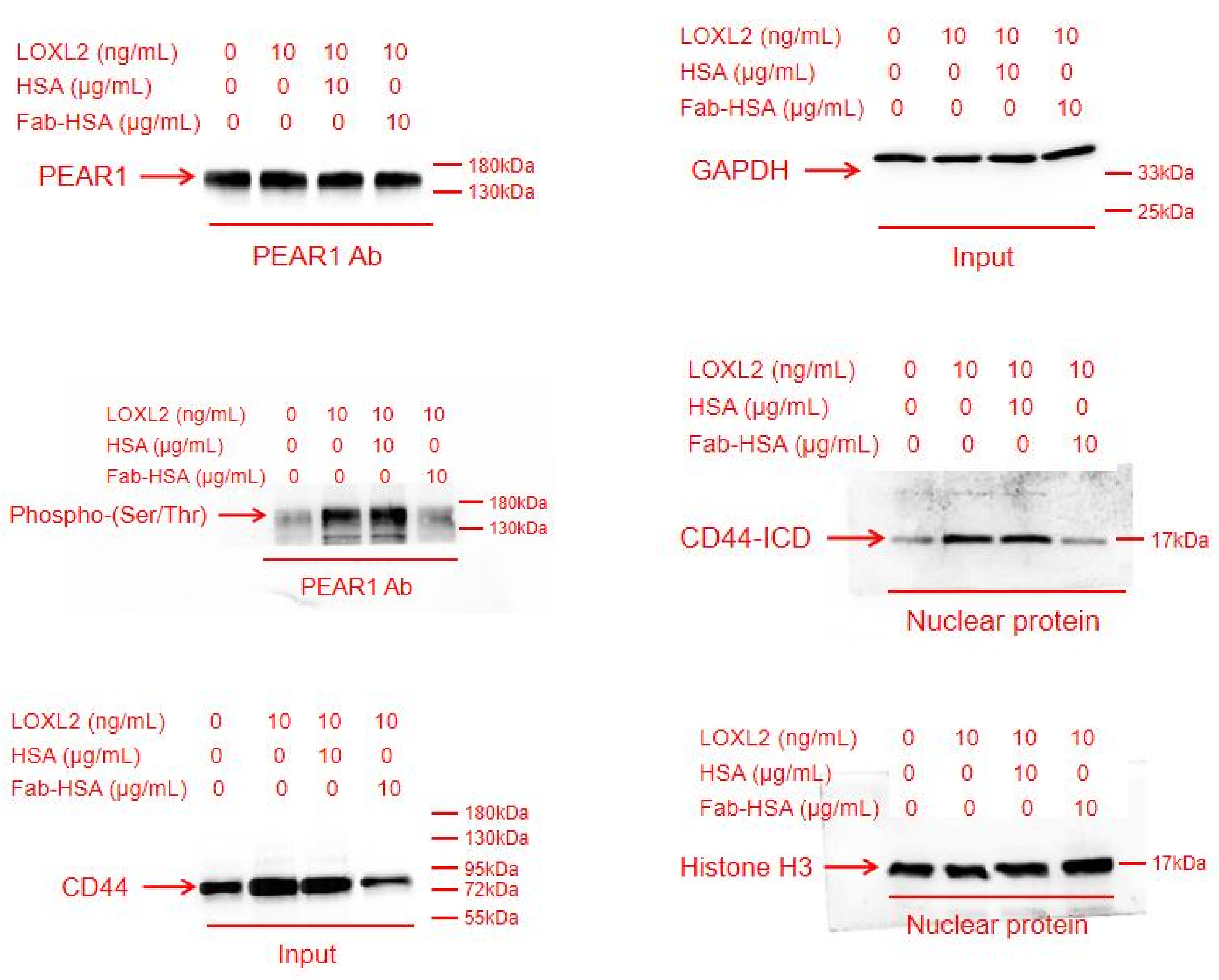

Full unedited blot for Figure [S2A]

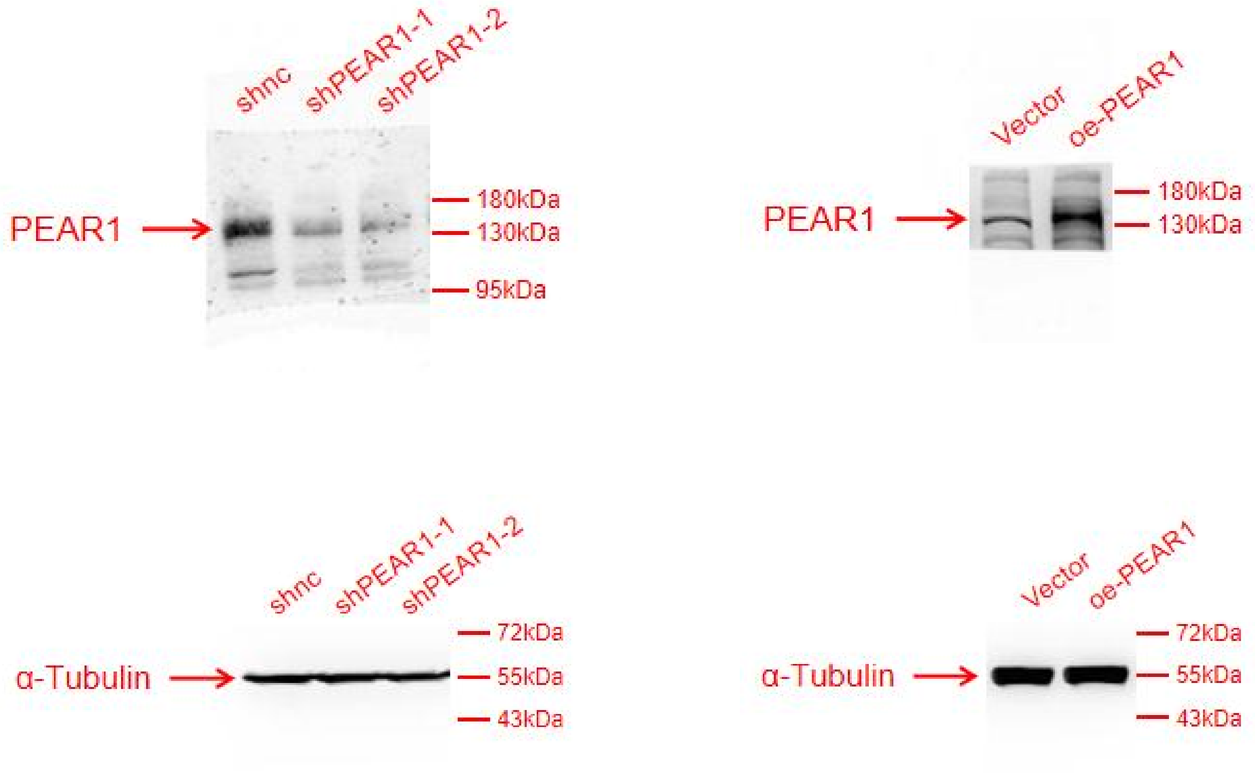

Full unedited blot for Figure [S2B]

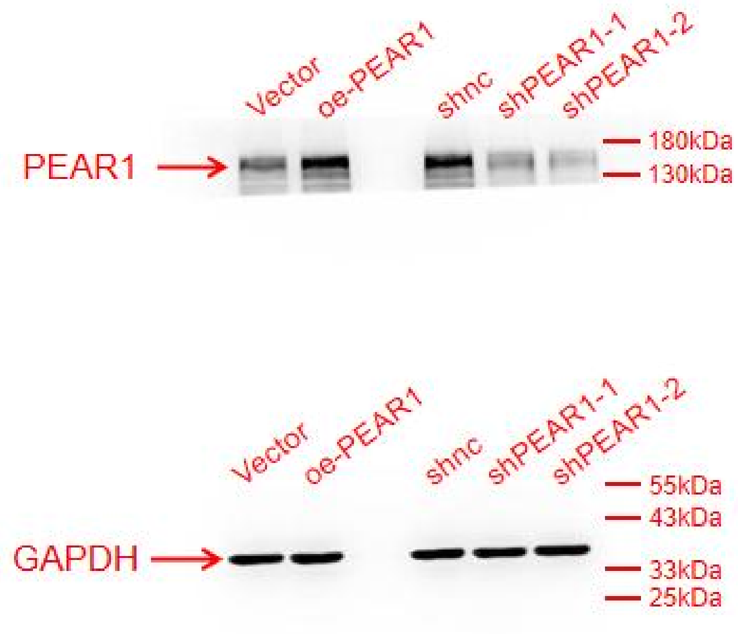

Full unedited blot for Figure [S2C]

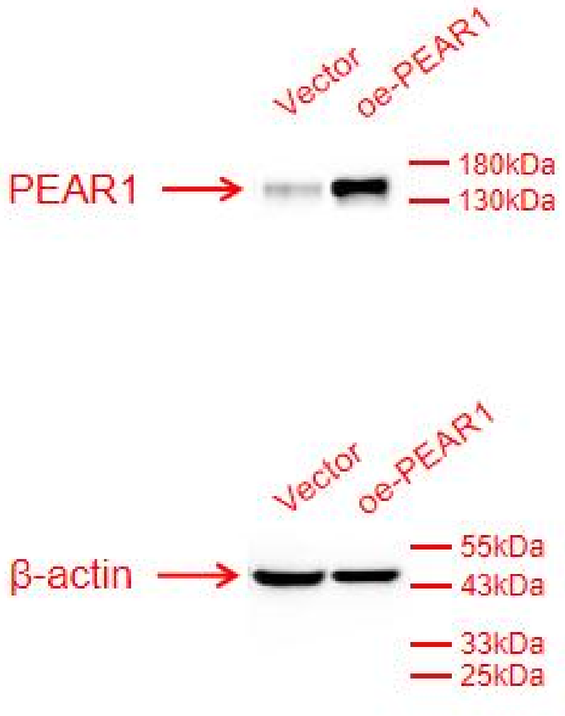

Full unedited blot for Figure [S3A]

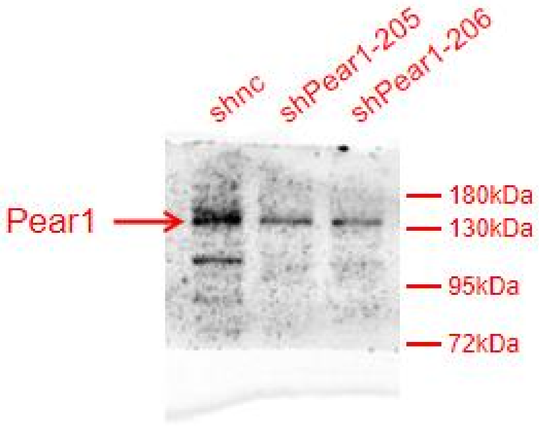

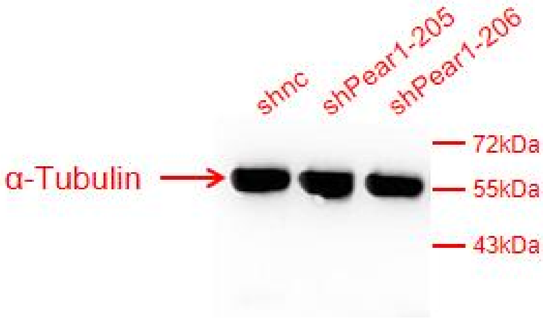

Full unedited blot for Figure [S4B]

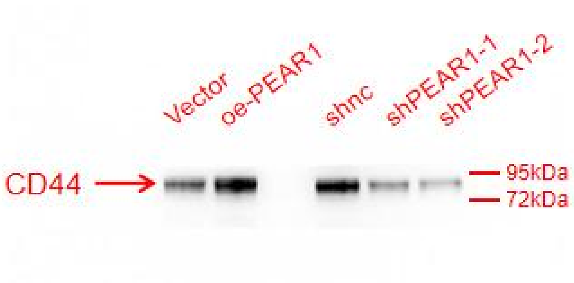

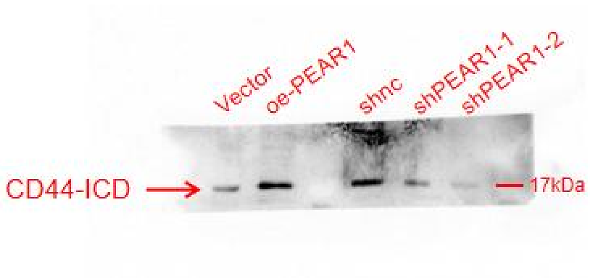

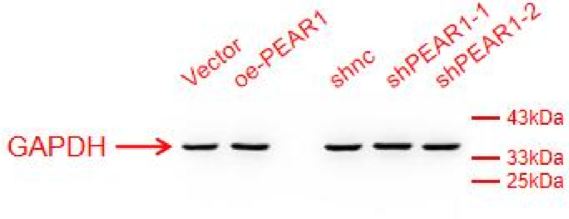

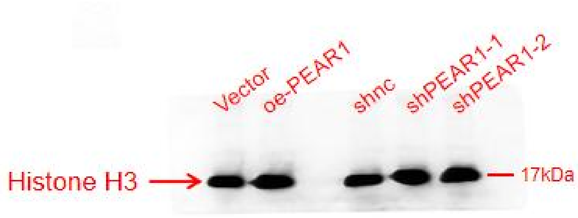

Full unedited blot for Figure [S4D]

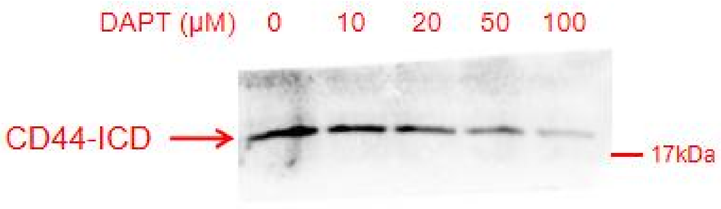

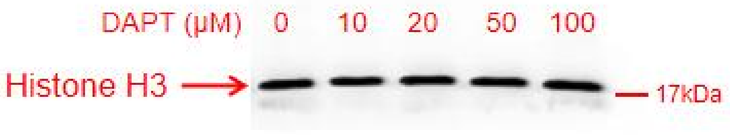

Full unedited blot for Figure [S5A]

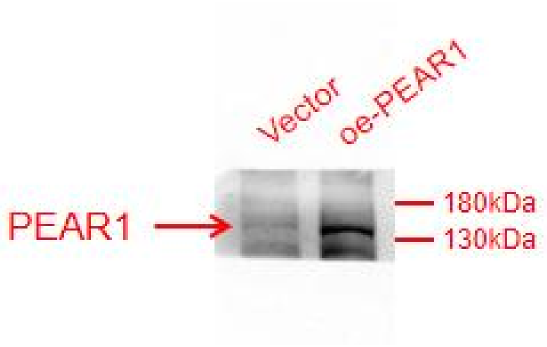

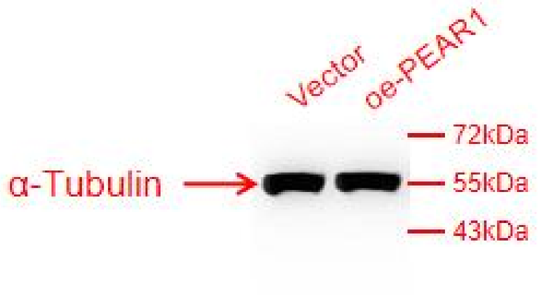

Full unedited blot for Figure [S5E]

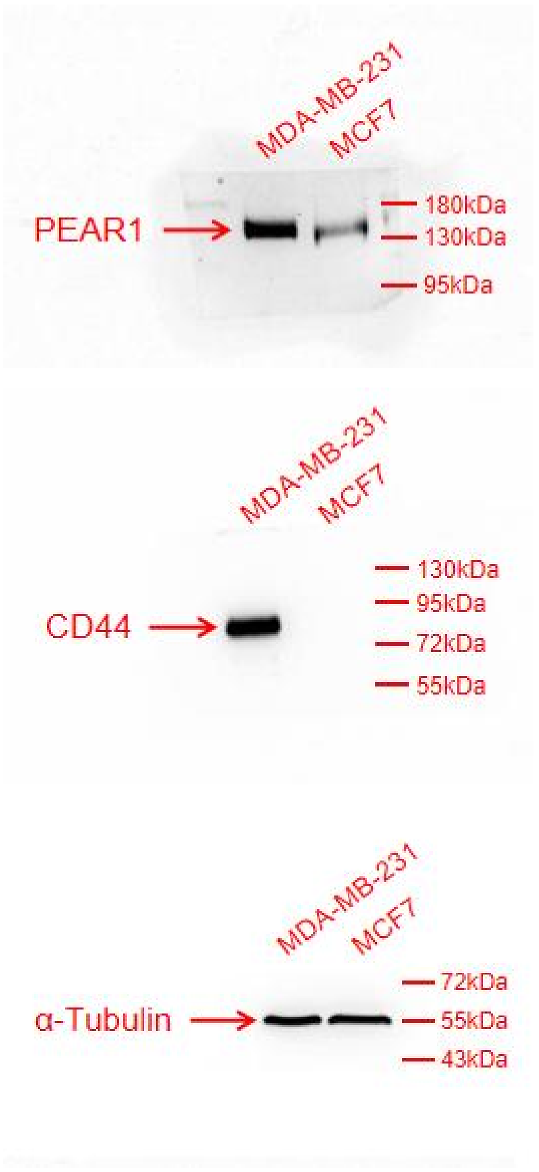

Full unedited blot for Figure [S5F]

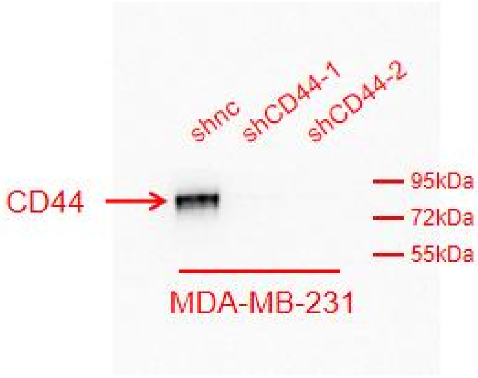

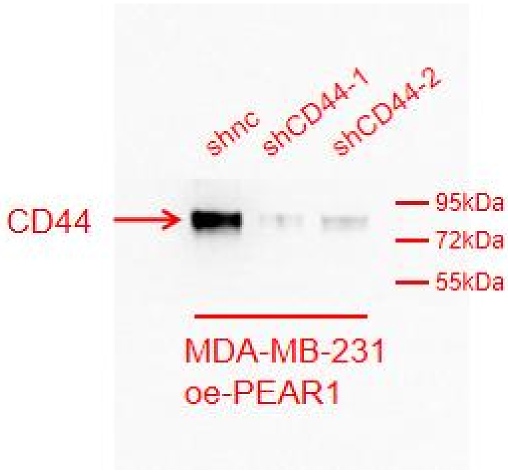

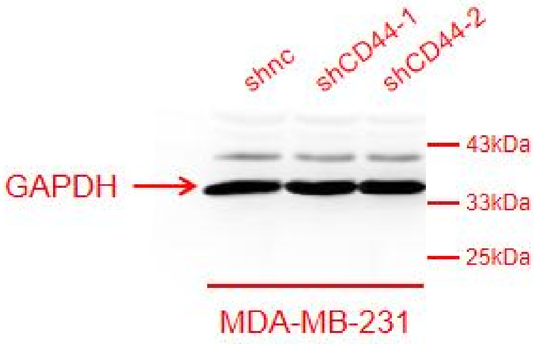

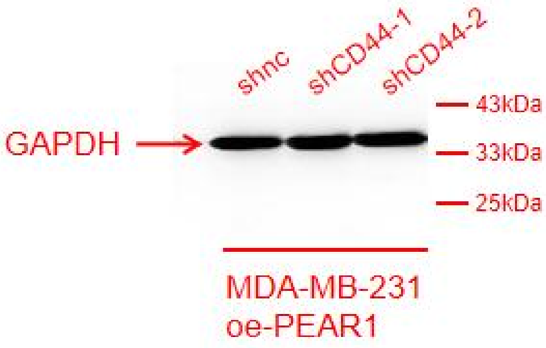

Full unedited blot for Figure [S6H]

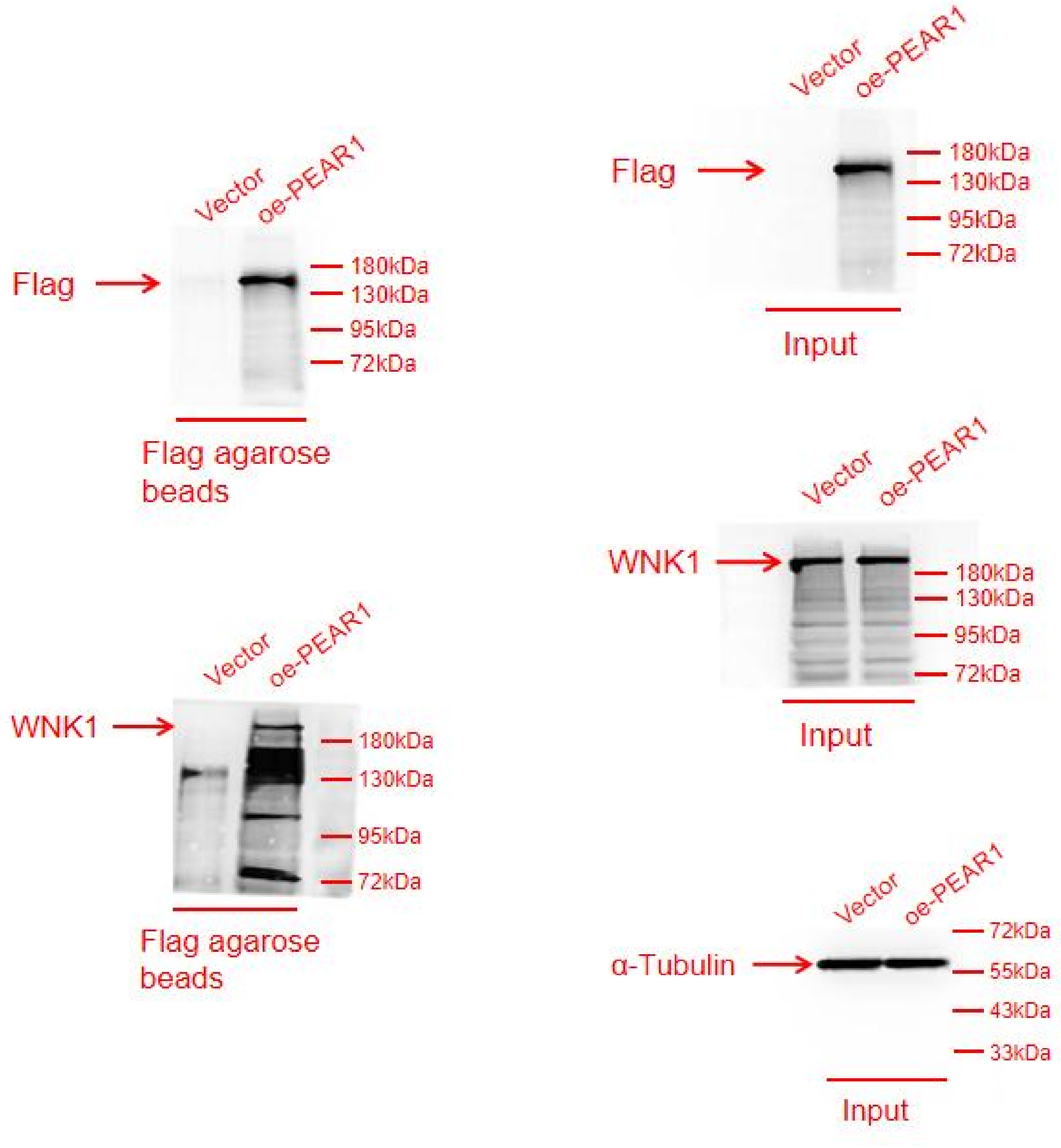

Full unedited blot for Figure [S6I]

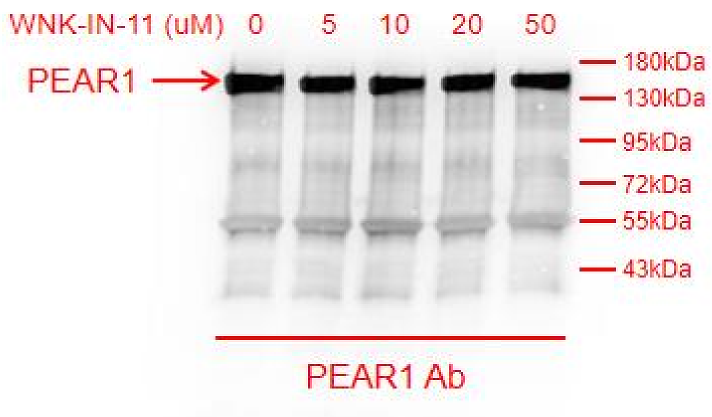

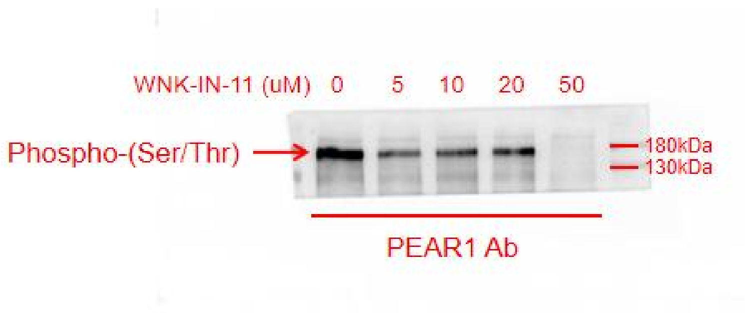

Full unedited gel for Figure [S7A]

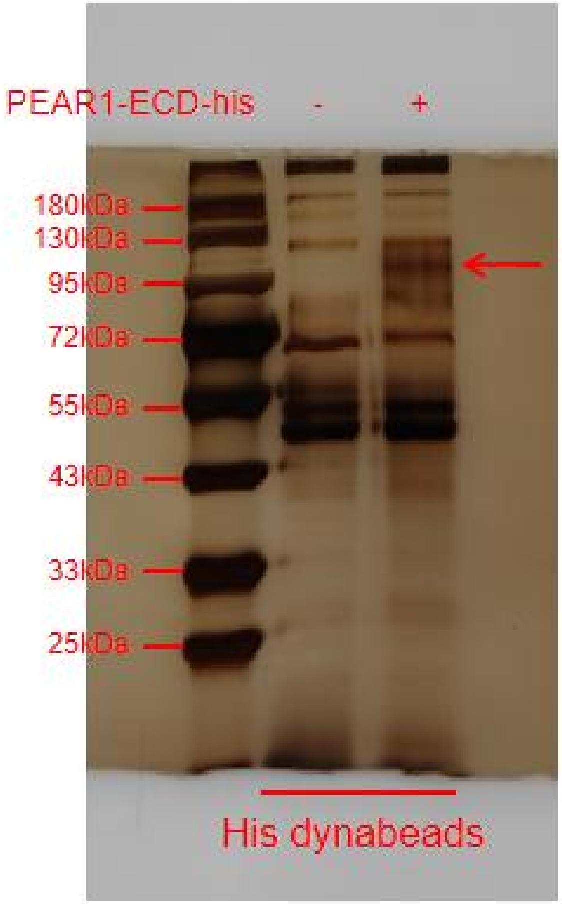

Full unedited blot for Figure [S9C]

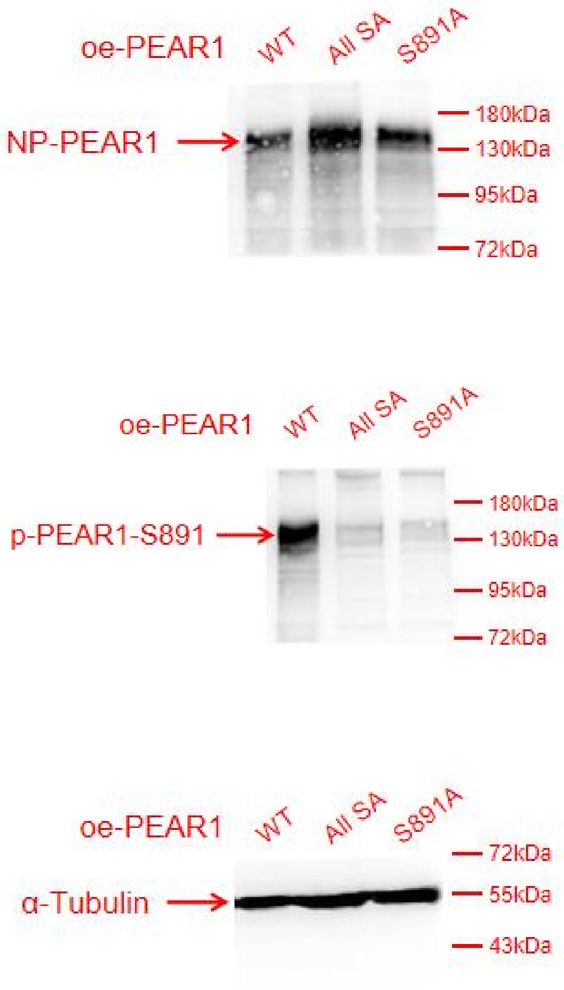

Full unedited blot for Figure [S9D]

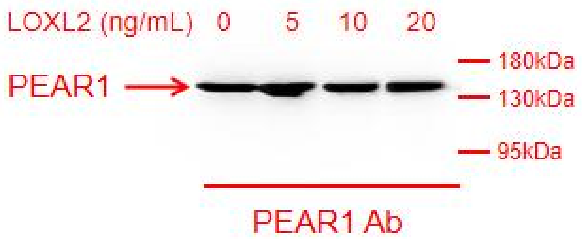

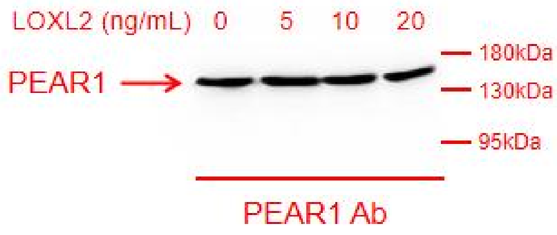

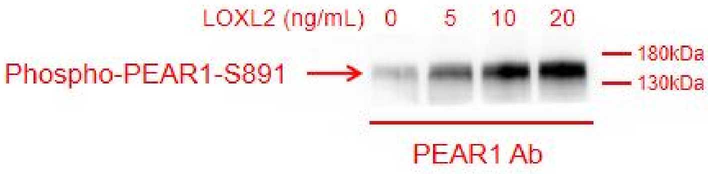

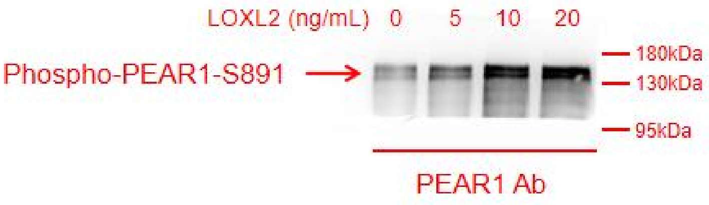

Supplement: Unedited blot and gel images [file jci-134-177357-s184.pdf]
